# Proteomic Analysis of Mycelial Exudates of *Ustilaginoidea virens*

**DOI:** 10.3390/pathogens10030364

**Published:** 2021-03-18

**Authors:** Haining Wang, Xiaohe Yang, Songhong Wei, Yan Wang

**Affiliations:** 1 Department of Plant Protection, Shenyang Agricultural University, Shenyang 110866, China; 2018200123@stu.syau.edu.cn; 2 Jiamusi Branch of Heilongjiang Academy of Agricultural Sciences, Jiamusi 154007, China; yxh@haas.cn

**Keywords:** *Ustilaginoidea virens*, exudate, proteomic

## Abstract

Rice false smut (RFS) disease, which is caused by *Ustilaginoidea virens*, has been widespread all over the world in recent years, causing irreversible losses. Under artificial culture conditions, exudates will appear on colonies of *U. virens* during the growth of the hyphae. Exudation of droplets is a common feature in many fungi, but the functions of exudates are undetermined. As the executors of life functions, proteins can intuitively reflect the functions of exudates. Shotgun proteomics were used in this study. A total of 650 proteins were identified in the exudate of *U. virens*, and the raw data were made available via ProteomeXchange with the identifier PXD019861. There were 57 subcategories and 167 pathways annotated with Gene Ontology (GO) classification and Kyoto Encyclopedia of Genes and Genomes (KEGG) pathway analysis, respectively. Through protein–protein interaction (PPI) network analysis, it was found that 20 proteins participated in the biosynthesis of secondary metabolites. Two separate PPI analyses were performed for carbon metabolism and microbial metabolism in diverse environments. After comparing and annotating the functions of proteins of the exudate, it was speculated that the exudate was involved in the construction and remodeling of the fungal cell wall. Pathogenicity, sporulation, and antioxidant effects might all be affected by the exudate.

## 1. Introduction

Rice false smut (RFS) is a kind of fungal disease that transforms panicles and spikelets into greenish spore balls and is caused by *Ustilaginoidea virens* (Cooke) Takah or *Villosiclava virens* (Nakata) E. Tanaka et C. Tanaka [1,2]. The disease was first discovered in India in the 1870s [3], and is now widely distributed in all major rice-producing areas of the world, including more than 40 countries [4]. Although there are many disease-resistant rice varieties, the majority of the cultivated varieties are still susceptible to false smut [5,6]. *U. virens* (anamorph) belongs to Ascomycota, *Ustilaginoidea*, and produces asexual chlamydospores, while *V*. *virens* (teleomorph) belongs to Ascomycetes, *Villosiclava*, and produces sexual ascospores. There are no visible symptoms in early stages of the disease; it cannot be observed until the rice grains are replaced by ball-shaped fungal mycelia [7]. When this characteristic has occurred, it is already too late to prevent the disease [8]. RFS is spread through spores, and the sori of *U. virens* are located in the palea and lemma [9]. In late autumn, the relatively low temperatures can induce sclerotial differentiation and formation and increase the number of sclerotia [10]. Sclerotia and chlamydospores play important roles in the life cycle of the fungus, as they contribute to primary infections in the following year [11]. RFS seriously affects the quantity and quality of rice [12], and the toxic secondary metabolites of fungi also poison humans and livestock [13,14,15]. RFS is already one of the main diseases in rice paddies in China [11]. At present, some chemical fungicides are commonly used for the prevention and treatment of this disease [7].

Under artificial cultivation conditions, the colonies were found to be slow growing, smooth, and between white and yellowish on their surfaces, with a slightly bulging center. The aerial hyphae are well developed and have a fine and velvety texture. During the artificial cultivation process, macroscopic exudates were observed, which is a common feature in many kinds of fungi [16,17,18,19,20]. Liang et al. [16] reported 56 proteins in the exudate from *Sclerotinia sclerotiorum* and classified the proteins into six functional categories by searching the available databases. In the exudates of *S*. *ginseng*, there were 59 proteins that satisfied the identified conditions. According to a Gene Ontology (GO) analysis, these 59 proteins were classified into six groups: carbohydrate metabolism, oxidation–reduction process, transport and catabolism, amino acid metabolism, other functions, and unknown proteins [17]. Through comparison with the transcriptome of *Cercospora armoraciae*, GO, Kyoto Encyclopedia of Genes and Genomes (KEGG), and Cluster of Orthologous Groups (COG/KOG) bioinformatics analyses were conducted on the 576 identified proteins of exudates of *C*. *armoraciae* [18]. Exudates contain many components, including organic acids, lipids, soluble sugars, enzymes, amino acids, cations, fatty acids, and free ammonia [19,20,21,22,23,24]. The function of exudates is also a popular topic for many researchers, as it may have close relationships with its components [17]. The oxalic acid and free ammonia in exudates may play a role in pathogenesis [22,25]. The presence of amino acids, sugars, and organic acids is thought to be responsible for the sclerotial resistance against microbial attacks [20]. The exudates can maintain the internal physiological balance during the growth of *S*. *sclerotiorum* and *S*. *trifoliorum*, mainly by eliminating excess soluble carbohydrates through the exudation of droplets [26]. The exudates of different fungi differ in color, which is determined by the degree of phenolic acid oxidation [27]. The exudate of *Rhizoctonia solani* at a concentration of 10,000 ppm also has a varied and significant phytotoxic effect on the decreases in the chlorophyll contents of *Abutilon theophrastii*, *Avena sterilis*, *Echinochloa crus-galli*, and *Poa pratensis* [20]. Many fungi in different growth stages are accompanied by the production of exudates; the compositions and functions of these exudates are not entirely clear, but they play irreplaceable roles in the growth, development, survival, and pathogenesis of the pathogen.

At present, this disease has reached epidemic status [28], but there has been relatively little progress in research on RFS. First, the occurrence of RFS has historically not been serious, so it has not attracted enough attention. Secondly, the velvety false smut ball is the only visible symptom in the field, and its infection process is not clear. Thirdly, the false smut ball has many competitors, it is difficult to isolate and inoculate, and it needs a long time to grow in an artificial culture medium, which is not conducive to studying the pathogen [29,30]. This study took the biological characteristics of *U. virens* as an entry point. The study of the protein components of the exudates of *U. virens* is helpful in order to better understand the occurrence and pathogenic mechanisms of the pathogen and to provide a theoretical basis for the control of RFS.

## 2. Results

### 2.1. Colony and Exudate Morphology of U. virens

**Figure 1:**
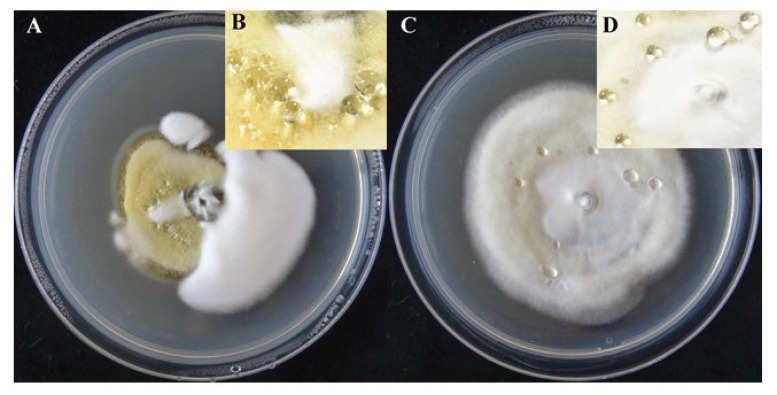
The colony morphology and the exudation of droplets of *Ustilaginoidea virens* under artificial culture conditions after 25 d. (**A**) The colony morphology of *U. virens* with yellowish and white hyphae. (**B**) The exudate from the yellowish hyphae of *U. virens*. (**C**) The colony morphology of *U. virens* with white hyphae. (**D**) The exudate from the white hyphae of *U. virens*.

The colonies of *U. virens* grew slowly on a potato sucrose agar (PSA) medium with a slightly raised center. The aerial hyphae were well developed and the texture was fine and velvety. The front color of the colonies was white or yellowish and became slightly darker in later stages. The back color of the colonies was white, yellow, yellow-green, or dark green. Exudates were collected from the hyphae of the *U. virens* after culture treatment for 25 d. The exudates were generally produced in the yellowish colonies or the junction between the white and yellowish colonies (Figure 1A,B), while a few were produced in the white colony (Figure 1C,D). The droplets were spherical, colorless, and transparent (Figure 1B,D).

### 2.2. Protein Identification

There were 650 proteins identified in the exudate (Supplementary Table S1); they were annotated by searching the UniProtKB *U. virens* database, and the raw data were made available via ProteomeXchange with the identifier PXD019861 (http://proteomecentral.proteomexchange.org accessed on 1 March 2021). These proteins were annotated in different groups and had different biological functions (Table 1). Some proteins in the exudates were related to the activities of the cell wall, including the organization, formation, and remodeling of the fungal cell wall. There were also some proteins that were closely related to fungal hyphae growth and spore formation. One protein identified in the exudate was associated with spore germination. The proteins in the exudate were not only related to the growth and development of the pathogen, but also had the function of regulating internal physiological balance by maintaining cellular redox homeostasis. In addition, it was determined from the identified proteins that the exudates were also involved in certain basic metabolic pathways, such as the carbohydrate metabolic process, tricarboxylic acid cycle, translation process, amino acid metabolism process, fatty acid biosynthetic process, glycolytic process, mannose metabolism process, and protein folding process. The identified proteins were also used to predict certain functions of the exudates. The exudates contained pathogenesis-related proteins, and some proteins were involved in the catabolism of chitin and cellulose. Some proteins in the exudates were involved in dealing with adversities, such as the response to oxidative stress, the cellular response to osmotic stress, the oxidation–reduction process, the defensive response to fungi, and the response to toxic substances.

### 2.3. GO Classification

**Figure 2:**
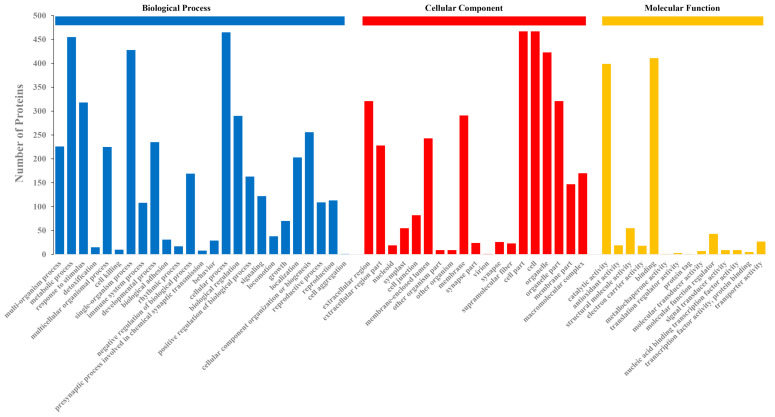
Gene Ontology (GO) classification of the identified exudate proteins from *U. virens*. Biological processes, cellular components, and molecular functions were the three main categories of GO terms. The values of the bars for each process represent the number of annotated proteins participating in that process.

Through GO analysis, a total of 57 GO terms contained by 508 proteins (78%) were classified. Among the 57 GO terms, 24 subcategories belonged to biological processes, 19 subcategories belonged to cellular components, and 14 subcategories were classified as molecular functions (Figure 2). In the biological processes, the metabolic processes, cellular processes, and single-biological processes occupied the first three terms in this category group. With these GO terms, it was also found that the proteins in the exudate were related to the whole life cycle of *U. virens*, including growth, development, reproduction, and cell killing. The cell, cell part, and organelle were the three most abundant terms in the cellular component category. In the molecular function category, some exudate proteins were classified into the antioxidant activity group. In this group, catalytic activity and binding accounted for large proportions.

### 2.4. KEGG Pathway Analysis

**Figure 3:**
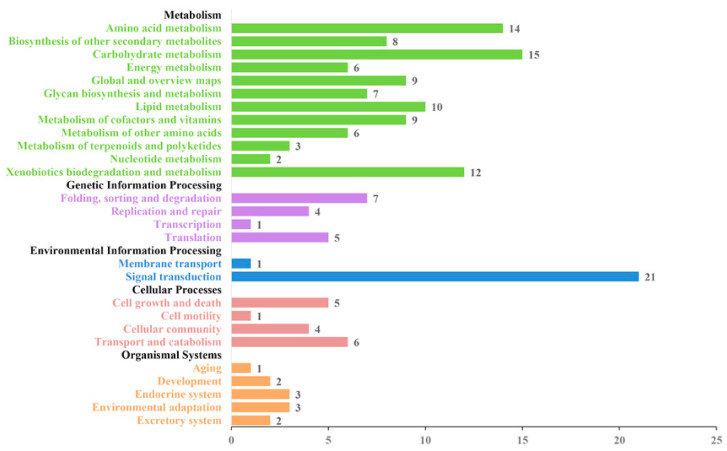
Kyoto Encyclopedia of Genes and Genomes (KEGG) pathway analysis of the identified exudate proteins from *U. virens*. The abscissa values represent the numbers of pathways in the subgroups of the secondary class level.

The 650 proteins were annotated in KEGG pathways, and 167 pathways were selected for KEGG analysis (Figure 3). At the primary class level, these 167 pathways were classified into five groups: metabolism, genetic information processing, environmental information processing, cellular processes, and organismal systems. At the secondary class level, a total of 27 subgroups were annotated. The metabolism group, which contained the largest number of subgroups, embodied the multiple metabolic pathways of proteins in the exudates, including amino acid metabolism, biosynthesis of secondary metabolites, energy metabolism, carbohydrate metabolism, and lipid metabolism, for a total of 12 pathways. The four pathways of folding, sorting and degradation, replication and repair, and transcription and translation belonged to the genetic information processing group. Membrane transport and signal transduction belonged to the environmental information processing group. In the cellular process group there were four pathways, while some proteins of the exudates were annotated in the cell growth and death pathways. In the organismal system group, five selected pathways were found: aging, development, endocrine system, environmental adaptation, and excretory system.

### 2.5. Protein–Protein Interaction (PPI) Network Analysis

**Figure 4:**
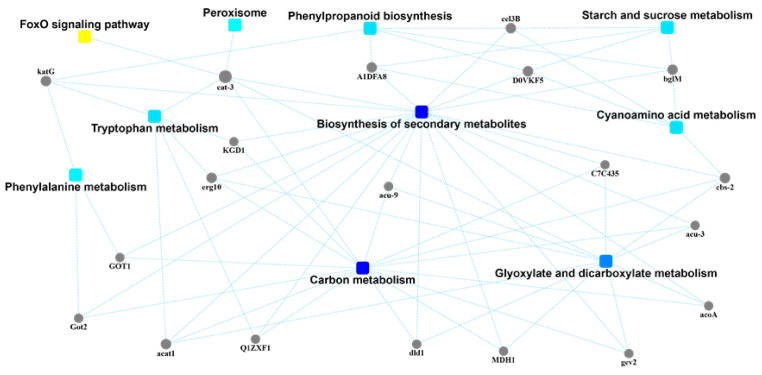
Protein–protein interaction (PPI) network analysis of the identified exudate proteins from *U. virens* that were related to the biosynthesis of secondary metabolites.

**Figure 5:**
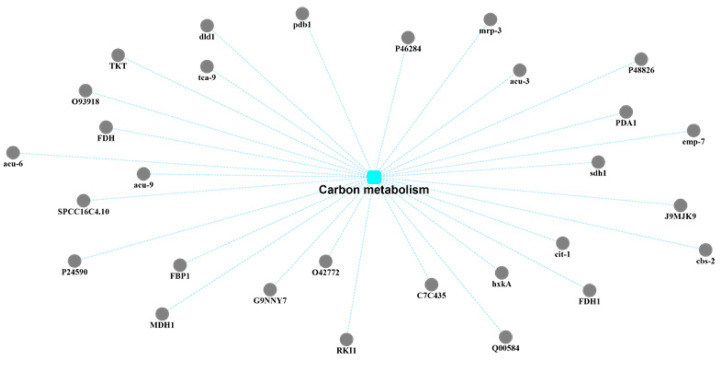
Protein–protein interaction (PPI) network analysis of the identified exudate proteins from *U. virens* that were related to carbon metabolism.

**Figure 6:**
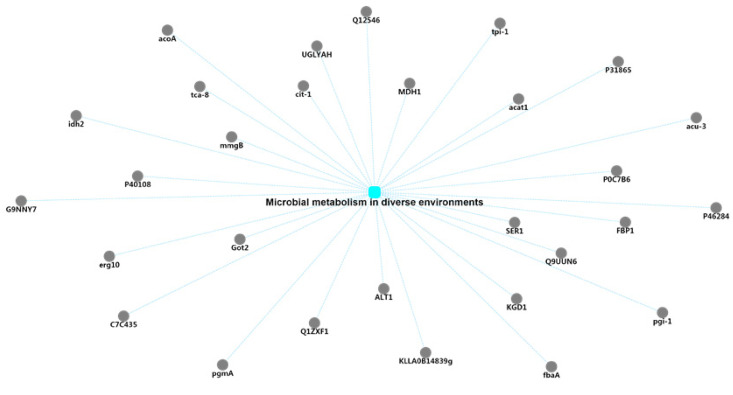
Protein–protein interaction (PPI) network analysis of the identified exudate proteins from *U. virens* that were related to microbial metabolism in diverse environments.

The exudate of *U. virens* performs the function of the secondary metabolism because of its various components. Therefore, the biosynthesis of secondary metabolites was the preferred target for the protein–protein interaction network analysis of the exudate proteins (Figure 4). Through correlation analysis, it was found that there were 20 proteins involved in this pathway. In addition, these 20 protein-related annotation pathways also included the FoxO signaling pathway, peroxisomes, phenylpropanoid biosynthesis, starch and sucrose metabolism, tryptophan metabolism, cyanoamino acid metabolism, phenylalanine metabolism, carbon metabolism, and glyoxylate and dicarboxylate metabolism. Among these 20 proteins, 15 of them were also linked to the pathway of carbon metabolism. Carbon metabolism is regarded as one of the most important metabolic pathways of the exudates from *U. virens* (Figure 5). Many proteins participated in this group and possessed their own functions, including gluconeogenesis, the glyoxylate cycle, the formate catabolic process, the succinyl–CoA metabolic process, the glucose metabolic process, the D-xylose fermentation process, ethanol oxidation, cellular glucose homeostasis, the D-ribose metabolic process, and the fructose metabolic process. Generally, the secondary metabolites endowed pathogens with different executive functions in different environments. Therefore, many proteins were annotated in the pathway of microbial metabolism in diverse environments through Protein–Protein Interaction (PPI) network analysis (Figure 6).

## 3. Discussion

By comparing and annotating the 650 identified proteins, it can be predicted that the exudate may be involved in the entire process of growth and development of the pathogen; this is consistent with research on *Cercospora armoraciae* [18]. Under artificial culture conditions, the exudates of *U. virens* not only appeared on yellowish colonies, but also on white colonies, which confirmed this hypothesis. In many fungi, exudates begin to appear from the early stage of pathogen development and gradually disappear until pathogen maturation [16,17], which was also observed in this pathogen. This phenomenon might have been caused by the limited growth space of the culture medium, resulting in the exudates being absorbed back into the pathogens to be used again [27], or due to evaporation [19]. In natural environments, no fungal exudation has been observed, which may be due to evaporation.

From the identified proteins, it was also found that the exudates played a very important role in the entire life of the pathogen. According to the identified proteins, it could be speculated that the exudates were involved in the formation of fungal cell walls, the growth of hyphae, and the formation of spores. Some of these proteins participated in the process of cell wall organization, including glucanases (KDB12613.1 and KDB14267.1) [31,32], glycosidases (KDB12546.1, KDB15368.1, and KDB13423.1) [33,34], mannoprotein (KDB10968.1) [35,36,37], and clock-controlled protein 6 (KDB12449.1) [38]. Glucans is an important component of the fungal cell wall, and is highly dynamic during cell wall morphogenesis. Glucanase activity, which involves the activity of hydrolytic enzymes, might contribute to remodeling of the fungal cell wall [32]. Glycosidases that belong to glycosyl hydrolases can participate in cell wall modulation [39]. The mannoproteins have an important role in controlling the porosity of the cell wall [40]. In addition, mannoproteins can induce host immune response [41]. Therefore, it could be inferred that the exudate is involved in the host’s immune response. Different groups of α-mannosidases have already been characterized in filamentous fungi [42]. Mannosidases might be involved in the degradation of the amorphous fraction and in the deglycosylation of glycoproteins of the fungal cell wall [43]. Since the exudate of *U. virens* was found to contain α-mannosidase (KDB13380.1), it could be speculated that the exudate might play a role in the formation of the fungal cell wall. Chitosanases have been found in abundance in a variety of species, particularly in microorganisms [44,45]. In nature, the chitosanases produced by microorganisms probably have the primary role of degrading chitosan from fungi for carbon metabolism, as well as for the temporal modification of the cell wall structures of filamentous fungi [44]. In this study, chitosanase (KDB15085.1) was also identified in the exudate; thus, it could be speculated that exudates play a role in modifying cell wall structures and carbon metabolism. Glucan endo-1,3-beta-glucosidase eglC (KDB14252.1), which was classified in the glycoside hydrolase family 17, is probably involved in cell wall organization. An eglC-null mutant isolate altered the hyphal cell wall structure in comparison with the wild type and generated a smaller number of protoplasts after digestion of the hyphal cell walls with a cell-wall-lyzing enzyme [46]. Beta-1,3-glucanosyltransferases (KDB18188.1 and KDB11503.1) were also identified in the exudates of *U. virens*. In fungi, beta-1,3-glucanosyltransferases play an active role in cell wall biosynthesis [47]. In addition to being related to the biosynthesis of fungal cell walls, they are also required for virulence [48,49].

Exudates might cause plant diseases [16,17,18] or predispose host tissues to further attack by the pathogen [22]. Superoxide dismutases (SODs) are well known for their evasion of oxidative bursts of host defense systems by catalyzing the disproportionation of superoxide radicals into molecular oxygen and hydrogen peroxide [50,51]. Cell surface Cu-only superoxide dismutase (KDB18714.1) was found to be similar to SOD5 in its sequence, and is a new class of SODs found in fungi [50]. This protein was found through the GO analysis to participate in the biological process of pathogenesis. Additionally, superoxide dismutase (Cu-Zn) (KDB18140.1) destroyed the radicals that are normally produced within cells and that are toxic to biological systems. This kind of protein was capable of maintaining cell redox homeostasis. Cooke [26] indicated that the exudates of *S*. *sclerotiorum* and *S*. *trifoliorum* maintained the internal physiological balance during growth. Therefore, it was speculated that the exudate of *U. virens* could also maintain cell homeostasis during the growth of the pathogen. As an important component of plant cell walls, cellulose can maintain the morphology of plant cells and act as the first barrier against pathogen infection [52]. Of the identified proteins, acetylxylan esterase (KDB11955.1), endo-1,3(4)-beta-glucanase (KDB13443.1) [53], cellobiose dehydrogenase (KDB12033.1) [54], and probably beta-glucosidase A (KDB16468.1) were involved in the cellulose catabolic process, which might endow the exudates with a phytopathogenic ability.

In addition to the role of exudates in pathogenicity and cell wall formation and remodeling, they also have some other important functions. The cell wall protein phiA (KDB17876.1) is involved in the development of asexual structures and is required for spore formation. When the *phiA* gene was disrupted, the *phiA* mutation resulted in reduced growth and severely reduced sporulation of *Aspergillus nidulans* [55]. It was speculated that the exudate might be involved in the pathogen’s spore formation. The exudate also had antioxidant activity, which was confirmed in many pathogens [19,20]. Disulfide-bond oxidoreductase (KDB15731.1), catalase (KDB11441.1), and catalase–peroxidase (KDB15626.1) [56], which were identified in the exudates, possess the ability to respond to oxidative stress. In *Sclerotium rolfsii*, the exudates inhibited fungal morphogenesis, including spore germination, because of its phenolic components [19]. Phenolic acids play an important role in the antimicrobial processes of fungi [57,58]. It was speculated that the exudates could protect the pathogen from being attacked by other microorganisms.

## 4. Materials and Methods

### 4.1. Fungal and Exudate Materials

The three representative *U. virens* strains (SyUv-01, DdUv-05, and PjUv-07) used in this study were isolated from infected *Oryza sativa* in Shenyang, Dandong, and Panjin in the Liaoning Province of China, respectively. The isolates were placed in potato sucrose agar (PSA) medium at 25 ± 1 °C. After 5 days of cultivation, the mycelium on the outer edge of the colony was picked up with an inoculation needle and placed in a new PSA medium. After the isolates were cultured on the new PSA medium for 25 days, the exudates were collected from the mycelium. The exudate samples were stored in a 1.5 mL tube (Sangon Biotech, Shanghai, China) and quickly passed through liquid nitrogen, then stored at −80 ℃.

### 4.2. Enzymatic Hydrolysis of Proteins

The protein concentration was measured by using the bicinchoninic acid (BCA) method. Proteins (200 µg) were added to 1 M DTT (final concentration of DTT: 100 mM; the sample reconstitution volume was 50 μL) and then placed in a boiling water bath for 5 min. The filter-aided sample preparation (FASP) method was used for proteolysis. The sample was added to 200 μL UA buffer (8 M urea, 150 mM Tris–HCI, pH 8.0) and the well-mixed protein solution was transferred to an ultrafiltration centrifuge tube. The homogenate was centrifuged at 12,000× *g* for 15 min. and the filtrate was discarded. IAA (50 mM iodoacetamide in UA) was added to the ultrafiltration tube. It was incubated in the dark at room temperature for 30 min and centrifuged at 12,000× *g* for 10 min. The filtrate was discarded. UA buffer (100 μL) was added into the ultrafiltration tube and centrifuged at 12,000× *g* for 10 min. This process was repeated twice. NH_4_HCO_3_ buffer solution (100 μL) was added into the ultrafiltration tube and was centrifuged at 14,000× *g* for 10 min. This process was repeated twice. Finally, the proteins were digested with 40 μL trypsin buffer (4 μg trypsin in 40 μL NH_4_HCO_3_ buffer) and incubated at 37 °C for 16–18 h. The dried peptide was dissolved with 0.1% trifluoroacetic acid (TFA) for liquid chromatography–tandem mass spectrometry (LC-MS/MS) analysis.

### 4.3. LC-MS/MS Analysis

The peptides were separated by a Q-Exactive Plus mass spectrometer that was coupled to an Easy nano liquid chromatography instrument (Thermo Scientific). The samples were first loaded onto a trap column (100 μm × 20 mm, 5 μm, C18, Dr. Maisch GmbH, Ammerbuch, Germany) and passed through a chromatographic analysis column (75 µm × 150 mm, 3 µm, C18, Dr. Maisch GmbH, Ammerbuch, Germany) for gradient separation at a flow rate of 300 nL/min. The mobile phase consisted of solvent A (0.1% (*v*/*v*) formic acid in H_2_O) and solvent B (0.1% (*v*/*v*) formic acid in acetonitrile). The gradient was set as follows: 0–2 min, gradient of B liquid was from 3% to 8%; 2–170 min, gradient of B liquid was from 8% to 26%; 170–206 min, gradient of B liquid was from 26% to 40%; 206–216 min, gradient of B liquid was from 40% to 100%; 216–240 min, the gradient of liquid B was maintained at 100%. The peptides were separated with a Q-Exactive Plus mass spectrometer (Thermo Scientific) for data-dependent acquisition (DDA) mass spectrometry (MS) analysis. MS data were acquired using a data-dependent top-20 method that dynamically chooses the most abundant precursor ions from the survey scan (350–1800 *m*/*z*) for higher-energy collision-induced dissociation (HCD) fragmentation. The full MS scans were acquired at resolutions of 70,000 at 200 *m*/*z* and 17,500 at 200 *m*/*z* for the tandem mass spectrometry (MS/MS) scan. The maximum injection times for MS and MS/MS were both set to 50 ms. The normalized collision energy was 27 and the isolation window was set to 2.0 Th.

### 4.4. Sequence Database Searching

The MS data were analyzed using the MaxQuant software version 1.6.1.0. The MS data were searched against the UniProtKB *U. virens* database. Trypsin was selected as a digestion enzyme. A maximum of two missed cleavage sites and the mass tolerances of 4.5 ppm for precursor ions and 20 ppm for fragment ions were defined for the database search. The carbamidomethylation of cysteines was defined as a fixed modification, while the acetylation of protein N-terminals and oxidation of methionine were set as variable modifications for the database search. The database search results were filtered and exported with a <1% false discovery rate (FDR) at the peptide-spectrum-matched level and protein level, respectively.

### 4.5. Bioinformatic Analysis

To annotate the sequences, information was extracted using UniProtKB/Swiss-Prot, Kyoto Encyclopedia of Genes and Genomes (KEGG), and Gene Ontology (GO). GO and KEGG enrichment analyses were carried out with Fisher’s exact test and FDR correction for multiple tests was also performed. The GO terms were grouped into three categories: biological processes (BP), molecular functions (MF), and cellular components (CC). The construction of protein–protein interaction (PPI) networks was also achieved by using the STRING database with the Cytoscape software.

## Figures and Tables

**Table 1:** List of proteins identified with liquid chromatography–tandem mass spectrometry (LC-MS/MS) in the exudates of *Ustilaginoidea virens*.

NO ^a^	Protein Functions ^b^	Protein IDs ^c^
1	fungal-type cell wall organization	KDB16044.1, KDB16026.1, KDB12967.1, KDB17270.1, KDB13423.1, KDB12509.1, KDB10968.1, KDB12449.1
2	pathogenesis	KDB16026.1, KDB18474.1, KDB18473.1, KDB13423.1, KDB13264.1, KDB17801.1, KDB14282.1, KDB12970.1, KDB12705.1, KDB12369.1, KDB12341.1, KDB12056.1, KDB12863.1, KDB14729.1, KDB12463.1, KDB18714.1, KDB11931.1, KDB11462.1
3	response to oxidative stress	KDB14292.1, KDB12590.1, KDB18386.1, KDB17179.1, KDB13850.1, KDB15471.1, KDB13484.1, KDB11596.1, KDB11345.1, KDB11441.1, KDB15626.1, KDB16892.1, KDB13523.1, KDB15731.1, KDB18611.1, KDB16877.1, KDB17534.1
4	sporulation resulting in formation of a cellular spore	KDB13075.1, KDB17876.1
5	ascospore formation	KDB12701.1, KDB11591.1
6	spore germination	KDB17080.1
7	cell wall organization	KDB16967.1, KDB18910.1, KDB15368.1, KDB12546.1, KDB14267.1, KDB19043.1, KDB12613.1, KDB14252.1, KDB13987.1, KDB18188.1, KDB11503.1, KDB16319.1
8	cellular response to osmotic stress	KDB18447.1, KDB11395.1, KDB13915.1, KDB18874.1, KDB17080.1
9	carbohydrate metabolic process	KDB14278.1, KDB18474.1, KDB18473.1, KDB18838.1, KDB14575.1, KDB11829.1, KDB14601.1, KDB14600.1, KDB15368.1, KDB12546.1, KDB11271.1, KDB14684.1, KDB12548.1, KDB15611.1, KDB10889.1, KDB14267.1, KDB14320.1, KDB12419.1, KDB18213.1, KDB13612.1, KDB15373.1, KDB13423.1, KDB11780.1
10	cell redox homeostasis	KDB16889.1, KDB12590.1, KDB18386.1, KDB17179.1, KDB18140.1
11	cellulose catabolic process	KDB12033.1, KDB16140.1, KDB12252.1, KDB16790.1, KDB11955.1, KDB16468.1, KDB13443.1
12	chitin catabolic process	KDB15606.1, KDB11464.1, KDB12586.1
13	oxidation–reduction process	KDB16889.1, KDB18474.1, KDB18473.1, KDB17420.1, KDB12801.1, KDB15461.1, KDB12059.1
14	filamentous growth	KDB16843.1
15	tricarboxylic acid cycle	KDB12786.1, KDB14278.1, KDB14601.1, KDB14600.1, KDB10889.1, KDB13150.1, KDB16991.1, KDB17192.1, KDB16006.1, KDB11543.1, KDB10744.1, KDB14658.1, KDB17818.1, KDB11496.1
16	translation	KDB17180.1, KDB17178.1, KDB13202.1, KDB17437.1, KDB17624.1, KDB16501.1, KDB12514.1, KDB15720.1, KDB18497.1, KDB13460.1, KDB12690.1, KDB11557.1, KDB18340.1, KDB13903.1, KDB18121.1, KDB17697.1, KDB12335.1, KDB16500.1, KDB11526.1, KDB11525.1, KDB11907.1, KDB15905.1, KDB16255.1, KDB15436.1, KDB14900.1, KDB13937.1, KDB13450.1, KDB11439.1
17	amino acid metabolism process	KDB12840.1, KDB18587.1, KDB14255.1, KDB18363.1, KDB16051.1, KDB18484.1, KDB12951.1, KDB13778.1, KDB13915.1, KDB13888.1, KDB14012.1, KDB14608.1, KDB13578.1, KDB16028.1, KDB11789.1, KDB11862.1, KDB16889.1, KDB16190.1, KDB11291.1, KDB12646.1, KDB11239.1, KDB11321.1, KDB12537.1, KDB18031.1, KDB11222.1, KDB17215.1, KDB15208.1, KDB16374.1, KDB18200.1, KDB16669.1, KDB18959.1, KDB10792.1, KDB18706.1, KDB18773.1, KDB11366.1, KDB11543.1, KDB15471.1, KDB15846.1, KDB12825.1, KDB11677.1, KDB18285.1, KDB12314.1, KDB17813.1, KDB15640.1, KDB16843.1, KDB12482.1, KDB17017.1, KDB11426.1, KDB14889.1, KDB11692.1, KDB13308.1, KDB15264.1, KDB18161.1, KDB15909.1, KDB13150.1, KDB12414.1, KDB13272.1, KDB12151.1
18	fatty acid biosynthetic process	KDB13931.1, KDB13929.1, KDB16107.1
19	glycolytic process	KDB18218.1, KDB19042.1, KDB15252.1, KDB11157.1, KDB14945.1, KDB14792.1, KDB11877.1, KDB13798.1, KDB18458.1, KDB16050.1, KDB15065.1, KDB10987.1
20	defense response to fungus	KDB14422.1, KDB11072.1
21	mannose metabolism process	KDB12509.1, KDB13367.1, KDB16099.1, KDB15243.1, KDB13380.1
22	response to toxic substance	KDB11815.1
23	protein folding	KDB18447.1, KDB13877.1, KDB14801.1, KDB15561.1, KDB18092.1, KDB15388.1, KDB17676.1, KDB15520.1, KDB13738.1, KDB11515.1, KDB17022.1, KDB12004.1, KDB11307.1, KDB11203.1, KDB13922.1
24	antibiotic biosynthetic process	KDB15025.1, KDB13507.1, KDB14422.1
25	fungal-type cell wall polysaccharide metabolic process	KDB18520.1

^a^ Protein function number. ^b^ The protein functions obtained from UniProt database. ^c^ Proteins involved in the function.

## Data Availability

There were 650 proteins identified in the exudate; the raw data are available via ProteomeXchange with the identifier PXD019861 (http://proteomecentral.proteomexchange.org).

## Supplementary Materials

The following are available online at https://www.mdpi.com/2076-0817/10/3/364/s1: Table S1: List of proteins identified with LC-MS/MS in the exudates of *Ustilaginoidea virens*.
