# Supplementary material for: Proteomic Analysis of Mycelial Exudates of Ustilaginoidea virens
Source: Pathogens. 2021 Mar 18;10(3):364. doi: 10.3390/pathogens10030364 (PMC8003167; doi:10.3390/pathogens10030364)
Supplement: Supplementary file 1 [file pathogens-10-00364-s001.pdf]

Supplementary Table S1 List of proteins identified with LC-MS/MS in the exudates of *Ustilaginoidea virens*

NO [a]	Protein IDs [b]	Protein names [c]	Score [d]	Mol. weight [kDa] [e]	Cov [f]	MS/MS Peptide sequence [g]
1	KDB17818.1	Succinate dehydrogenase [ubiquinone] iron-sulfur subunit, mitochondrial	6.282	30.486	4.1	TGPMILDALVR
2	KDB18023.1	3-ketoacyl-CoA thiolase, peroxisomal	6.2998	43.626	2.1	ALDLAGISR
3	KDB12646.1	ATP phosphoribosyltransferase	25.709	34.047	17.6	AIDTVVQSTAVLVQSR EIALVMDELSR SSTNTDMVDLIASR VGASDILVLDIHNTR
4	KDB11684.1	Bifunctional purine biosynthetic protein ADE1	22.54	86.534	4.5	GLAHITGGGLIENVPR SLLPVLGEIK TVGESLLTPTR
5	KDB16707.1	Proteasomal ubiquitin receptor ADRM1	12.204	42.367	4.3	GSGSGGAGPDATGGDVR
6	KDB15928.1	Cytochrome b2, mitochondrial	34.9	58.379	9.4	EFDPVHPSDTLR GVQTVEDVLR MLTGADVAQHSDAK SGIEVLAETMPVLR
7	KDB12275.1	Aspartate 1-decarboxylase	11.724	112.62	3.6	GLILTLSEIPEASK TAAIAGLGSGNIIGIPVDNAAR
8	KDB15972.1	Glucosidase 2 subunit beta	7.3902	64.984	3.2	IDPLSPQQLLPASGLAPGR
9	KDB15004.1	Ribose-5-phosphate isomerase	70.089	32.491	32.6	AAGLALGALDDRPLDGR AIPIEVLPLAAPDVLAR AVDDHLLPSYR GGGACLLQEK GPAFHAR KLIAVADSR LIAVADSR MTFFPTGSQSK YVGIGSGSTVVHVVDAIASK
10	KDB18474.1	D-arabinitol dehydrogenase 1	19.425	25.025	19.2	ENPEAQFDQLKK ILEDAIHYVR NLNWVDATLLEPASCACHGLEK
11	KDB18473.1	D-arabinitol dehydrogenase 1	11.481	10.294	36.6	FPLIPGHETVGVIAAVGK VAADNSELCNECFYCR
12	KDB15780.1	Cyanovirin-N homolog	85.42	11.188	31.7	QVINLDER TASNVQLQGSQLTAELATLSGEPR
13	KDB14501.1	14-3-3 protein homolog	71.578	34.763	34.4	GAATAAHEAYK IELELEK KEEGDSTEKPAEETK LGGELTVDER NATDVAQTDLTPTHPIR QAFDDAIAELDSLSEESYR YLAEFASGEK DSTLIMQLLR
14	KDB13397.1	14-3-3 protein homolog	120	29.882	55.8	AATEVAQTELPPTHPIR DLTVEER DSTLIMQLLR EETSAAAEPAVASEEAK GHEDAVYLAK GNFSQVTLIK HLIPSAK ICEDILDVLDK IEAELAK IVASEDRDLTVEER NLLSVAYK QAFDDAIAELDTLSEESYK YEEMVENMK YLAEFAIGDR
15	KDB13503.1	Glucan endo-1,3-beta-glucosidase BGN13.1	275.8	80.016	48.5	AGDVGTAQIQDMR AGGFWYADMDHTGAPR AVKPGHGADIQDAIDSAVNGPR DPMQNGGHTIK DTNSDIVQLPSGTALGAASHVNNFSFGNTVGR DTSVALR GAPDLTGSCTDASNECMGAFLGLHFAK GDGTVDEAAALNDVLQFAAQHNK GGALVESTK GSTLGLADVR GYAPHADK HPIYGATQSSATR IAYFPFGDYR IVGEAWSTISGAGQFFK NGQGHTGIELGR NLQAYGLCAK NTGGSPFVAIIDAR PAAAAPGGR PGDVALWNSLITVGGTR PGHGADIQDAIDSAVNGPR PVVQVGK SASNVVVTLLQSETNYEQGDNAR SINSGVTFDSSVYPSIVIDNLTK VESTLLIPIGSR VVYIPSGTYTLSK YMHYISQTPK
16	KDB15456.1	Beta-mannosidase B	127.9	96.73	23.6	DDSAYLPVAQFPTQIHLDLLHHK EMAPISIGIK FDDNLIDIVPGETVAIGVR FVSEFGMEAFPSIK GLAVECEDDKVR HVNWPEPLK PVNLELYESR QTYSDTVSAK RVELVQQPLIDQPGTSFFFK SVASETVSVIDGQAVATFK TILETDNMFVSER TTFSTPEIGGR

17	KDB18838.1	Phosphorylated carbohydrates phosphatase TM_1254	79.93	27.379	47.2	TVVAAYLHQDGK VELVQQPLIDQPGTSFFFK VHVTTETK VQNPELWYPIR ASLRPCPGVDEELEK DWSEFPAALQK EAGESVAIEDSK IESAQSEAR MEEDAVIAK SPGGEYLIYSAATSLNPPTSK TFTGESLIVEFVGQNFR TIGYVGPYADDK TIGYVGPYADDKK VLTDAGAVVIMR
18	KDB11613.1; KDB17942.1	Histone H4.1	12.245	10.759	18.9	DAVTYTEHAK TFLEGVIR
19	KDB15025.1	O-methyltransferase MdmC	11.01	29.905	11.3	LLRPGGLIVADNVLR LRPSAPYDIVFLDADK
20	KDB13507.1	Phenylpyruvate C(3)-methyltransferase	129.35	38.767	36.6	EINHVYYDPAFWK FLLGDAHR GSVGADSDSQR HFEEAYK LLLNSR LMEILGR LSFVEGNVLALEYR QHDLNPVSTEAIVAALMAVDILAK RFLLGDAHR SIFHFTSLGSGGLFAK SVVDLGCGSGER YDGEVAGGK
21	KDB11815.1	Probable nitronate monooxygenase	6.1623	40.434	2.9	GTIPYEVDLER
22	KDB15836.1	Short-chain dehydrogenase/reductase SAT3	45.035	31.082	29.8	AGALDGDLVPLTR AITGPLDHFADIPSLQQK AVYILGR EESLLSAK REESLLSAK VGSEEDYAGVVLFLTSK VNAIAPGFFPSEMTQNLPFMNSDK
23	KDB15845.1; KDB15848.1	Peptidase M20 domain-containing protein SMAC_03666.2	126.93	38.373	45.8	DLISIPSITGDENNVGR FDGGVAANVIPETAK FNILAWK GAPGHSGYPWLGK GGLFCDVVAK IATGPEESAHLVVK ILDTDLGSSELFGDTTFNIGR NPLRPEAVIFGEPTENR SANELMVR SGYNAQLQSVAEEGSPK VQSILDEVDK VQSILDEVDKQAFEMICTHGYGSVK
24	KDB19047.1	Putative calmodulin-3 (Fragment)	49.577	8.5193	69.9	DNNGFISAAELR DTDSEEEIR IDYNEFVQLMMQK LTDDEVDEMIR MKDTDSEEEIR VFDRDNNGFISAAELR
25	KDB19043.1	Putative metallocarboxypeptidase ecm14	7.8732	61.828	2.7	EYIVPTGEEVFSALK
26	KDB19153.1	Hydroxymethylglutaryl-CoA synthase	23.736	52.204	10.1	LLYHDYLSNADAPAFAEVAPELR PTGGAGAVAMLIGPDAPVVMEPGLR
27	KDB19133.1	UPF0587 protein C2D10.03c	14.616	18.282	20.6	FEANEMSGSR GLEFTEFRPEGEWLAEGIDSGTK
28	KDB19078.1	Uncharacterized aminotransferase C6B12.04c	13.524	12.675	21.1	DDEVLEEAK LPADYPFPEHVASR
29	KDB19022.1	40S ribosomal protein S3	41.511	28.189	23.7	AAAAQASQDAR ATQTQEVLGEQGR FTDGFMIHSGQPAK GLSAVAQCESLR VTPTVTDIIIR
30	KDB18959.1	Homoisocitrate dehydrogenase	6.6339	37.885	4.8	SAALMLEFLHEPAAAAK
31	KDB18916.1	Synaptobrevin homolog ykt6	19.814	22.545	19.8	NESKPAHELVAEK QDVEEQDYTFHAYGR VVDEFLSNHPR
32	KDB18803.1	Probable mannosyl-oligosaccharide glucosidase	19.396	90.818	5.2	EQILGDEAR ILDVMGDEEQLWSAHGIR TAHLDNPELGEDFLR
33	KDB18592.1	Protein vip1	18.288	28.693	12.2	ANSFLTGISSYFEK ATDQSYGISQR VETEGETK
34	KDB18520.1	Alkali-sensitive linkage protein 1	70.897	38.349	32.6	ALFSFNEPDNR ALIGAPAVSNSNLAGEGLDWLK APSSPAAASPAK EVSNWDSAATSAIANGAK GQADMTPSVAADAHVQYMNK GSSAGSSASSGGPGFSGK NCGWAYSWGK YPGSLDSK
35	KDB18324.1	Guanylate kinase	15.594	24.11	11.2	GTEDEASIR YVFIAPPSLDALEAR
36	KDB18218.1	Pyruvate dehydrogenase E1 component subunit	21.025	41.506	8.3	DLTIVSLSR NYGIEAEVINLR VVSPWSAEDAK

		beta, mitochondrial				
37	KDB17808.1	UBA domain-containing protein 7	6.171	99.842	1.1	PTPSPAGSGR
38	KDB17676.1	Probable T-complex protein 1 subunit beta	16.962	57.215	5.5	GATEQLLDEAER SLHDALAVLSQTVLEPR
39	KDB17613.1	Probable proteasome subunit alpha type-3	22.068	27.896	13.5	DGIVLAAER IEFATVGQTEEGK LWSADEITALLK
40	KDB17471.1	Curved DNA-binding protein	13.14	45.258	6	ILEQPLSR SADLLLATHYANELLLR
41	KDB17446.1	Adenosine kinase	43.092	36.451	14.6	ANDAILAEEK DLATSVDMGQWLAR HLPLFEDLLNNFDAK KPEIWSLVEK
42	KDB17323.1	Deubiquitination-protection protein dph1	15.912	45.575	5.8	LAGEEFENIPIER QLNDMGFYDFDR
43	KDB17241.1	Protein pim1	6.2123	57.53	1.1	LDQIAR
44	KDB17215.1	Probable aspartate-semialdehyde dehydrogenase	38.371	46.159	14.7	AEMPVFSNAK DLGNGYTVSVGR LRQDESGVFDMK QSAPMGGATAEMVVR VPVLDGHTACVSLR
45	KDB17084.1	Probable proteasome subunit alpha type-5	7.3266	25.811	8.1	AIGSGSEGAQAELQNEYHK
46	KDB16991.1	Fumarate reductase	38.454	66.932	9.9	AQVLNQDK DATEEFEMLHDDEVIPK EGDLWVVVK FLAAEALR VTGVTYELDGK YAPNQVIGR
47	KDB16892.1	Uncharacterized RNA-binding protein C328.05	52.831	52.428	10.2	FDTADTAETAITK FQGYQYGGR PLNLSFVK SETIYVR VEQAEIQYEPSGR
48	KDB16889.1; KDB16893.1; KDB16891.1	Dihydrolipoyl dehydrogenase, mitochondrial	180.09	147.9	16.8	AEEEAVAVVEYIK EAAMATSSK ETAVNGLTK GAGSFINENDIK GGKPETLDAEVVLVAIGR GIEVGDVK GTLGGTCLNVGCIPSK LNLANFMK MLADPETDR NILIATGSEATPFPGLEVDEK NILIATGSEATPFPGLEVDEKR RPYTQGLGLENIGMELDDR SLLNNSHLYHQILHDTK TCHAHPTLAEAFK TNLDTDGLVK VALNDGGETTVR VIIDSEYR VTVVEFLGQIGGPGMDTEIAK
49	KDB16860.1	Probable hydroxyacylglutathione hydrolase C824.07	92.923	31.565	56.1	ATGATDPVDVMAK AVFTGDTLFVSGCGK DAVIIDPANPPEVAPILK FAISVLQSEPVK KLEEFAASNQVTTGEFTLADEK LDDPAVQK LGSLPDDTVVYPGHEYTK LLAELGTPGLAIVGGK SKDAVIIDPANPPEVAPILK TPAHEEVFQLGDIK VTSVHTPCHTQDSICFFMEDGTGR
50	KDB16819.1	Septin homolog spn4	131.95	57.127	21.4	ATATAAPAPSR DSVVALLQDWPANAPSFLGR GDHYPDFLSHFQR GWQAVVEEHLCR TAELLHLNAYVAAAAAAGHPPHLPK TYQLAAK VQQTEEAASK VSELLQEDLQK
51	KDB16630.1	60S ribosomal protein L4-A	60.418	37.983	37.2	ALDDIYGSTTEASSHK AVGAGPDLDK DFFLPSNVVSQADLTR GHEIMSVAEVPLVVDSTLFEGAAITR LINSSEIQSSLNAPK NIPGVETSPVSALNLLQLAPGGHLGR QPYAVSEK RGPLVVYNPEVDGK
52	KDB16535.1	Importin subunit beta-3	25.394	105.24	2.5	ALLALIDLAETAPK ISDAVAEVAR
53	KDB16489.1	Ran-specific GTPase-activating protein 1	24.174	27.481	16.5	AEAGEDEAPESEDVHFEPVIR ANEATEATEAK LSPNVGSDR
54	KDB16319.1	Glutathione S-transferase omega-like 2	58.006	40.915	25.9	DENEPGENVTPDPNEGHEDFTHLR DWISSEAGAK FTVPVLYDK MLGTEFDGFLDETHR NVTALFEALDR SGFATTSAAYER SIQLYPEPLR
55	KDB16121.1	Diphosphomevalonate decarboxylase	22.819	41.692	16.9	AVADLYELPDSPSELSLVAR GVSSTSGMQQTVATSGLFQHR LEDGIASTLR

						QIVPSNMDMMEEAIR
56	KDB16099.1	Meiotically up-regulated gene 157 protein	97.844	58.417	32.9	ACAEIMQPIDENTASR AVELYGVVK DANSATETVSNSGSGNPVLGNIGLVR DFVLSR FPYCNAFQPPPEAK GTINLQAR GTYADDGHVNTSPYTWLR HHWADAVR HPPYSPGR MAEGIER QLMGATSGLGLIHESVNTWNDQK SYVVPDVDK TILSLADDMMR YPSQRPEVK
57	KDB16013.1	Probable 5-hydroxyisourate hydrolase	15.849	14.115	17.2	TFESLTDDDGR TFFPEAVVVFR
58	KDB16010.1	Cys-Gly metallodipeptidase dug1	204.35	52.627	50.4	AVAIPSISAEDAR EGGSIPVTLTFEEATGK EHLQLPPVVLGR ESTLMAR FPSLSIHGVEGAFGGSGAK GATDDKGPVLGWLNAIEAHQK GPVLGWLNAIEAHQK HWNFSAAAK IQIPGIMEQVVPVTEEER LLGAYLHYVAEEPQS LLGSLVDTHGK NVLLLPMGSSTDGAHSVNEK QVDDSANAFIER RPDVVR SLYDGISFTMEDLHESLGSK TILVYGHYDVQPAEK TTIYEDK VFAQHTGK VWGVQPDFTR
59	KDB15864.1	60S ribosomal protein L14	8.309	25.882	6.9	VLVDGPSADPALAVPR
60	KDB15614.1	Uncharacterized methyltransferase C1B3.06c	74.182	30.166	22.6	AQIEDASGAWR EEIDWWSGLWSER LVPQGHVTGLER PGGIVAAR TMASGFAESAIETGTATR
61	KDB15561.1	10 kDa heat shock protein, mitochondrial	50.015	21.299	32.8	AGEEEYQLFR LPMGVAVGDR SLAPLLDR TAGGIFLPESSVEK VLAVGPGALDK VLIPSFGGSPVK
62	KDB15520.1	Heat shock protein sti1 homolog	25.474	63.824	10.1	ALGTENYK DTPMTDAHAPESK FTQAIALQPENHILYSNR KAPEPEPEPEPMDEEALEK
63	KDB15471.1	Probable peptide methionine sulfoxide reductase	55.523	26.235	33.6	ALLEFFYR GGIVTEIAPAGR MHDPTTR NAQGPDAGTQYR SAIFFHDGEQER TGHAEAAQIIFDPR WWTAEEYHQEYLGR
64	KDB15388.1	Uncharacterized protein C1711.08	47.536	36.392	29.2	FELFGGNVSGEYLELEPGKK LAPALIEEHGK LQIEFDQNDVDHVTVMR TTADELYQTFVDPQR TVTDNEEFR VITIFDVK VTWDGVPVGQEEVTK
65	KDB15344.1	Uncharacterized protein C9E9.15	56.428	29.717	24.5	DELPPPR ELNLASVK FHGHLDTTALGGAGFASQHSR GGASTSHLTVL$GPR GPEPADGK GPEPADGKR YALTLK YALTLKDELPPPR
66	KDB15262.1	Probable glutamine--tRNA ligase	6.644	71.808	1.9	TVGLLNAPYPVK
67	KDB15243.1	Meiotically up-regulated gene 157 protein	11.581	56.418	5.6	LFENAFPSTTDTTVR MILDSSGNPYYLR
68	KDB15208.1	Branched-chain-amino-acid aminotransferase, mitochondrial	129.49	45.201	40	DINIPMGPQGQPGPVTAK EAAAPHVPSAPGAVPAPLDASR EQHHWGVVIPEK GDGRPELVTAPLDDK GWLSDIMYGR ISLPLFDPPELQR KYTIDELLEADAEGR LHTSPEDMVR LILDGVTR LTYALTASPR MAGELQVTER MLLSAGR VGANYGPSLMATQDAR YTIDELLEADAEGR
69	KDB15161.1	Uncharacterized protein C3H1.02c	135.98	118.51	19.7	ALADIPEEK DGNRVDFEQVVMELER DSIGYGIGSSR DVTADEIR EELASSITNWWEAR FLVTADVAK FVDLLNDEGR IATGEVAIDK NDQPIPAEIIDR NFIQNVVR NIDSGILSYR PNSVIWLQPTGVATDK QELGTAGLEK SIGLGTGPAQTMIDSAR TMMFDSVFDPQR VDFEQVVMELER WSVPGTESIHFIESDTAR WTDEEWR YETGGMTDALR
70	KDB15156.1	Elongation factor 1-beta	26.595	26.08	26.4	HIASFEDEFASLSGDASK LIPVGFGIK PYTVYGPEASELTLNPAK SVVTLDVKPWDDETDMK

71	KDB15096.1	N-acetyltransferase ats1	6.2624	20.12	6.6	RTDAPAILELIR
72	KDB14889.1	Uncharacterized hydrolase C21C3.09c	82.114	26.291	33.7	ADDTQGALEAIK GFDTFLPMSNVIPK GLPWDIAK QDGSTGLMIYPIPR VMTLHPGDIVLTGTPAGVGPVVPGDVMR VVCIGR
73	KDB14806.1	Pseudouridine-metabolizing bifunctional protein C1861.05	13.452	86.478	2.7	EAASIVLAQER NLPLEHEELVR
74	KDB14765.1	Uncharacterized transporter C757.13	47.174	88.038	6.7	PFFDEVFAQTGCR SPGVLAGR TATLLAK TQITGWLAEDAPSFDYAGYVVGEGER
75	KDB14670.1	Protein NIF3 homolog	11.982	34.863	8	HLMVASPFGADVR SWDNVGLLVGNSK
76	KDB14575.1	Probable 6-phosphogluconolactonase	137.51	28.346	72.3	AIDAAHLDDTQELADQYEK AYIVQSQNAGFCR CSWFVDEPAVQGVAFPR ETDAWVAPIEDSPKPPPR PDDKVDFAK IFDSPEGLPCTLVNEAAGER ITLTLPVVTHAVR LPADQQPTVR TLAQALLAPPR TLVGSFASR TQPNLYAFPDVDALAPALR VAFVATGSGK VAVSGGSLPK WEIFFADER
77	KDB14491.1	SDO1-like protein C21C3.19	33.642	12.79	36.5	HGSIQSMEMPGR NILHAEFGTDNTDDVITR QGSQGTYDTAPK
78	KDB14144.1	Importin subunit beta-1	9.0168	99.573	1.8	VAANEVVPVLLSLLTK
79	KDB14085.1	Uncharacterized AIM2 family protein C30D10.14	44.574	48.034	19	DAEGVNVPTALLASGDEPAEDVKK DQIHGWMAAR FPPPDIAGK GSPAAIEWYPPDTAEK IPAYVQAVK SNLSDAR VLIPDWFK
80	KDB14047.1	Riboflavin synthase	9.4948	25.235	7.4	ITNLGTLVENSPVNLER
81	KDB13994.1	Protein-glutamate O-methyltransferase C1393.13	41.984	57.333	17.2	AFFETQSEDDRLQGK LPSEAPELHEDLK NAVVELAAR NILINDLPAAYDLLK TPAPLADKDADDLVFVFQDWAR WPVILTGALDDVHR
82	KDB13944.1	Hypoxanthine-guanine phosphoribosyltransferase	7.902	22.93	5.4	ILIVDEVDDTR
83	KDB13931.1	Probable ATP-citrate synthase subunit 2	66.306	53.407	23.5	AATQELGLNAK AGPNYQEGLK EEAYIAEMDAK QFLVEPFVPHSQDTEYYININSVR SGLLALNK SPAALGLTNIAPVNGEK TVLDLMLR VEHTTGVLR VNIDAGPPMEFPAPFGR
84	KDB13915.1	Protein arginine N-methyltransferase skb1	243.91	149.88	9.8	AALAECGYK AVADLGLK AVDVVQNR GIIFAGGLHK IITATHVDTSTGVLFDLK ITGAVGGR ITGAVGGRPQPDEIEQALR IVAERPEDQAHAMTAIYLPDSVSIPSILPK KAVADLGLK KTEPATYFASMK PQPDEIEQALR RAVDVVQNR RPDIDNALEALR TEPATYFASMK TLTDVVK
85	KDB13912.1	Exportin-1	18.698	122.4	3.9	AVTIEELDTTVR HFVALQPSEQEPFIEEIVR ILSSAQYPQTK
86	KDB13888.1	Probable 5-methyltetrahydropteroyltriglutamate--homocysteine methyltransferase	222.86	85.872	42	AGVDVIQVDEPALR AIETVETAIQK AVNDFNEAK AVTEGPASVR CVRPPIIVGDISR DDVHQSVQAQQLALALR DEVVDLEK DGLDAVDQYFAMGR DIYAVHVDLVR EAGAESVQIDEPTLVFDLPAK GEISEEQYDK GGIDVPSLEMVK GQLEANAK GQSVDPIDLLNK HIGPGVYDIHSPR ISQADLLAEAK KLDPEVADWFSFASEK LLPVYEQLLTK NPEQLETVAGALGSK PAPMTVK PVLVGPVSFLHLGK QSEFPTR TILSAGVVDGR VFVDSAYPR VIVATSSSLLHTPHTLASEK VPSEQEIR VQSAILGFPR

87	KDB13877.1	Protein wos2	14.92	22.245	14.9	AEVATPEVLWAQR YHVELPFYAEIDPADSK
88	KDB13858.1	Vesicle transport v-SNARE protein vti1	23.836	25.29	17.6	ALEEADELLDSMR LSGLASDR LVQADLLQK NYEADIDAHR
89	KDB13809.1	40S ribosomal protein S20	6.2157	13.017	10.3	LIDLHAPTEVVK
90	KDB13766.1	Protein phosphatase 2C homolog 2	43.17	47.424	14.4	ANSVAEAGQQDQNQGTK ISMEDAHTTVLDLASQGISLDAK LSSTDNGAVESK QDSEAQPATGK
91	KDB13738.1	T-complex protein 1 subunit delta	6.5395	56.92	2.4	GIHPSVISESFQR
92	KDB13719.1	Thioredoxin-like protein 1	44.887	12.944	49.6	AISPLFEK HSDALAADHAVAFAK PVITVTSK QQFDQLIR TAVDDLTK TSVVTGANPGSLK
93	KDB13528.1	Threonine--tRNA ligase, cytoplasmic	8.5102	84.744	1.6	NEFSGALSGLTR
94	KDB13524.1	Putative NADPH dehydrogenase C23G7.10c	238.22	61.696	29.9	AAIQIAHAGR ADSLDLIVGSVGNIDTPEFAR EIIEAVR ELSAEEIGALIQSFAESAVR GQAGVPFYTPAQEPAAGTAADPASAPTLFQPLR ISPQDSGLWQDSQIEPLR ISPQDSGLWQDSQIEPLRR IVNYVHSQNTK LLPALGVDVLDVSSGGNSFEQK SLELHNR TYQTNLAR YGGSFENR
95	KDB13436.1	Uncharacterized protein PYUK71.03c	81.873	165.21	5.9	FIVNVYDYDFADKPDFLGATTIR LDSLEPFR LLFRPDYVTR LRVDVLDAEDLPSADR STFQPAELDAPDTPR TLNPTWNEFFEVAVPSR VDVLDAEDLPSADR
96	KDB13380.1	Alpha-mannosidase	53.371	121.34	9.1	APDEHADMGR ELPEDSAEQNHALSVAMR EVIEPGGQANK FADLSEHNYGVSILNDSK IEWNLPEAFR LGLTVDDFFER QLYPGAFER VDSSSLLVFGK
97	KDB13367.1	Phosphomannomutase	11.935	30.152	8.1	GTFVEFR LGASPEILATLAALR
98	KDB13281.1	Rho GDP-dissociation inhibitor	46.175	22.047	35.4	DISDPNDSR DPVTIDLSSPGSETSLK FQEEEAPSGMLAR GHYSAVSSFVDDDGK VQHEILSGLHYVQVVK
99	KDB13253.1	Probable Rho-GTPase-activating protein 7	26.511	83.825	8.8	DLPDPLLTSEYHDAFVR DSLHGIINSLPDPNYATLR GMFDAESSNPALDFR NPENFYHDVNSVTGLLK
100	KDB13184.1	Single-stranded TG1-3 DNA-binding protein	62.055	38.427	27.3	AAANEAASASAAEGR AIAELSGK GFGFVTLASEELQQK IALRPIPR SYLVESVSIPK TDRPVGYAFVDLSTPSEAER VMVANLPYDLTEEK VSVQLAR
101	KDB13150.1	Isocitrate dehydrogenase [NAD] subunit 2, mitochondrial	6.2275	40.984	2.9	FAFQQAQAVGR
102	KDB12951.1	Probable argininosuccinate lyase	72.297	52.112	28.8	AEAECGIIMPGYTHLQR DLQESWEPMLDHVK EDILGSIAFAR FTGGLDPLMLK GILTTLAVKPER NPDGLELLR SEELGIPMNELSLEQLQAIDSR SEFTEIER TPAENMLWGGR TVSDSLQIAK YNASISYDK
103	KDB12801.1	NADP-dependent 3-hydroxy acid dehydrogenase	12.523	29.145	7.7	DFVTNLPQDWR LDVANAQEVR
104	KDB12701.1	Sec14 cytosolic factor	54.346	39.107	27.3	GWLDPVTVK IAQVHQLR LDEVVPVWDYPEK LDTLTLLR LMLEAEGFK MLTNLAVEYER QASVISQNYYPER YDNYDFPTTAAEPQDGHAGYLTPEK
105	KDB12650.1	Uroporphyrinogen decarboxylase	37.225	38.202	23.8	ELAYVYDAVALTR GWIANLGHGITPGVDPDNLR HFFEEIHR LPETLDAMGLDR

						PLDAPTDGQYER VVFQGNADPGVLYGTK
106	KDB12580.1	26S proteasome regulatory subunit rpn2	13.025	125.63	2.1	EGDDKESSPSDAK ESMDLDPAPSK
107	KDB12537.1	Putative dihydroxy-acid dehydratase, mitochondrial	27.724	67.258	7.2	ELNAALAAGAVGR GNLAPGGAVAK GPGDVLADVER NMPGVVMAAAR
108	KDB12473.1	Acetyl-CoA acetyltransferase	14.271	40.736	5.6	AQAAAEAGLFR EIVPVQVAGGR
109	KDB11862.1	Putative glutamine amidotransferase-like protein C13C5.04	83.089	28.491	37.2	ALLASAAQTLSPPR AVGAGVGK DLDAILTTSAHDVVNDLR HTAFDNDPWILR LAILECDTPQPQTR RAPIPHDGVAVGK SYPDLDNVDALLLTGSR
110	KDB11805.1	Isopentenyl-diphosphate Delta-isomerase	47.941	29.56	34.6	APSDGQWGEHEIDYILFIK KLEHELGIKLEHELGIKK LFPDIDTSSEALSGHDEEQIR LFQDPSLK NELLLQQR NEVQATQYVGADELK YTDEQQIR
111	KDB11701.1	Putative D-lactate dehydrogenase C713.03, mitochondrial	31.157	90.216	7.8	APATGSTAIPTR IQLLDTHAALAFAGLNADAR LSVEDPVTIDYITK NYKEEMDR SLLEVVQTGAK
112	KDB11395.1	Lactoylglutathione lyase	36.456	36.489	16.2	EGIIELTHNYGTENDPTYVVNTGNAEPHR FYEFLGMSVIK FYQEVLGMTLIR
113	KDB11291.1	Putative glycine dehydrogenase (decarboxylating), mitochondrial	6.1729	117.48	0.9	SHLEPFLPHK
114	KDB11239.1	Probable histidinol-phosphatase	8.2751	35.879	3.8	AAVAAAGGTEER
115	KDB11194.1	Translin-1	13.098	27.019	9.2	AVATAQGLLAR RVEDVVYDLSLR
116	KDB11054.1	Probable UTP--glucose-1-phosphate uridylyltransferase	18.538	56.154	8	DGMSFLDLSVR KGESDISILQLETAVGAAIR LLEIAQVPK
117	KDB10792.1	Saccharopine dehydrogenase [NAD(+), L-lysine-forming]	46.322	55.206	14.3	ALIEAGYVVR APTSDIILGLK AVGADIVPAGSWVK IIAIDYLPTLVAR VIVIGALGR VPPFVTFESLSGPGR
118	KDB10744.1	Probable succinate dehydrogenase [ubiquinone] flavoprotein subunit, mitochondrial	12.667	58.723	4.9	GEGGYLLNSEGER GVLAYNQEDGTLHR
119	KDB16126.1	Transketolase	184.17	74.869	38.5	AHSEIDQLAINTIR ALPNCMVWR AVATVDFWK EYADLQR FGASGAYK IESSIPELFGGSADLTGSNLTR IVSIPCFEVFDAQTK KFEFTPEGVAK LIWVATHDSIGLGEDGPTHQPIETLAHFR LSALSGVR LSETVLSK LTTTIGFGSLQEGTHGVHGSPLK NLPVYTPSDSAVASR PADGNETSAAYYVALTSK QNLPQLEGSTVDK SAVDFQPEATGLGNYAGR YEAYGWHVEHVQDGNTDLAGMEAALK YTHEQFGLNR
120	KDB14060.1	Cysteine proteinase 1, mitochondrial	47.054	119.22	5.9	ALVLSDNGR ISTSAATGQYR ITETRPR NVTYINVDMETLK QSLPVIDQSNLWAR YLGVDLFTK
121	KDB19054.1	Aminopeptidase 2, mitochondrial	9.6163	98.587	1.6	TDALWAALAEASGR
122	KDB19042.1	Pyruvate dehydrogenase E1 component subunit alpha, mitochondrial	12.647	45.234	4.9	GPMVLEYVTYR SIIGELLGR

123	KDB18874.1	Vacuolar aminopeptidase 1	85.687	60.887	20.8	ATTGALDPGLGVQFFK AVEALSGDAFGPGLAGQTYAR GFLDHWEQIDGEWS GNFLPLVVER LVALFDDEEIGSLLR RVGEIIGAK SGGTVGPMLSSAMGVR TQNFQIR VGEIIGAK VVVRDEETGK
124	KDB18149.1	Allantoinase	37.289	54.772	17.4	ADDADVTAYQTFLDSRPPALETAAVR DAATTLMFHAEKEEDVEHQQQR GGAPAIADIVR GPLVLVSSR ITSVVPAVLPR TAWEGFWTGTR
125	KDB18139.1	Adenylosuccinate lyase	7.2922	31.554	4.9	AHLTVSDEDFETAR
126	KDB18031.1	Branched-chain-amino-acid aminotransferase, cytosolic	25.342	48.875	9.1	AVSLEATDYAVR ELITAPLDGTILEGVTR ETEESGETALR
127	KDB17178.1	40S ribosomal protein S4-A	17.99	88.293	3.6	DAIDNSFATR HDGGFNIVHIK IELATGK
128	KDB17092.1	Rab GDP-dissociation inhibitor	33.436	67.579	8.4	NDHYGGEAASVNLETLFK QVAGSFVQQGAAPK SPLMGIFEK VPSDAGEALK
129	KDB16967.1	Mannan endo-1,6-alpha-mannosidase DFG5	78.939	45.364	24	ALSNPQGILEETDK ALSNPQGILEETDKCELR DLINDGLDSK DVFNDMQTGR EFEQFILR GTNCGGGIYWNK NADSILAK SFDVTGDYQYLEAAK SGLINSK YLNQVAPHR
130	KDB16801.1	Elongation factor 1-gamma 1	77.5	46.433	29.1	AFGTLFTSADNSR ANNLDLK ATFPLDEYKR GQEHEPAFDVAPDWESYK HPLDLLPR ISEVEFGKPTAEHLK KNVDDGAQATEK LDPNNADDR NVDDGAQATEK QDYASILR QFSNSETPEALK QQHPNVTR
131	KDB16714.1	Probable dipeptidyl peptidase 3	222.57	78.746	32.2	AFGELVSAAPTLLPLLPWDK AGLASIELWDPK DDLSDLTIHLDR DTEFTVGNGLLK ENPPVSPLTQK GEWEGFASMVNR GLLPENNR HFLADQPPTVVR HYDASLVGIIQSWADR LLQETSPGVFNFDK NAVADYLR NVSLGNVVSAK QAADNADNDTQR QISPESEPIYDLIISLHNSCR SFETGSLEAFK SVAALAATSPEAAK YRDDAFEVQVGLHELTGHGCGK
132	KDB16561.1	60S ribosomal protein L20-A	10.875	20.974	12.9	ATGEIVSVK HLPTEANPTPDMYR
133	KDB16363.1	UPF0045 protein ECM15	10.898	9.4867	23.3	VLGASGLK YTLHSAGTTVEGR
134	KDB16280.1	Saccharolysin	81.857	101.44	10.7	AYTIAESNK GGSQEELDTLR GPEEQGQGYEWGNR LAPGGAQEAGR LVNASYASR VMDFLGDLR VVAEVKPEDAVFANVLEPSLASANR YTHYFPLVK
135	KDB16191.1	60S ribosomal protein L19-A	11.652	23.669	9.8	EQAIKDEMDAK LVADGLIIR
136	KDB16148.1	Proteasome subunit alpha type-1	20.34	27.679	8.3	LANISQVVYTQR MLTEDEIDDR
137	KDB16051.1	Argininosuccinate synthase	69.037	44.919	20	EANEIGR FAEEQNIPVSSTPK GCAYVVGR GCYDTPGLTILR QAHLDLEGLVMDSK VLAPWR VVTGSLEIFK YLLGTSLAR
138	KDB15720.1	40S ribosomal protein S2	18.258	27.725	17.6	FLQLAGVEDAYTSSAGSTK INSMEEIYLHSLPIK SPLEEFADTLR
139	KDB15701.1	60S ribosomal protein L8-B	14.664	27.036	9.2	NYGIGQDIQPK TAAVLALTEVR
140	KDB15640.1	Cyclin-dependent kinases regulatory subunit	7.1651	13.394	11.6	LDEYIDSIHYSAR
141	KDB15345.1	Fructose-1,6-bisphosphatase	11.892	57.429	5.3	KLDVISNDLFIEAMR TLLYGGIFAYPADK
142	KDB15264.1	General amino acid permease AGP2	6.6276	61.773	2	PLIDRYEADLR

143	KDB15112.1	HD domain-containing protein YBR242W	11.961	30.381	7.5	DDAAVGQEIR GVQGGVTEEK
144	KDB14495.1	Proteasome subunit beta type-3	7.6005	21.15	6.8	TFANLVSSSLYER
145	KDB14476.1	Protein HMF1	58.382	18.556	40.1	CNIFLSDMAHFDEMNSVYEK DAPAPVGPYSQAVK FFAHKPAR TPTAIYCSGQIPLKPDGTMVEGSIADK
146	KDB14458.1	Bifunctional purine biosynthesis protein ADE17	10.538	64.39	3	HVSPAGAAIGLPLSPEER
147	KDB14326.1	Niemann-Pick type C-related protein 1	57.059	140.6	4.7	AMDLIGGGAK KCNDEDPDYR LLVTELDHLISER SELGTPNTLIGSCPACK YGSGLYDSCK
148	KDB14303.1	40S ribosomal protein S8-A	19.967	23.35	19.4	QWYEAHYGQPIGR SAVVQIDAAPFR VIVVAYHPSNNELVR
149	KDB14278.1	Malate dehydrogenase, mitochondrial	89.779	34.907	37.3	DDLFNTNASIVR GADIVLIPAGVPR GGPGVAADLSHINTK GIVEPTFVDSPLYK HPDLSSNAELVK LLEACLGDLK TLFGVTTLDVVR TNPAEENITVIGGHSGVTIVPLFSQSR VTDLALYDIR
150	KDB14189.1	Aminopeptidase 2, mitochondrial	323.31	102.41	49.5	ADEINQIFDAISYSK AFPCFDEPALK AGMIADAGALATSGYQR AMLFEGAGLSGDEQIIDASK ANQPELIK AVTEGLK DGLLSVEDR DGVDGQIALTER DQDVYMPVIGLR DRDDVAAWVK EFVVWNEITSR ELLPANVVPR EVGYPVLTVTENDADSTIHVK FEFEGTLPK FTVTLVADK GLDSEKEFVVWNEITSR HAYGNTQTGDLWASLSAVSGK HAYGNTQTGDLWASLSAVSGKPVEDVMR HYHLTLEPDFK HYHLTLEPDFKK LGAIQSAWVFEDK LNANHTGIFR MGWQFSENDGHIEQQFK MVSTYLGEDVFLEGVR PVEDVMR RADEINQIFDAISYSK RDDGSEGILATTQMEATDCR SSHPIEVPVK SVFAIALK TGDATPEDDKVLYPVFLGLR THAEGIESLFEWMEAR TKDGVDGQIALTER TLGLLFGPDVK TSGVLNLLK TSYTPSR VFGIEFPLPK VLYPVFLGLR VPSTGFFK VVDLLLDEK VWENYVTDNLQSALSLDSLR VYAPPGQDIEHGR YGGVEEYDK
151	KDB14026.1	60S ribosomal protein L9-B	19.482	21.886	17.2	ETGLWEVEIR IVTVEGPR YIHSQELLEIPEGVK
152	KDB14012.1	Cystathionine gamma-lyase	6.6112	44.636	3.1	LIWIESPSNPTLR
153	KDB13850.1	Metal homeostasis factor ATX1	6.4536	11.761	11.6	VVTELPYETVLSK
154	KDB13732.1	Probable proteasome subunit alpha type-7	12.239	19.028	14.9	LDLQSETMTLEEAVK TPIPTADLASR
155	KDB13578.1	Glutamine--fructose-6-phosphate aminotransferase [isomerizing]	11.687	74.58	3.3	AVIQELEGAYGLLIK SLLLLGR
156	KDB13577.1	Proteasome subunit beta type-7	12.546	28.885	10.7	HLTELALAEAYDSPDAAR YAPDEESAAK
157	KDB13484.1	6-phosphogluconate dehydrogenase, decarboxylating 1	163.99	57.983	43.5	AAELGIPTPAFSTALSWFDGYR AIHAAQPGWR ALGLSNK DIHVNWTGR DIMYFNDDDGKPLVEK DLPANLLQAQR DYFGAHTFR EQFLEDLEQALYASK FEGNKEQFLEDLEQALYASK FVGSGVSGGEEGAR GDIIIDGGNSHFPDSNR GVLDSFLIEITR ILDQAGQK LLPLLEK NLLFDDFFNK PVDEWIEK SIVGAQSVEEFIGK VDHFLANEAK VMLLVQAGKPVDEWIEK YGPSLMPGGNEEAWPHIK

158	KDB13437.1	Proteasome subunit beta type-5	7.3755	30.976	4.2	HGFTDTNPVFWK
159	KDB12840.1	Probable alanine aminotransferase, mitochondrial	76.092	59.223	24.5	ADGLDVR ALEAAAAEK AWGTNFEAIK DGFPADPSK DSIAAFLER DTINQHVVLAEYAVR EYSAIYDGLR MQQESPGR QVGSVGAYSASAGVPAIK TDVISR TTFLAPGTEWVAGVVK YDGLELASLHSISK
160	KDB12786.1	2-oxoglutarate dehydrogenase, mitochondrial	15.491	118.34	3.4	IEQLNPFPWQQLR NIDFPTAEALAFGTLVTEGYHVR
161	KDB12590.1	Peroxiredoxin PRX1, mitochondrial	97.11	24.239	67.9	ALFPNLR DIAEVTGGQVK FPIVADPDR FPIVADPDRK IATPIDWLPGQDVIVANSVK IVDSLQAVDK LGSQAPGFTADSNVGPIDFHK LLGLSADTTESHETWIK NAAEVLR NSLPLTIR SVFFIDPR TILSYPASTGR VANLYDMIDYQDPQNIDR
162	KDB12482.1	Phosphoserine aminotransferase	52.87	46.314	23.9	AFLEEGLAQGLTGLK AQLIYDAIEAFPEIYR FGTIPDESTWK GDDIDAAER NLGPTGITVVVIK SFLPPVTSQPPSSLMR SQLASDIINQTK VEGQQAVSEK
163	KDB11596.1	Probable quinone oxidoreductase	40.717	34.89	22.8	AHEDIESR NAYAGVNFIDTYFR TGGVDVLELKDDVPVPTLSSGQVLVK TGLYPASLPLTLGR YASELFELVSSEK
164	KDB11345.1	Fatty acid repression mutant protein 2	40.847	22.961	27.6	AHMNYGDEAQPHPEKPSK ASAPLTADQFLAAASHR ITLITADK QFWDVISAAAEPVLK
165	KDB10988.1	Obg-like ATPase 1	6.8575	43.797	2.5	DLDIISEELR
166	KDB10968.1	Cell wall mannoprotein CIS3	42.118	37.112	21.2	ACGGEGTLIMTLHDGVLK CLSGSFYNLYDR KPDSPPPAGCSTSYDGK NWAAQCQPIEMVVTSCDAGNNPPSGGSGGK
167	KDB18200.1	3-isopropylmalate dehydratase	18.184	84.806	4.4	ALVVPGSGLVK EFGLTYFGLGDK EMEVDLPNQQIR
168	KDB17534.1	Superoxide dismutase [Mn], mitochondrial	57.876	25.048	43.2	HHQTYVNSYNAAIEALDK LLAAINEDFGSFEDLK LLAAINEDFGSFEDLKK MSGTLSLVTR NDVTAAAAQAPLINFHGGGHVNHSLFWENLAPNGK QTNAALAGIQGSGWAWLVK
169	KDB16669.1	Glutarate-semialdehyde dehydrogenase DavD	90.286	53.728	24.5	AAADAFPAFR APGETPYTALAVAELGHR GVVNVITTSANTPEIGEVLTTDPTIK GVYEEFVGK LIGTCAECDAQDTR LPDLGANFFQPTVIR PATSGETFEVNDPSTGK TYGDTIPATVPGNR
170	KDB13689.1	Cold shock protein CapA (Fragment)	112.42	69.247	13.3	DGQAVTFVAVQGQK GFGFITPDDGGSDLFVHFR GIEMEGFK IVSEQPTAK QLPLGFAPEQSWR VAFHPYAPDLFSPAFSADDYPK
171	KDB16823.1	Alkaline phosphatase H	171.32	75.941	33	ALGDLLELDDTVR ALGVFCQSNLPVWLDR AVEVLHK DAIGVYEQSGLSQYTVKPK DIAAAFK DVNYGTGVNFPANWEPR EPALDLPGLK GAWGAVSTAFLADATPTALTGHTR GFFLMSEAASVDK GGKEPALDLPGLK KTPSVVNVSSK LSLDEPGK QMHALDYDR SSDGYVVNPK STVNAMGVHADSSPDPFDDPKVETIVEIFR TPSVVNVSSK TTAEWVVR TTAEWVVRPIAK VEEPPLEK VETIVEIFR YAIAGGVGAAPDR
172	KDB10918.1	Peptide-N4-(N-acetyl-beta-glucosaminyl)asparagine amidase A	157.81	75.324	29.4	ADLTQGMDMTVLGR AEASVLLPR AHPGIAWTYWK ALEDGSTVYHAAYSYPTLAR DEDLSGAAGR DMTNYLALWR GSSSSQGSAWTYPEQK IFVWLDDGPGHVTSGRPPR LATMWLGDAEVWR LIFDLGNLINHK NLGNPR PADHVLPISAGR PMVGPQAFDLR RPVVEVARPPAAQGDSPNGFAVR VLQAESR VLVSAPDYEPR
173	KDB13378.1	60S acidic ribosomal protein P0	7.9244	34.455	3.4	TFINDTPEYER

174	KDB17762.1	Elongation factor 1-alpha	61.2	46.428	21.2	IGGIGTVPVGR LPLQDVYK QLIVAINK RPTDKPLR SGDSAIVK SVEMHHEQLTEGVPGDNVGFNVK TLLEAIDAIEPPK YYVTVIDAPGHR
175	KDB17697.1	40S ribosomal protein S9	18.061	21.98	17.3	HIDFALNSPFGSGR QIVNVPSFIVR VGVLDESR
176	KDB12335.1	40S ribosomal protein S15	18.787	17.43	10.5	GIDLDNLLDLSSDQLR
177	KDB11515.1	Heat shock protein 90 homolog	276.11	80.256	35.6	ADLVNNLGTIAR AILFVPK APFDLFETK DEQAEYLNESK DFELEETEEEKK DTGIGMTK ELVSNASDALDK GTSIILHLK GVVDSEDLPLNLSR HSEFISYPIYLHVTK IDIIPNK IEEVDDEDEEK IEEVDDEDEEKEK IEEVDDEDEEKEKK LGIHEDSQNR NMYYITGESIK NPQDITQEEYASFYK QYESLAK RAPFDLFETK SGDEQTSLSDYVTR SLELFNEIAEDK SLELFNEIAEDKEMFDK SLSDPSQLDSGK SLSNDWEDHLAVK SPFLDTLK VKETTIEEEELNK
178	KDB10832.1	Histone H2A	6.2585	14.227	6.7	AGLAFPVGR
179	KDB12033.1	Cellobiose dehydrogenase	75.582	60.657	21.9	AVHDWIVGSAGYK DAQTIASFIHSLFQR ILASMR ILMYSGIGPR LPPSTPNSPAK LSEAGLK PSSGIYYGHPR RQPPLTHYSPDGK SYGIVGGR TLLLEQGGK TYLQSALAR VDVPGLFSSIFR
180	KDB12171.1	Protein SnodProt1	14.198	14.293	16.9	VDATVTQLSAR VSWDGGYNEAGR
181	KDB14079.1	Probable beta-galactosidase A	119.77	112.24	20.2	AEGIFDLDPFFDAAK AGIYLLAR AMMFSGEVHPFR ASSIQDLVSWDEHSLFIHGER GGTLSLYGR GPLNEGGFFFER IDGSSILLHEESSSTIVQWKPGPK IHLTDYPVGDYK ITGNLGGENYVDK LPVPSLYLDVFEK LPVSPNGMLTIPQR PGPYINAEVSGGGFPGYLQK SAGISVPMINNDVGPSGNYAPGK VVLVTGPLYSK
182	KDB18706.1; KDB18773.1	Homocitrate synthase, mitochondrial	18.365	44.746	7.3	MIVGSHEYVTSR SDLVDLLSLYK SEGLEVR
183	KDB16852.1	Importin subunit alpha	10.241	60.341	2.2	QAAGEGAEAINR
184	KDB11829.1	Beta-1,3-glucan-binding protein	34.517	35.283	25.8	DVGDEDAWEAMSAGSK GVWHTYAWEMNR HWGTWEIETYTDDPQNLYITHDDTLR IETTPEWDTACEPGHR YDLGTSYPGGPK
185	KDB18653.1	Proteinase T (Fragment)	20.434	39.383	9.9	AAAAITK DDNLADYSNVGSLVDVLAPGSK ILSTWIGGR
186	KDB11595.1	Bifunctional protein GAL10	19.663	40.06	10.1	AFSAVVGR AVGESGEIPLEYYR SMIEDVITDWVNAQR
187	KDB16107.1	Neutral ceramidase	34.296	81.348	6.1	AFIVADK GVAAWMFENSVK QEQTFAAIER QGSDGSWSR YGVLDGLK
188	KDB12890.1	Probable (S)-ureidoglycine aminohydrolase	62.278	31.153	39.6	DVAPAAMPGTHGAWATTR EYEAVPGLDPPDPLFLNER FVDPADLR GNNDTVGPYYAPTGGLPPQTQLLTDR KEYEAVPGLDPPDPLFLNER PLSGFAETFSQYIMEVQPGGGSDR THTLAEGGYAYLPPR
189	KDB13798.1	2,3-bisphosphoglycerate-independent phosphoglycerate mutase	74.212	58.517	23.2	DQDQDLVPSNK EVGIGQIATVVGR IKDDDTVFFFNYR NEELSENEVIK NEELSENEVIKK NGDQDRDEFLTPIIVGGGESR QITQLLGDVDR SGAGYMQELVEYIK SVLPDFSYPK VFIHFFGDGR VVWQDVVR
190	KDB18825.1	Probable Ras-related protein Rab7	7.0909	22.971	6.4	DPINFPFVVLGNK
191	KDB18767.1	V-type proton ATPase catalytic subunit A	67.022	63.456	22.6	AIAQGQSWAK EATSDLQAK LAADQPFLVGQR LGEMPADQGFPAYLSSK

192	KDB18497.1	60S ribosomal protein L3	11.366	44.066	5.1	QLISDSEELDQVVQLVGK TVISQSVSK VGDHISGGDVWGTVFENSFISTHR VGHDSLAGEVIR VQALGSPER VSQTIYIPR SMFTHTSR YGEINNDFVMLK
193	KDB18386.1	Glutathione reductase	170.65	47.041	47.6	DGQGNESSVSDVDHLIWAVGR HFDPMIQESVTK ILIAVGGR KLDYSNIPSVVFAHPEVGSVGLTEPQAVEK LDYSNIPSVVFAHPEVGSVGLTEPQAVEK LFGPAEFSTR LIVAGPDEK LVSSNQVEVTQDDGSK NLGNDKVEYLHGR PSSPPQIPGAQLGINSDGFFDIDK RLNGIYER SYGFSVQQTAPFDWTTFK VLVNAK VPETQHIGLQEAGVK VTFNAAAIAETLHDAK
194	KDB18363.1	4-hydroxyphenylpyruvate dioxygenase	68.223	45.514	33.1	AGYAGPFLPGFR ALLADMHAHLER AVAQGAEAVR DVAFEVDDVPAVYAR GVDFISVPATYYATLR MPINEPAPGR NQFEGFGAGNFK SLAHLPADEDISPDDR SLFEAIER TADPHGSVVSAAIR TYGDTTHTLVSR
195	KDB18340.1	40S ribosomal protein S5	20.212	23.648	17.4	QAVDVSPLR SIAECLAEELINAAK VNQAIALLTTGAR
196	KDB18338.1	ADP,ATP carrier protein	46.094	52.332	9.7	GNTANVIR SGDAQPK TAAAPIER TLASDGIAGLYR TMADEGALSLWR
197	KDB18313.1	Heat shock 70 kDa protein	195.63	71.573	42.8	CDIIANDQGNR DAGLIAGLNVLR DLSTNSR EEDEAEGR EEYEEHQK EEYEEHQKELEAVANPIMMK ELEAVANPIMMK ETAESYLGTTITNAVVTVPAYFNDSQR FELTGIPPAPR FNDAEVQADMK GGKPLVEVEFK IINEPTAAAIAYGLDK IVAWLDDSQQATR LITDYFNGK LVNHFVNEFK MLSDAEK NQVAMNPHNTVFDAK SINPDEAVAYGAAVQAAILSGDTSSK SQVHEIVLVGGSTR STAGDTHLGGEDFDNR STIQPVDR TFTPEEISSMILTK TTPSFVAFTDTER YKEEDEAEGR
198	KDB18285.1	Cystathionine gamma-synthase	25.935	69.89	9.3	ELEWAAEYGVVEHLVR ETALPMELALDAAK IQALYTEFPGNPLLK RVEIANQNAEAVAR
199	KDB18196.1	Phosphatidylglycerol/phosphatidylinositol transfer protein	74.399	18.613	48.3	EIPPGTYNVVAHVFAANDDEITCLTATVK EYVEINSVDLVPNPPAAGK TLDLCEQMGEVDLECPVQPGER VPGESPLEYCTGQETK
200	KDB17961.1	Heat shock protein hsp98	42.178	103.2	8.7	AHGDPQLFDR DSYIGVDHLITALAEDHSIQTALR ESQPEVIDSLER NVEEELSPLR TADTDQENENLAK YGAIPEQQAVIK
201	KDB17625.1	60S ribosomal protein L7	7.2211	31.795	4	HFIEGGDLGNR
202	KDB17624.1	40S ribosomal protein S14	11.763	16.686	15.3	IEDVTPTPSDSTR TPGPGAQSALR
203	KDB17571.1	V-type proton ATPase subunit B	44.158	56.583	15.9	AVVGEEALSAEDK LGVSEDMLGR QFISQGAYESR SGQVLEAR TTLFLNLANDPTIER VEFTGESLK YNEIVSLTLPDGSVR
204	KDB17548.1	Probable cytochrome b5	42.637	15.117	24.8	DIYVVIHDK QPGDPAPK RQPGDPAPK SAALELSYQDVAEHNTK
205	KDB17437.1	60S ribosomal protein L10a	6.3974	24.094	6.5	HGGVDAMSADDLKK
206	KDB17426.1	Carboxypeptidase Y homolog A	56.96	52.777	21.2	GGHPAVLDQQSCR QAGDWFLPIHR QDFHISGESYAGHYIPIFASEILSHK QYSGYLDDNATDK VDPSSLGVDTVK WPGHDAFNSAQSVELTLGPSPAYGTIK
207	KDB17394.1	60S ribosomal protein L17	6.5281	26.373	4.3	SAQYLLDLLK

208	KDB17296.1	Peroxisomal hydratase-dehydrogenase-epimerase	47.452	96.572	11.4	AAANYNSVEDGDKIIETAMQNFGR IIETAMQNFGR RAPDAVVEAK TGGHGFPVNVALTPEEVVK VALVTGGGAGIGR VDVVINNAGILR VVDFSKPQYPTGPNDFLGLLEESQK
209	KDB17270.1	Serine/threonine-protein phosphatase 2B catalytic subunit	23.79	67.094	7.1	LFEVGGDPAETR LTEEQALWILR TLQVDNAIR YLFLGDYVDR
210	KDB17192.1	Malate synthase, glyoxysomal	80.872	60.516	28.8	AGHDGTWVAHPALAAIATDVFNEHMPGPNQLFAR DDLLNMTVPGSVTEDGIR DFVLPDR GAPPAPGLVDR GVHAMGGMAAQIPIKDDAEANAR GWHLDEK LESHLEAR LWNDAFNLAQDR RDDVGSIGR RVEITGPTDR SQLWQWVR TPPTLIVR
211	KDB17180.1	60S ribosomal protein L24	6.2205	17.958	5	SESLFLQR
212	KDB17022.1	Peptidyl-prolyl cis-trans isomerase, mitochondrial	23.334	19.601	14.4	IIPDFMLQGGDFTR ITFDLFDDVVPK
213	KDB17017.1	Kynureninase 1	10.955	55.637	5	LEPDAGGLIPTDSILR VAPVPLYTR
214	KDB16989.1	FAS1 domain-containing protein NCU02579	6.351	25.502	4.7	LILPDGVEVDR
215	KDB16625.1	Protein disulfide-isomerase erp38	45.232	40.277	22.3	ENDVSSYPTIK FDVQGFPTLK KGEIYSGGR LEMPSEVVMLTDK NLAPVWESVAVDFANDANVVIAK SLAPVYEELGLAFEHAK
216	KDB16573.1	Phosphoenolpyruvate carboxykinase (ATP)	11.469	65.782	3.9	AWTAGAESFQNEVVK GVLPPISK
217	KDB16500.1	60S ribosomal protein L32	11.639	14.907	15.3	GTVAMPSIGFGSNK HVPIVK
218	KDB16374.1	3-isopropylmalate dehydrogenase	175.41	36.93	47.9	ANVLATSR AVEAAVK EDDGSGTAWDSEPYSR ELTGGIYFGER EVGDEVVK FVLNEQLLGGCSIDATGSPLTDEALNAAK GTDIVIVR HLITILAK IEHQLIDSAAMILVK KVMTETFASEFPDLK LAGFLAR SADAVILGAIGGPEWGTGAVRPEQGLLR TVWSLDK VMTETFASEFPDLK
219	KDB16190.1	Putative serine hydroxymethyltransferase, mitochondrial	30.697	53.088	17.1	FADIVTTTSHK HFINLIPSENFTSQAVLDALGSPMQNK LDETTGLIDYDKLEELATIYRPK YSEGYPGAR YYGGNEFIDQSER
220	KDB16187.1	Guanine nucleotide-binding protein subunit beta-like protein	28.468	31.899	16.7	FSPNPQNPVIVSAGWDK LWELATGTTTR QIVSGSR VDELKPEFTAVGK
221	KDB16142.1	Spermidine synthase	99.188	36.342	42.5	ASFVLPTFAQK EISEMWPGQAMTLR HDCVEEAILCDIDEAVIR KPYFQLLHDALR QWTPEEEEAK QYLPNMAEGFSHPK SQYQDVLIFK STHHGTVLVLDNVIQCTER VHVGDGFK VLVIGGGDGGVLR YYNAEIHK
222	KDB16090.1	Mitochondrial outer membrane protein porin	17.849	35.971	7.4	AANDLLNK ATSAAIEGK IDPVSFAK
223	KDB16050.1	Enolase	228.39	47.025	48.9	ACNALLLK AIVPSGASTGQHEACELR AVDNVNSIIGPALIK DSYADGWGVMVSHR GNPTVEVDVVTDTGLHR GVPLYAHISDLAGTK IAMDVASSEFYK IEEELGDGAIYAGANFR KYDLDFK LAFQEFMIVPSDAPSFSEAMR LNQILR SGETEDVTIADIVVGIR SQDIQIVGDDLTVTNPLR VDAFLNSLDGTDNK VNQIGTLTESIQAAK

224	KDB16006.1	Isocitrate lyase	58.383	60.315	19.1	AIASSSGDQAGLR DHAYILGSTNPNLQPLNDLMVAAEK IEDEWLAAAALMR IEYPSSAQAR MVTGGVSSTAAMGK RPFTAEQIVSK VGAELQR VLVPISEHISR
225	KDB15899.1	Heat shock protein hsp88	270.25	79.799	47.7	AADIPDEDTSLTVFNK ALAGPIVQR ALLDAAEIAGLK ALVDHLAK ATAWDHTFGGR ATPSMVGFGPK ATQQDGGDQEMK DGDAMDTDKK DGDAMDTDKKEDAPK EAAMVMEDK EDIDIIEIVGGGSR FTSTQLIAMFLSK GNVLPSTKGVDVITNEVSNR KGDLPISSGTSSLDAAQK KNELETYIYDLR KQPFDLEAR LDDQYAELATEEEK LDLPAPEEPPR LDLPAPEEPPRR LEAAEDWLYDEGDDASK LINDTTAAALGWGITK LTKEDIDIIEIVGGGSR LVADTEEK PLSYTMNADEAVAR QEFEAMIEPLLNR QPFDLEAR SVVGVDFGTLK TQEITNLK TQQPLEEVLAQAK TVVAVAR VDIYTHGR YARPEELPGK
226	KDB15865.1	Nascent polypeptide-associated complex subunit alpha	6.2725	22.445	6.8	SPNSNTYIVFGEAK
227	KDB15300.1	Probable small nuclear ribonucleoprotein F	7.1303	9.7001	16.1	PMLQDLVNKPVFVR
228	KDB15252.1	Dihydrolipoyllysine-residue acetyltransferase component of pyruvate dehydrogenase complex, mitochondrial	34.379	49.236	13.5	DVPVGSPIAILVEEGADVAAFEK ENNLSLDGVK FTLEDAGGNAQPAAQPK VVDGAVGAEWIR
229	KDB14978.1	Leukotriene A-4 hydrolase homolog	203.97	71.219	41.9	CTALQWLTPAQTSNK DFYHPICR DPTTLSNYSAWR DVEAFTANQK DVSCVYGVAELLGSVGR ELALETFAK FIPHYFAK GFHFLYYLDR IATIDWQDR IVFLDK LIISHR LPVVASGVPVGDHTPAVGAEK NVDPEDVYSSVAYEK SIFPCQDTPDVK SLDSFEFR SLEDAVALFGK SVVVTGPNELEGCK VALDAK VDSAECAWELRPHSDPFGAPLHVAVPEGAAK VPIPSYLFALASGDFASAR VQESASLSASR VYDFVSSK
230	KDB14796.1	Inorganic pyrophosphatase	109.84	33.006	63.1	ASEYSLR DGVPVSPFHDIPLYANR DQTVLNMVVEIPR EELLNPIK EYAMDVVR GVSIANTTVAHSADR GYLWNYGAFPQTWEDPNVVHPETK IDSSIDK IPDGKPENQFAFTGECK LNDIEDVER LNPEQLPPLPPHQELPAEK TGAANTFEHR VIVVDVNDPLAPK VLGVMALLDEEETDWK
231	KDB14658.1	Succinate--CoA ligase [ADP-forming] subunit beta, mitochondrial	19.31	38.718	8.2	DTTQEDPDEVR FGFDDNAEFR TVENLNLK
232	KDB14601.1	Citrate synthase, mitochondrial	29.672	10.737	34	AIGVLPQLIIDR AVGAPIER AVGAPIERPK IAPGVLTEHGK
233	KDB14600.1	Citrate synthase, mitochondrial	86.755	41.904	23.7	ALVWEGSVLDAEEGIR DFIELLR DLSAEWAAR DYLWSTLNAGR FAELLPEK KVVGDDLSDK LPNIAAR VTLDQVYGGAR VVGDDLSDK VVPGYGHAVLR
234	KDB14596.1	Plasma membrane ATPase	30.908	100.05	6.1	GYLVAMTGDGVNDAPSLK QLGLGTNVYNAER SLEDFVVSLQR VGAPALDTNIESGK
235	KDB14283.1	Hercynylcysteine sulfoxide lyase	27.299	50.901	12.4	DTDALVPLEHK LVADALGTEHLDNER TIELAYPLEDEDIVAAFR TTLATSHGYVAR
236	KDB13992.1	Superoxide dismutase [Mn], mitochondrial	19.494	22.75	13.4	ATYVENIWK TWGSLDGFK VINWETAESR

237	KDB13929.1	Probable ATP-citrate synthase subunit 1	65.583	72.164	16.8	DGTIVPQPEPAVPK EAGFHVPNTFEDMPLVLK FGGALDGAADEFTR GQELLYAGLPISDVFK IPIDYSWAQELGLIR TEVQFGHAGSFANSQLETAATK TIAIIAEGVPER
238	KDB13778.1	Arginase	45.559	33.806	27.8	AFSMFDVDR AGVDAGPEALIASGLLTQIQSELGYK DDMLLNVK LVLTLGGDHSVAIGTVTGSAK NPGTVSAVNR YEELMPASDPDHR
239	KDB13470.1	GTP-binding nuclear protein GSP1/Ran	15.421	45.752	5.1	LVLVGDGGTGK NLQYYDISAK
240	KDB13461.1	ATP synthase subunit alpha, mitochondrial	102.92	59.599	25.7	ASPTEVSSILEQR EAYPGDVFYLHSREITPAFIK GVQEESGLAETGR HSLIIYDDLSK ILQWEADYLAHLK LFLAQYR QAVAYR TAVALDTILNQK TGAIVDVPVGDELLGR VHGLANVQAEELVEFASGVK VLSVGDGIAR
241	KDB13460.1	60S ribosomal protein L11	11.673	19.983	12.1	AEEILER VLEQLSGQTPVYSK
242	KDB13389.1	ATP-dependent RNA helicase eIF4A	81.042	44.78	21.7	ALQEGPPVVVGTPGR DFTVSAMHGDMDQAQR ELAQQIQK GHDVIAQAQSGTGK KDELTLEGIK MFVLDEADEMLSR VLIATDLLAR
243	KDB13202.1	40S ribosomal protein S21	9.2701	9.6428	12.6	AMGESDDAINR
244	KDB13193.1	Glucoamylase	323.31	62.635	57.2	AAPACNSDMTFNVK AATAYGETIYLTGSITELK AGVVPPSWGEPASNSVPTSCK ALVEGSTLATR AQGAASGVVVASPSR ATLNDTWR ATTLILYANR AVSAYADGFVAVVQQYTPSSGALAEQFDR AVVDSFR DANSLISVIHTFDPTASCTDATFQPCSAK DLLPSIATGSYAK DSALTFK FIAGDATMQPK GAVETAWPVISNDLAYAVR GVLANIGPDGAK GYVDSNINVNDGR LQGVSNPSGGPTTGGLGEPK LVDQGDAR QYLAAQAK SDPDYWYTWTR SSSPTFR TTSGEFVWLSGPNLSATSSSGCGSR VAGEAAAVGR YAEDVYYK
245	KDB13167.1	Elongation factor 2	178.69	93.369	31.9	AETLYEGPLDDDAAMGIR AFNQFILDPIFK AGIISTAK AYLPVLESFGFNGDLR DLEEDHAGVPLIISDPVVQYR DSVVSGFQWASR EDLYQSFSR EGPVAEEPMR FSVSPVVQR GGGQIIPTAR GHVFNEEQR GHVFNEEQRPGTPLFNIK LYMVAEPIAEELSLAIENGK NDEVTSLLEK SGTLTTSDTAHNLK SGTYEGKPLER STAISLYGR TVLMMGGK VEPIDDMPAGNIVGLVGIDQFLLK VFSGTVR VGTIVTEMR VLADDFGWDVTDAR
246	KDB13083.1	Glycogen [starch] synthase	12.075	79.845	3.1	APVTTAEYGDR GVDMFIESLAR
247	KDB12782.1	ATP synthase subunit beta, mitochondrial	122.46	58.163	30.4	ATDTGAPITIPVGPATLGR FLPIHSEPPEFIDQSTSAEILVTGIK GISELGIYPAVDPLDSTSR IGLFGGAGVGK IHQVIGAVVDVK IVGQDHYDTATR LVLEVAQHLGENVVR TVFIQELINNIAK VALTGLTIAEYFR VALVFGQMNEPPGAR VVDLLAPYAR
248	KDB12690.1	60S ribosomal protein L5	33.803	35.818	19.2	DIITQIVSSEINGDK GASDGGILVPHSEK VFLDVGLAR YIDDDVEADGLEELYTEAHAAIR
249	KDB12532.1	Cytochrome c oxidase subunit 4, mitochondrial	12.208	22.281	16.3	GPSTPNEQAAALAGTVR PGTVPTDLEQATGLER
250	KDB12449.1	Clock-controlled protein 6	34.328	30.177	19.6	CLAAMNDCK CLSAMNDCK CSDEWTACR KCSDEWTACR SKPGANMSFCTSEYVK
251	KDB12314.1	S-adenosylmethionine synthase	18.315	42.93	7.9	SLVNAGLAR SNFDLRPGVIVR SSEELVEIIR

252	KDB12309.1	Peroxisomal hydratase-dehydrogenase-epimerase	38.174	33.968	26.5	FVTEVAGGK GATQEVVDFYASSK GRPGSVLEVETDLVDAATDEVYTR IPSVPEFDYR QAGFEEIR VTSSSFFVGQGNWHGPK
253	KDB11877.1	Glucose-6-phosphate isomerase	157.6	60.926	35.8	APANTLPAWAELQAHR AVYHTALR EAFASDPDR ELDEPDKSEGHDTSTGGLIDAFK FLSGAHAMDR HFVALSTNEAEVTK LIPTDFILAAK MLASNYFAQAEALMVGK NLVTDETVDLLVK NMFGFENWVGGR NSDPETTLFLIASK SHNPISNNLHQK SIASDGSSVK TFTTAETTTNANTAK VDGVDVMNAAGGVK
254	KDB11875.1	Mannitol-1-phosphate 5-dehydrogenase	44.399	43.793	18.7	FIAPVIAK FIGPAAELAER FQNVEGDDESK HEFEEDDQAAYVEK IGNAHLGDAVER IYPQLLQVVK THEEDLVK
255	KDB11692.1	Phospho-2-dehydro-3-deoxyheptonate aldolase	6.9406	53.838	3.1	VGPTTPTSDLLDLLR
256	KDB11558.1	E3 ubiquitin ligase complex SCF subunit scon-3	13.246	19.784	10.6	ENEWAEDR TTDIEEWDQK
257	KDB11557.1	60S ribosomal protein L12	10.204	19.771	6.6	SVSLEEIIEIAR
258	KDB11526.1	40S ribosomal protein S26E	6.2734	14.873	6.9	NMVESAAIR
259	KDB11525.1; KDB11907.1; KDB15905.1	Ubiquitin-40S ribosomal protein S27a	61.434	17.576	42.5	EGIPPDQQR ESTLHLVLR IQDKEGIPPDQQR LIFAGK MQIFVK QLEDGR TITLEVESSDTIDNVK TLADYNIQK
260	KDB11496.1	Succinate--CoA ligase [ADP-forming] subunit alpha, mitochondrial	7.4438	34.501	3.3	ALESAGVIVER
261	KDB11467.1	30 kDa heat shock protein	110.18	23.277	56.9	AFYEDTSSFTPLFR ATVEDVDEGKDK DGILSVVVPK DNVHIEFTEPQTLVVR GAITEGGEGGESHR LLDDFDTYSR SGGAFWQPR TFTFPAPVDQDGVSAGFR TYTAGTPPAGVIEEGER
262	KDB11441.1	Catalase-3	323.31	78.489	44.6	AHLEPYEVDDSHGYLTSDVGGPIEDQNSLK ASLVWDEAQHIAGK AVQQNVLTQLNK DGAGQNFIHK DVHGFATR FSTVAGSR GFFTAPGR GFFTAPGRPVTGALGR GIDFTEDPLLQGR GPTLLEDFIFR GSGIYAQK HGGPNFEQLPINR HGGPNFEQLPINRPVVPIHNNNR HMDGFGVHTYR IFSYLDTQLNR IIPQEYAPLFPLGVMR ISHDIAVR ITHFDHER LDANPTNFFAETEQVMFQPGHIVR NPGDLVR NTAPYSPNSLNK PAQIVR PIEQQFLINAIQFETSHLSKPLGFLGNAANVIQAAGAHK PVTGALGR PVVPIHNNNRSTLYPPGR TFADHWSQPR TPVFVR
263	KDB11386.1	78 kDa glucose-regulated protein homolog	111.95	72.66	25.4	AEEAEFHDEL ISEEDKETILEAVK ITPSYVAFTDEER IVNEPTAAAIAYGLDK KTEVDDIVLVGGSTR LSNVAYPITSK MYGSGEAGGEK NQVNDSEGLGGK QESITITNDK SQIFSTAADNQPVVLIQVYEGER VEILVNDQGNR VQALIEEYFGGK
264	KDB11321.1	Histidine biosynthesis trifunctional protein	49.606	92.585	9	AIDTSFENIR APFSPDLMR ILGPGNQFVTAAK IVTPIIDDVR LVFASPPR TMQEAMR VEELEAHR VTEAELLSTLQR

265	KDB11222.1	Ketol-acid reductoisomerase, mitochondrial	13.912	28.541	7.5	EVYSDLYGER YEAEMEEIR
266	KDB11157.1	Triosephosphate isomerase	130.41	27.125	32.8	DSNINWAILGHSER FFVGGNFK IVIAYEPIWAIGTGK PAFIDIINCR QPDIDGFLVGGASLK QPDIDGFLVGGASLKPAFIDIINCR TVEVVTR VATTEQAQEVHK
267	KDB17876.1	Cell wall protein phiA	13.278	19.843	10.8	DQLVFGNSK VISTENQAESK
268	KDB15283.1	1,3-beta-glucanosyltransferase gel1	125.72	49.177	28	AEPGPSYNAK DLGVNTIR FKDLGVNTIR GIDYQPGGSSANEDPLGDTDVCLR GPGLDLDGK MQTAMYMNCGSDDAR NYMNSR RVPVGYSAADVSK SDFFAFNDYSFCNSNFK TVTETDEYANFK VPVGYSAADVSK VYAVDNSLNHDK
269	KDB13933.1	1,3-beta-glucanosyltransferase gel2	99.116	51.832	22.3	AAWATIDWK DAALMQVMGVNAIR FQIVGVAYQPGGSAGFSK LIDSGISPK NVLFDTWNYMQCSDDGK QIPVGYSAADVR TSPDPLSQPDACLR VVPLADDEFNMAGK
270	KDB12613.1	Probable glucan endo-1,3-beta-glucosidase eglC	86.693	41.015	42.9	LFQAAIDNTK LYTMIQAGSDSDPISAIPAAIK NSPDPGASPDTLVSYIK PLYDLSCDGYK PVIDAIDFVGMDAYPYFENQKPNGLENAAK SATGDLPIFIAETGWPVSGPTENSAVPGIDAAQTYWR TAAGLEGTNGGFTSAR TSLLLGLWASAGQDSFNK VQSDFEAEFK
271	KDB18502.1	Disintegrin and metalloproteinase domain-containing protein B	14.77	84.778	2.5	FAPSGAVCR NVETSADIGSR
272	KDB17801.1	Tripeptidyl-peptidase sed1	55.956	66.437	16.2	AEEVAVFDPK ELYDIPLGTK GTFNPQGR GVTVLVAAGDR GWDPVSGLGSVK HNFYSGGGFSNINAR PAYQDQAVADYLASHDPHYPK RPIGFLNQILYR
273	KDB17179.1	Peroxiredoxin Asp f3	92.429	20.056	56.5	AGDSFPDGVK ANGLRDDFILFASDDAGFSK DDFILFASDDAGFSK GIEVSGAEAVLASL GVEQVVVIAYNDAWVMSAWGK SIGWDMGER VVLVSVPGAFTPTCQASHLPSYVAK YAVVVDHGK
274	KDB16140.1	Probable beta-glucosidase M	58.821	91.332	13.2	DLSIWDTTAQK ESDYGALLGPSKPEGR GAAMGGEYR HYIANEQESHR LDTSCSGNIAPNK SADSLSPGPGDIYINLWR TLHEVYLWPFQDGVK TVELPLTR TVVVLHNAGPR
275	KDB16044.1	Secreted beta-glucosidase sun1	61.159	47.122	20.1	DGYLELTR DGYLELTRPSVK GLDAEFPSGK IVITNPIQDK SQWSSAQGSTK TDYPGTENMVIPAAAEPHSK VLNPEEAAAGLK YYQWDGKPTSAQYYINK
276	KDB15368.1	Probable glycosidase crf1	166.2	22.537	33.8	DKYPQSPMQVK DSPANGGVK EWAGGYTDFSQAPFNAYYK EYIYGDNSGSWQSIK LGTWCAGGK SISIVDYAGK TLSADAVK YPQSPMQVK
277	KDB15085.1	Endo-chitosanase	6.348	59.029	1.8	DYGIVYLQGR
278	KDB14801.1	Calnexin homolog	61.936	62.712	20.7	ALHQEEFSNTTPYVIMFGPDK APFLEQFTDDWEK APFLEQFTDDWEKR DILFDNIYIGHSVADAEK FRDDPVLYVK IKNPDFFEDK NPAAHHAISAK VLAEADQPK VPDLPSFTPTTLK WSAPLIDNPAYK
279	KDB14282.1	Tripeptidyl-peptidase sed4	36.114	63.294	7.4	FGIAGFLEQWGSHADLDQSFR GVSVLSSSGDGGAK TWRPDLAK
280	KDB13746.1	GPI anchored CFEM domain protein C	14.635	15.347	15.3	EFLSGISCCLEHK TTDIACICANK
281	KDB13473.1	Dipeptidyl-peptidase 5	200.32	76.936	36.7	DDIWYPGSMGYDIHALVTR DPGLNPAR ELPTPIYEEATVSNAR EVLGFINK

						IESSLYSVLDPVSK LLGAGPSLLITSK LPISEGLAMFNVLQAR LQGYSVAPTFSSDGQK LTPEVLLSAPR LVVFPDENHWILKPENSLVWHK NIQYESDK NIQYESDKTHLLLVPNVDDVSK PDWITWSHDDKELYVGAEK PSDFDPSK SEPHMIHYLAGSISNPK SLGLNK SVTEVSSSSR TDLYYVPLK THLLLVPNVDDVSK TYTSLFVR VAVCFSGLTTPK
282	KDB13037.1	Nucleoside diphosphate kinase	87.18	22.125	51.5	AHLEAHYADLK EGDVVSYK GDYAIDVGR GLIGPIISR GLVEYMASGPICAMVWEGR LVSPGK NVCHGSDSVENAK QSQFSWIYEK QSQFSWIYEKE TILGATNPLASAPGTIR
283	KDB12970.1	Aspartic-type endopeptidase ctsD	28.639	55.239	6.8	ASDGTNEGEIR LHAVVPGSGSDDGLTFK NVYAVFDR
284	KDB12705.1	Vacuolar protease A	73.194	42.57	19.2	EPGLAFAFGR FDGILGLGYDTISVNR GFGGQYTIDCDK IQYIPLR IVPPFYNMVNQK KQDFAEATK QDFAEATK RYYSVYDLGK YYSVYDLGK
285	KDB12546.1	Probable glycosidase crf2	146.4	48.458	19.1	DWVAQGEPALLDGNVLLTMPK FTTLDSIVDISSYYGDASK GTVDWAGGPIDWNAPDIK IEWLIDGQVGR KDWVAQGEPALLDGNVLLTMPK VQLSIWPGGAATNAK WTPDKIEWLIDGQVGR
286	KDB12509.1	Mannose-6-phosphate isomerase	11.394	57.256	4.1	ALVGEEAASELAK SVLYGDGAK
287	KDB12369.1	Tripeptidyl-peptidase sed2	19.801	64.754	7.7	GVSVVAASGDGGASGTGGNTQCLGPGGR HYLAELAGK PAWQDTQVR
288	KDB12341.1	Phosphoglucomutase	123.19	59.823	41.8	AFVSPGDSLAIIAHHAK ATGGILLTASHNPGGPK DFWTQYGR DIFDFGVIK EFFTSHPDFK GSVLVIGGDGR HIPFGAASDGDGDR IFETSK LSGTGSSGATIR LVADSSFVGSK LVIGQNGILSTPAASHVIR LYIEQHSSDPSTYDMDAQDFLK SMPTSGAVDLVAK TVQFQPFQDQKPGTSGLR VLFDGLHGVTGPYGK YDYEDVDSDGANK YNLANGGPAPESVTNK
289	KDB11271.1	Probable aspartic-type endopeptidase opsB	61.278	58.14	14.2	AADPEALGLPSR EMVVDALPALIR FDFLFDGVTVAVPMR FSGGLAVLPLQPLPAR FYLLGDTFLR GESTPPLVDCAYAGPK
290	KDB15606.1	Endochitinase B1	323.31	85.814	36.8	ADPSPQGEDLTTSNAATCVK APGTNVYGNVK DRNEAVQMADLLR EALDDLAK FILSYASPAGPEHYK FTHLSYAFSNIK GAGLEGSLGSAGIWR ILLQNAIYGR KNPAATEYETQSGIEYYINQGK LAGTSWWEVSQDR LREALDDLAK LVEDLGFDGLDVDYEYVK MNFSPMLASR NEAVQMADLLR NPAATEYETQSGIEYYINQGK NSGFMGNVFLDK QLYLLK TAEYLISYDTAEIAK TDDLSLVK TETGEVVLSDEWADLQYAYPGDNTK TLGYYGNWDIYAAK TTIDEYGGLGALEK TTLSIGGWSYR YFVTDIPAER
291	KDB12252.1	Probable beta-glucosidase I	90.197	91.022	23.7	ADLHVEQVLQK AFVDDELVVDNATK EIYALPFQLAVR GFESLGEDPFLAGLGAAALVR IGNESIVLLK LGLSPEYTVGAYTHR LPGLLDQLISR LQDVPSFLNYR TLVVGPNAK TPVDVLFLSK VATYHGGGSAALPAYYAVTPYDGISGK VELEAGETK VSGLGIQEFGPEEEANTPETAALLR WTVYNQPPGTPDR

292	KDB17851.1	Cyanate hydratase	132.11	17.966	71.3	ASPEDVDKLSSLLGVPVAELAEQLTAFPNR FGDGIMSGVCFK GTSVPMPPTEPLIYR LSSLLGVPVAELAEQLTAFPNR LYEVVQNYGYAYK NLTFAQIAQHLGR SEVACAALFYGQAR TDVDKEVDETGAAWAVITMK
293	KDB16255.1	40S ribosomal protein S1	25.68	61.918	6.6	IVEVSLADLQK LIPEVIGR MTDIIQR TTYAASSQIR
294	KDB15846.1	Methylthioribose-1-phosphate isomerase	24.698	41.992	11.9	IGTYQLAVLAK LPHEFHYDDVSTR NIAAVIVGADR QEAFDSIATMR
295	KDB14469.1	Vacuolar protein sorting/targeting protein 10	38.394	170.33	4	AVASLDGK ESLSFFNQVQR IIYTIDR IVVTQITFDDGR LHLHSVTELDNVGR RVDSVPEGR
296	KDB14097.1	NAD(P)H-hydrate epimerase	12.587	18.877	16	DPFPDVIR LPITSVDAPSSWNIEHGPPR
297	KDB13462.1	ATP-dependent (S)-NAD(P)H-hydrate dehydratase	24.968	30.288	12.8	DPLMLETVSR LDVLVVGPGLGR MPVVLDADALQLVQR
298	KDB13454.1	Carboxypeptidase Y homolog A	54.651	60.972	15.7	AGHMTPFDQPEASLDFLNR GLDNSLAR LEWSGHEGFSK NFLLQGDWMK QDFHIAGESYAGHYIPVFTSEILSHK SHGNLSFLQIYR
299	KDB12825.1	S-methyl-5'-thioadenosine phosphorylase	77.455	33.716	31.9	GTVVVMEGPQFSSR GVVGHVGFADPFDAGLAAVVK HGLHHQFAPHEVPNR HWEGYAQGGLK LVGAVLDQLSQIEHGDLVMAK SWGGSVINMSTLPEAK
300	KDB11677.1	Enolase-phosphatase E1	28.335	28.109	19.4	AALLAGLR LAFESHVHDLVAR WLFLSDNPLEVR YLDDHWNTIGFAPYQK
301	KDB11786.1	2-methylcitrate synthase, mitochondrial	32.692	51.814	14.8	DAIPASYTEQDVHDYLWTTLNSGR DPVFQLVEK ENENFQDLLR IYQNAYR VENTLGGMR
302	KDB12056.1	Carboxylesterase A	59.724	64.52	19.4	AAAIAVISLPAR ALAQDLDAAMR DMVEMVDR FLQDYVDIPGR HYELVSFDPR LSVPLDWR PLADCFPGGTLAR VPDTDAAFAGPVLANPGGPGDSGVELLR YAVAHGLALVDGFAK
303	KDB17750.1	GTP cyclohydrolase 1 type 2 homolog	19.498	11.935	34.8	AAVAALK AAVFNAGAGR PGGTANPHFGTPGVLEQTPEVR
304	KDB15317.1	Gastricsin	60.626	40.96	24.7	EDTTPNGGLLTLGDSR FGMVVNGK GPMVTVPIVK LTLTGDELAR NFYTVWDFGSHDPAAYNPTLSFGK NGQFDMWR STTPGMIITTSGSNTPGR
305	KDB14851.1	Pyroglutamyl-peptidase 1	12.147	29.905	8	EQYPVNPSWEIAR ILVHPEPIR
306	KDB14567.1	WSC domain-containing protein 1	67.941	64.555	15.6	AFPVGLSIVSGDPTLR EFMSNLDPSK ENMVFPSDAGNGK GEGVGFPDVDCDGFASPLR GTAGMDQCPEIK NECTIEAPVNEK VGGLLDALPGNNR
307	KDB12263.1	Guanine deaminase	11.398	56.491	3	DPEGALVR FVMTGDDR
308	KDB12151.1	Aspartate aminotransferase, mitochondrial	32.746	47.439	12.6	IATEILSSPK ISVAGITTGNVGR LAYGANSPVLDR NMGLYGER PLYSNPPIHGAR
309	KDB14684.1	Probable phosphoketolase	52.506	93.858	11.3	AHQVPITDANTNAEHMK DIIAQNRFVNVVDLFK GNIDTPLELAIR GSPLSASDLSLYNDYFK INNPTILAR QTLLDEQISAR SNNYVNVIVADK
310	KDB18006.1	Uncharacterized protein MJ0644	6.161	31.848	3.3	LHQVLELLPR
311	KDB16271.1	Leucine aminopeptidase 1	29.224	43.177	16.7	AMLQQDMTGYVR GTLDAGLPESVGVVTDYVHPGLTR HIHTTSDLLEYLSFDHMLEHAK VHVLAQR
312	KDB11556.1	Probable Xaa-Pro aminopeptidase P	43.755	68.013	16.8	AHLENSNIQVKPYDVFFADAEK ALGGDGSVEEIR DLLGPDEK GLISLDTAVFPK PSRPQLPVTVLEDNFAGK TVAVDSTLIPGLAAK YFSQAAQELDANWTLLK
313	KDB11473.1	Probable Xaa-Pro aminopeptidase pepP	109.02	54.168	36	AAATLHYTDNNAPLSGK DFETVKPAIEASR DIYQIVLR EISEVEAAAAR ETTELIK

						GCQNLTTAIK GDAQEILDAR GGVIWDHLHLR LEDNVYVTEKMQEDNDSPEHFR SAPEMPYHPIVASGR VAATEQELYATFLER VFKDEYEVAMIR VVDLIR YIDANVLEK YWDVGGVR
314	KDB18052.1	Tubulin beta chain	56.871	82.202	9	AVLVDLEPGTMDAVR EVEDQMR GHYTEGAELVDNVLDVVR IREEFPDR NMMAASDFR VGEQFTAMFR
315	KDB17683.1	Cuticle-degrading protease	26.108	39.923	15.8	GFATSVDEAGLK LHQANAPWGLAR SNWGPFVDIHGPAVDVQSTLPGGR SQTVNTAAASLVR
316	KDB17088.1	Orotate phosphoribosyltransferase	8.3577	25.247	6	ILIVDDVITAGLAK
317	KDB16790.1	Beta-glucosidase cel3A	18.748	93.415	5.6	GVNVALGPSAGPLGR GYAGDNTCAGNTGSVPR RPYFGVEAASSNVDDK
318	KDB14017.1	6,7-dimethyl-8-ribityllumazine synthase	60.476	20.569	45.5	GETMHFEYISDAVSHGLMR GHNHGEDWGLAAVEMGVK GPTPQQHDGSGLR IAIVHAR NIVVQSCPGSWELPFAVQR WNDSIIAPLVSGAK
319	KDB13903.1	40S ribosomal protein S0	17.334	30.503	12.9	ADGVNIINIGK FAAHTGASSIAGR FTPGSFTNYITR
320	KDB11366.1	Saccharopine dehydrogenase [NADP(+), L-glutamate-forming]	104.63	51.743	26.7	DLVFLQHK DSTGYSER ENDLLSAIASK GVIAPMTTK GVLLALK HVVTTSYVSPSMLELHDEAK MSFEEGER QLLGAASSR QVLDGTISER VLVDMGFLR VLVDMGFLRDDEQDYLK WLGILSDAK YNIPEAHTIIR
321	KDB11273.1	Molybdopterin synthase catalytic subunit	12.196	18.355	18.3	DPLNAQAVMDR LGTVPVGQESILVAVSSPHR
322	KDB11027.1	Subtilisin-like proteinase Spm1	125.83	57.097	35.6	AYFSNYGK CTDIFAPGLNILSTWIGSK DVLSDIPNDTPNK ETLIHVSTR GLSFASYNK GSVANMSLGGGK GVDAYVIDTGTNVDHVDFEGR GVEFAATR IVAAGGYEGTPQASELEK QAPWGLAR SNGAGSMSDVVK THALDAAVNAAVK VGIHFAVAAGNDNADACNYSPAAAEHPVTVGASAFDDSR
323	KDB12487.1	Glutaminyl-peptide cyclotransferase-like protein	16.284	40.476	6.5	LLAPILIPR SIPSPGSDFDIHTGK
324	KDB13696.1	Uracil phosphoribosyltransferase	99.436	23.542	39.7	AAGEWPEGTELWIAGIDR EGMLRPGLGDIGDR EPSTLDPVEYYNNLPHR PGLGDIGDR SLVNDISLILATEALASNLTAVDGSK VLVLSVVGAEEGVK
325	KDB18748.1	Uracil catabolism protein 4	40.797	56.078	15.7	DYDAPFTSIPGHGR EGFFSGNPDNK ELAGNEMTLPQLLEAGSWK MHFAGQELLTGLPEYR PGNELAGLQGR YQHFGVGGR
326	KDB12351.1	Probable acid phosphatase	37.85	50.303	15.6	APLNIATVMNLLDYR DTVEVPDGLHPPR FLEPLLTNR LAIIYFENQNYDK RPILQAVK YTATDPASVAK
327	KDB11479.1	High-affinity glucose transporter	12.472	61.464	3.2	AEHGDVEAR SVLALVHGK
328	KDB13558.1	4-hydroxy-2-oxo-heptane-1,7-dioate aldolase	9.4188	30.448	4.6	SAQEATELVQAAK
329	KDB15065.1	Phosphoglycerate kinase	150.27	44.532	58.1	AHSSMVGVNLPQK ANDGDVVLLENLR ELPGVAALSSK FHLEEEGSSK GIPDGEMGLDCGEK GLTALGDVYINDAFGTAHR IQLIDNLLDK IVGAVPTIK IVIIGGGDTATVAAK LSHVSTGGGASLELLEGK LSITDVDVK SVILMSHLGR TILWNGPAGVFEFDSFAK

						VDFNVPLDENKK VGYATDSK VLDDMSIGNSLFDEAGSK VVLPVDFVTGDK YALDHGAK YSLQPVVPELEHLLGK
330	KDB13976.1	Alpha-galactosidase 2	144.05	82.398	29.6	ELPDLGR FDPGILHYFPQIWTSDDTDAVQR FHVDGATGDLIGDHFGGPLR GIHETPSPQTDHEYMLGLYK GNAIEADIGAAPGWVNMIGR HPDWALHAGDYPR INPIVVK LFEAHINDHWPVVR LFVLDDGWFGIK LHGLYR LPEESNYPAALFISRLQGLDPSANYR QSAGHTISR VDDGQVVSGATLMNK VGDDAGLGDWVANPQR VYESLTER YDAVVR
331	KDB12601.1	Pyruvate kinase	170.47	58.925	41.6	AEISDVGNAVTDGADCVMLSGETAK AVLGEEGK DAGLNVVR EILEETDGVMVAR GDLGIEIPAAEVFAAQK GGMGNTNTLR GVNLPNTDVDLPALSEK GVYPFLFPEAK IAESCAMAAVR KGVNLPNTDVDLPALSEK MNFSHGSYDYHQSVIDNTR NNVDMVFASFIR PDFNQVNWQEDVDKR QGLNNFR QIQIIAK SLSTMAATQQDHLNTGGR SSIICTIGPK TNSVEAINK VIEPGR
332	KDB12548.1	D/L-glyceraldehyde reductase	13.846	36.566	6.1	ALADVAGLK TADGSEVELNR
333	KDB12442.1	NADP-dependent mannitol dehydrogenase	19.46	28.348	10.9	LNVVDYNEVEK SMIPLGR VVVVTGASGPR
334	KDB11955.1	Acetylxylan esterase	82.302	32.558	24.1	ATVPAPEPGDSAQGCAK GLYLISAR GTGEAAGIGAAGTIIGR IALMGYSQGAQLTLDTLCGSDEK SLADLITEHVK
335	KDB11787.1	2-methylisocitrate lyase, mitochondrial	38.252	65.801	13.5	AYVDLVQR DHEFILGVTDPSIPPLAETMQAMEAK LVTFDEAAVAHMDR RPYSAADVVTK TPVHFDWDVPR VLVPTGEHINR
336	KDB12663.1	cAMP-dependent protein kinase regulatory subunit	12.309	42.473	11.3	AIEGNFLFSHLDDEQTAQILGALVEKPIPAK GAIQPGPEGLGDK
337	KDB13525.1	Protein disulfide-isomerase	178.7	54.655	50.4	AFGAHAGNLNLK ASSETFTSVAEK DNYLFGSVSDAAVAEAEGVK EEATEEAAPPAGAK EGDADEEAHDEL FADDFASGK FDAEEIEQFTK FPAFAIHETAK FPFDQDSK GLDNISPYNGQR GSPVTYSGSR INFATIDAK LPSIVLYK QDGPVTVVVAK QSLPAVSSLTK SEPVPEK TDKFPAFAIHETAK TIQDLVQFVK VDATANDVPDDISGFPTIK VDCTEETSLCSK VVLIGYIAADDK YEQLGEAYAK YQVEGYPTLK
338	KDB15880.1	Heat shock 70 kDa protein 12A	6.4088	82.874	1.4	EVVTLTSDLSR
339	KDB15235.1	Ras-related protein Rab-6A	7.472	17.162	7.2	LQLWDTAGQER
340	KDB12059.1	Cytochrome P450 1B1	27.258	91.621	3.9	DAFLEPGPR DAGFTWGQQALPALR LPGVEDEK
341	KDB11981.1	Alpha-N-acetylglucosaminidase	11.75	92.042	2.9	LLDILSTLDR YALGSSLAPPELHR
342	KDB11426.1	2-amino-3-carboxymuconate-6-semialdehyde decarboxylase	22.188	41.173	3.5	LILLESELADLDR
343	KDB18701.1	Vacuolar protein 8	14.162	71.101	3.4	AEDIIEHIR EAVADLLQYLENR
344	KDB18383.1	FK506-binding protein 1	37.741	12.081	43.4	ATLDISADSGYGAR EGTGETPVK IGVGQVIQGWDDGVTQMR QFDSSVGR
345	KDB17573.1	Tubulin alpha chain	35.945	50.012	17.4	EDLAALER ELIEGVLDK GDVVPNDTHAAVASLK KAQDPDQGFSTFFSETGQGK

						NLFHPEMMITGK SIQFVDWCPTGFK
346	KDB16999.1	Multicopper oxidase GIP1	46.11	65.743	11.3	AALDPK FVAAAITAGER LMVLSDWSR QVDFSIDEHPMWIYEVDGNYVQPHK SVAEAIAAEPESFNLK
347	KDB16995.1	Dehydratase aurZ	20.222	15.848	25.7	KPGISTEDYR NYMVNVHAPLVAGLMEK QTVGPDHEK
348	KDB16572.1	FK506-binding protein 2	68.279	18.827	45.3	FDASYDR GEPLTFTLGTGQVIK GVGPIPAGSVLVFETELVGIAGVPSPDEAK IAVADTDGGSQEHEEL KFDASYDR TLTIPPELGYGDR
349	KDB15436.1	Translationally-controlled tumor protein homolog	23.95	18.133	21.3	EDGTTPYVIVWK GAQTYVK GYLTYLK VNNIVHSFR
350	KDB14653.1	Cytochrome c	11.307	13.747	14	IGPALYGLFGR MAFGGLK
351	KDB13523.1	Cytochrome c peroxidase, mitochondrial	12.352	40.356	10	FAPESDHGANAGLLAAR MGLNDQEIVALSGAHALGR
352	KDB12004.1	Peptidyl-prolyl cis-trans isomerase B	11.402	29.099	6.7	ALATGEK DFMIQGGDFTK
353	KDB11307.1	Peptidyl-prolyl cis-trans isomerase H	11.328	22.791	7.2	TAENFR VVDGMDVVR
354	KDB11203.1	Peptidyl-prolyl cis-trans isomerase D	34.979	39.949	18.1	AGNLQVAVAK AHELAPSDGMVAK FEDEAFPLK SKDEENALDDLER TVENFR YLNEDPDLDNEPATTK
355	KDB13308.1	Aspartate aminotransferase, cytoplasmic	126.98	47.591	45.8	AAIQAAPDR AGCFHAVTSPGPDATSTISR DDDAKPWVLPVVK ELAVIIAQK HFPFFDCAYQGFASGNLAQDASAIR LESLGTPGTWNHITDQIGMFSFTGLSESQVER NFGLYGER NIHHVADAIDK NNPELNHEYAPIAGVPDFTSK SEISNPPLYGAR TVYLSNPTWANHK VADYPYFSK VDLGIGAYR VSMAGLNTK
356	KDB13272.1	D-amino-acid oxidase	11.248	28.836	7.5	GQLVLVR LGGVQDETMAPSR
357	KDB16028.1	Glutamine synthetase	12.094	37.588	5	DIVDSHYK EYGEDNDQR
358	KDB15137.1	Bifunctional cytokinin biosynthesis protein	6.5419	25.43	5.5	DNQFVPSESMYGR
359	KDB18136.1	Transaldolase	135.27	30.37	49.6	AAEILQR ALHIIDLYK EEDPGVASVK EYSKEEDPGVASVK FAADAVTLK FDFNEDQMAVEK FSFDTEESVDK GGDVDSQVDHALDR IASTWEGIK KGGDVDSQVDHALDR KLDAAAAASANLER KSYINDEPLFRLDAAAAASANLER LLVEFGK SYINDEPLFR TIVMGASFR VSTEVDAR
360	KDB12414.1	Aspartate kinase FUB3	43.566	19.594	27.4	EFLVIIPDKPGATAK EQVLELLK NITPLTESGVVK QDVYSASGVWDIEK
361	KDB15461.1	Thioredoxin-like protein	21.321	13.02	28	AAPVHHINSPEELR AIAPVFEK GADAAALGAAAEK
362	KDB15731.1	Disulfide-bond oxidoreductase YfcG	27.941	77.909	5.8	ASIALEELGLQYK IPAITDTLPGGQK VFESGAILEYLVDR
363	KDB19077.1	Xanthine dehydrogenase	7.7062	86.2	4	NVGTPAGNLVTASPISDLNPALWAADAVLVTR
364	KDB18587.1	Homogentisate 1,2-dioxygenase	80.934	51.425	31.6	AGAGGFVPGGASLHNVMSGHGPDAESHEAAR DFLAPVAHFTQDFGPTAHEGSGR DHDFVSGLK FNNALFR ILPSCAHPPYQPSPEDAAR LSGTAFTAPR LVAGAGDATQK MHYIPNQLR NTMSEFMGLIR TELGWLLVR VGLASGPAR
365	KDB18447.1	Heat shock protein 60	196.73	77.434	35.8	AATSGFHEVEANDFEELEYETVAK ASLLAGVDTLAK AVATTLGPK DKFENLGAR

						FVDALNATR GEYVDMIEAGILDPLK GFVSPYFITDTK GIQAAVDSVVEYLHK GQLQVAAVK GVAADATTSEYEK GVVGPGCR LTDEYAADFNK NVLIESSFGSPK TIEVEDASEEQDWESFR TMQDELEVTEGMR TNDVAGDGTTTATVLAR VANFDQQLGVNIVK VEFENPLILLSEK VGGSSEVEVGEK YAVYDFEPEVPAGR
366	KDB18121.1	40S ribosomal protein S19	11.991	16.797	13.9	IGVLEQDEEK LPIPGWVDTVK
367	KDB18073.1	Alanine--tRNA ligase	23.19	103.47	4.4	EVLGDDVNQK IAAAVVADQK LDALDDMPFGAR TALGILLDR
368	KDB18053.1	3'(2'),5'-bisphosphate nucleotidase	7.3377	38.603	3.3	LAGDVGSNQTDR
369	KDB17905.1	Cell division control protein 48	46.842	92.92	7.9	EMVELPLR GDTVLVR KTPVSSDVDLGYIASK LDSLIYVPLPDEVGR QGDLFIVR VVSQLLTLMDGMK
370	KDB17879.1	4-aminobutyrate aminotransferase	6.4587	56.409	2.3	GQGTFIAFDNPR
371	KDB17080.1	Heat shock 70 kDa protein	87.002	76.586	14.3	ADSMLNDTER DAGQIAGLNVLR EKTDDLQMASLNLFDK NAIEAANR NESSSSEQQPEGENK NESSSSEQQPEGENKGENKP TDDLQMASLNLFDK TIEPVR TLNEHADKLDK TSGIDLSSDR YAEADKER
372	KDB16513.1	Uricase	6.5703	34.514	3.9	DEYTTLSDTWDR
373	KDB14945.1	Probable 4-hydroxy-2-oxoglutarate aldolase, mitochondrial	85.628	36.482	30.2	ALLQAVR DGFPDYPIVAGTATHSIEETVEQLVDAK EAGSQWGMVLVPGYNAAVTPQDGIVR GFGDADGSR GLVLFGSTGESVHVHPR HGIVGVK RQPTEAEFR
374	KDB14792.1	Hexokinase	94.359	54.249	23	EILDWPAK IFAGQDISLLR KSEFDIIQSK LAELIGTR LEDLFTVDTALLK LTALINDTTGTLIASAYTDTK LVLVDLHDNR RLEDLFTVDTALLK SEFDIIQSK TCHVGADGSVFNK TEFDVIIDR VCEITLTDR
375	KDB14608.1	Homocysteine synthase	45.866	34.177	23.3	AVYVESIGNPR FVDVDKPEDVAAAIDDK GGGAAGSQVVDGFK HAQNALALAK LISNLANVGDAK YNVPDLEAIAR
376	KDB14292.1	Carbonic anhydrase	29.221	24.661	19.3	ATTDPGYFAK DLGLLNPWLR IDFQAMLQNIQK TAAVQQSYAR
377	KDB14255.1	Fumarylacetoacetase	43.332	46.535	21	AAGIPNQETLLPYLR ASSVVVSGTPVRFLHDGDTLTIR QHLQSVLSAR RPAVAIGDYALDLR VPVLAPCEK VVNHLPLAVGDYTDFYAGK
378	KDB12967.1	GTP-binding protein rhoA	11.631	21.986	11.8	EVFEHATR TNQKPVTPEAGEEIR
379	KDB12808.1	Nuclear transport factor 2	91.018	14.247	60	DAAGQYYVYNDIFR DNSMLTFETASSLGATSIAEK LVGLPFQK MENNFEDVAK QFIEFYYNTFDSDR QFIEFYYNTFDSDRK SLSSLYR
380	KDB12711.1	Histone H1	11.384	19.872	13.1	ASHATYQDMITDAIVNLK TAKPSAK
381	KDB12708.1	Acyl-CoA dehydrogenase apdG	30.829	60.167	7.4	FQDEHPGGK LQVGSLDTKPK TFTTSDVASHNK YHNEGILK
382	KDB11567.1	Lactobacillus shifted protein	27.965	31.144	6.1	GSYLITDQVVGALPEIR
383	KDB11543.1	Aconitate hydratase, mitochondrial	183.94	85.943	29.5	AAFTVTPGSEQIR ATVNVQVSPSSDR DALDHGLK EFLLKPPTGDGLPAR GHLDNISNNMLIGAINEANDEANK GYDPGNDTYQAPPK HLGGLAIVTR ILYSHLDDPHGQDIER KGEANSILSSYNR LNRPLTYAEKLQILQPFQPWDGK

						LTGELSGWTAPK MSENLAIVR MYDYLAATK PVTMVVHPK QGMLPLTFADPADYDK QNIWEENNFINYK VADILTVK YNIGFWR
384	KDB17420.1	Pyrimidodiazepine synthase	11.789	31.477	8.7	GLVPTLAVPVDGAGK PLLPSDAYR
385	KDB14973.1	Cytochrome b5	6.1747	21.666	4.5	IGTVAEAPK
386	KDB14891.1	Probable small nuclear ribonucleoprotein Sm D1	16.252	8.8449	40.7	GQDPVALDTMNIR YFILPDSLPLDTLLIDDAPKPR
387	KDB13922.1	Peptidyl-prolyl cis-trans isomerase	100.32	6.9388	40	HVVFGEVIEGMDIVK IESLGSQSGK KIESLGSQSGK
388	KDB11464.1	Probable chitinase 10	317.83	118.39	38.9	ATSNVVTSFK ATVTTDKDAAVK CSGNEIEIAAK CVWGECGATCPSGLVPVASGK DAPICTWK DKPMELTTGK EKDKPMELTTGK FGFCGTTEEFCGGK GSNGLCGATSGGR GYLGSK IGLLTDVVDTFTYR IVTFDGDQWVSYDDEETLR NAIQSMIDGVTAK NCGGLTPEKNLFLETSAFDSGCAPTHQQR NPFVSAQK NVLGIELGCNK QSDVNLYFQAHPGGSTIFKSCLYNGGFK SECNPGWGK SFTLSNPGCATAGCGFR SGHTTWVQWK SGVPEDFDNYVSFLK TFANSLLSLMDK TFSELAASSDHQK TGCEPGEVTVATDSFGCR TLASYVNWVTEHVFEK TLISLPIAK TSTDANFGACK VIGYFEGWAHTK VIMGMGFYGRVKPPTCSGGTSANK YGAQLVADQQK YGFDGVDIDWEYPVAEER
389	KDB13459.1	Small nuclear ribonucleoprotein-associated protein B	11.371	20.981	9.1	SMTGQMLAFDK TLGLIIVR
390	KDB13266.1	Probable short/branched chain specific acyl-CoA dehydrogenase	12.259	47.998	5.4	DMDEAEDMDPDVVR YAISLLNEGR
391	KDB12436.1	Putative protein disulfide-isomerase DDB_G0275025	59.076	49.284	20.2	ADVELIAVNAR EIIVDSVEK HYEGDFGAESVESWIDAIR QVGEPNPDPAPPKSTGATPEVIAITTIATK VAAVDCDDDANK VTASLSQINTK
392	KDB11789.1	Type-1 glutamine synthetase 2	36.08	53.431	13.6	DAPDPHAPWPDVAALSDLGR DMLAVPDLTTFR EIFGDDFVDHFGGTR LAGIDVDGILR LISAPTSK
393	KDB11706.1	Probable enoyl-CoA hydratase, mitochondrial	24.591	31.536	14.5	LGTIPGAGGSQR SFIESWSLLTTQIK SQDLALR VIVLTGSQR
394	KDB19144.1	Aldehyde dehydrogenase	141.63	65.109	31.1	DVDVAVAAAR ELGEAALANYTQNK IAFTGSTLVGR IFVQEGIYDK KFEVVNPTTEEVITSVCEATEK LADLVEK LGNQSNYGLAAAIHTR NLDLLAAVESLDNGKSGKEEGATVEVGGGR TAEQTPLSALVFATLVK VAGAAIASHMDVDK VGDPFAQDTFQGPQISQVQYDR VGPALATGNTIVMK
395	KDB13291.1	Acetyl-CoA acetyltransferase, mitochondrial	13.487	44.899	5.8	AFADEIVPVTVPGR AVAQQYGSGSR
396	KDB17900.1	Guanine nucleotide-binding protein subunit alpha	10.252	41.067	3.4	EIIFSNTVQSMR
397	KDB11418.1	Endothiapepsin	34.713	42.932	13.5	AAFVVFDGGK KTSISGIADTGTTLLLLPDSIVK NEHFRPDGPAALAR PLPLDLAR

						TSISGIADTGTTLLLLPDSIVK
398	KDB17766.1	4-formylbenzenesulfonate dehydrogenase TsaC1/TsaC2	10.7	26.781	5.2	VAVVTGAAGGFGK
399	KDB12729.1	Ubiquitin-conjugating enzyme E2-16 kDa	12.744	16.492	4.8	ELTDLGR
400	KDB18161.1	Putative branched-chain-amino-acid aminotransferase TOXF	136.41	41.595	42.1	AADEFGWR ALVLDEFDR CVIDSVTSDSVQR EVNGHVESTYSQTTGEWSPLR FVADPFMR GAAPGGLAAGPR IHGMAPALNYGQQAYEGLK QEGYGITLHLDSAK SAQVVSMPEVPVELFMR TPGDGGICIFR VGGNYAPVMR YAELPSFSEVFAAGTAAALVPIR
401	KDB15611.1	Beta-hexosaminidase 1	213.44	68.328	40.5	AAALAELVWSGNK AAALAELVWSGNKDPK AYPLFMSDR DVIMQSWNNGVGNVAK DWFAADK EQIDGLALSKETYSSQDVLEVVSYAR EYLVANGVAAAPLMPK GVMVDTGR HAGEPLTSVDVK HVIGAASPLWSEQVDDTVISGK IKEQIDGLALSK IVGPLQLR LVMWEDVVLSPDAR NYISVGK QIDKDIVTCQDSWWSNDDWPLHTAVEPNPGQLDVLNPK TTSLTQR TYFDLNQHWIDK TYTVVQNVYAELSK VMPEIDMPGHSSSGWK
402	KDB18484.1	Ornithine carbamoyltransferase, mitochondrial	79.826	37.75	27	AFEGYQITNELAK AGETPSSLSASMSGR GYGIPEGMK HLMSISDVTPAEFVSLVR HPEEVADEVFYSPR LITSAATGVESPGK VGPHSDVAGLAK
403	KDB12586.1	Endochitinase 1	74.4	41.999	29.6	HHSGVAASLASIQTYR ILSIAVPGLSR IVEQVDFINVMTYDLLNR NQFNPGTKSDVFNSEDEHPDFPLFTSVTQVR TGAFSWHDAVPEELAPSFAR YFEDGSYGYLDPAER
404	KDB18458.1	Glyceraldehyde-3-phosphate dehydrogenase	78.777	31.304	37.9	FTIVEGLMTTVHSYTATQK GAAQNIIPSSTGAAK HDADGLTINGK LISWYDNEWGYSR VIISAPSADAPMYVMGVNEK VLDLIAYVSK VPTANVSVVDLTAR YDSSHGVFK
405	KDB18140.1	Superoxide dismutase [Cu-Zn]	83.545	16.104	73.9	GFHIHTFGDNTNGCTSAGPHFNPHGK HVGDLGNIETDGQGNAK LIGPHSVIGR PACGVIGISS THGAPSDEAR TVVVHAGTDDLGK VGGTVVFEQASESDPTTVTWDISGNDPNAK
406	KDB12002.1	Dipeptidase tcpJ	120.63	52.506	30	AESSPVMEDSIR DSILQLVR ELIHEMNR IAGGNVLR IDNDEWR LISPLGVEGLHQIGNHVANLR LKAESSPVMEDSIR NSVVMVNVSPDFISCR RFHELGVR VMEAFPHDFAPR VWHDVDAVSAR WNGVSPLGR YADAALLESPLR YPDLIAALLR
407	KDB12892.1	Sedoheptulose-1,7-bisphosphatase, chloroplastic	6.4758	34.627	3.4	GQGVYLSPVTR
408	KDB18882.1	Formate dehydrogenase	107.54	41.438	34.1	EDVAAALESGHLAGYGGDVWFPQPAER ELLYYDYQPLSPEK GDWDVAAVAK GSYLVNTAR HDYRPEDLIVHQGDYATK NFVPAHEQVER SILASYLSGK VVGTVAVGR WLQDQGHTLVTTSDK YADGVK
409	KDB18092.1	Ribosome-associated molecular chaperone SSB1	12.876	66.69	4.1	DAGAIAGLNVLR VEEIVTDPTLSLK
410	KDB14940.1	Nucleoporin GLE2	19.843	39.279	7.5	IYEIAQNGQSQGR QQGQAAATVSCQER
411	KDB12514.1	40S ribosomal protein S24	6.2226	15.537	6.6	DQVQVFGLR

412	KDB11247.1	Nucleoporin POM33	6.2686	39.539	4.1	VDSLVGAQGTPPVAR
413	KDB12938.1	Autophagy-related protein 2	10.876	234	0.3	VEVEVR
414	KDB17311.1	Pyridoxal 5'-phosphate synthase subunit PDX1	17.874	32.591	10.9	AALAEGGIIR AIVQATTHFR GGVIMDVTSAAQAR
415	KDB14295.1	3-ketoacyl-CoA thiolase A, peroxisomal	12.883	45.875	5.6	IMGIGPSVAIPK YVGSTVAGLAPR
416	KDB15884.1	Carnitine O-acetyltransferase, mitochondrial	74.301	68.444	22	AAAASAAAPVPPVGALTSENR AHQYWHGDGANR DLLLPTIQAPK DVWADAR HIVVIR LLQPGQEVPAIFK LPICMDSYR LPVPTLEETAK MSYYINEAAGEMR SRPTYESAATR VPHEVGGVQLNAAELESQFNR
417	KDB18441.1	NADPH-dependent conjugated polyketone reductase C1	12.615	33.015	6.3	LTEEEIAR YNVSESEIGLR
418	KDB14900.1	40S ribosomal protein S13	12.848	16.809	12.6	GATPSQIGVILR LILIESR
419	KDB18910.1	Mannan endo-1,6-alpha-mannosidase DCW1	116.53	53.966	16.7	AAAATCTGGASGHQCGMK DITWNR DLDVTSQISIR DYMPAEHADEEGNDDLHFWGAAVLSAAER IYDGAGIDSQCR SLLVPTSR
420	KDB15263.1	Elongation factor 3	11.9	117	3.4	AVFETPDILLLDEPTNHLDVK PHLIVLDEPTNYLDR
421	KDB17813.1	Adenosylhomocysteinase	167.66	48.817	52.8	ANAQSVQNIKPQVDR ASDEAFSK EIELAENEMPGLMATR ESLIDGIK FDNLYGCR GCYGVSEETTTGVHHLYR HIILLAEGR IGQIFVTTTGCR ILDEEVAR LHLDHVQAELSTLTK LLVPAINVNDSVTK LNLILDDGGDLTSLVHSK RATDVMVAGK VADISLAAFGR VAVVAGFGDVGK VLVTEIDPINALQAAMAGYQVTTMEK VQADYLGLAAEGPYK YAADQPLK YAEFAR
422	KDB16843.1	G2/mitotic-specific cyclin-4	10.88	71.036	3.9	TQSRQFEMVDLPADKATR VTTKVQR
423	KDB16026.1	Regulator of cytoskeleton and endocytosis RVS167	6.6478	48.047	2.3	AGDVIEIVSR
424	KDB13937.1	60S ribosomal protein L13	11.137	29.556	5.7	EAGIAR TIGISVDHR
425	KDB13264.1	Glycolipid 2-alpha-mannosyltransferase 1	19.15	48.27	7.2	NTDVWDIAR TYIDYFESLDR VIYGDSESYR
426	KDB10889.1	Malate dehydrogenase, cytoplasmic	66.064	34.386	30.6	AETFVAEIIGEKEPHK DADIIVIPAGIPR DDLFNINAGIVK LFGVTTLDIVR LNIPVIGGHSGETIVPLLSQAK PTVDIPSEK SLIETAADVAPK VFNAQR
427	KDB12863.1	Glucosamine 6-phosphate N-acetyltransferase	11.364	22.119	13.3	DQFEQQFR FIHSLGSVGHIEDIAVAK
428	KDB11318.1	UDP-N-acetylglucosamine pyrophosphorylase	11.658	48.241	3.9	FLESIPQWAHK ILLESR
429	KDB15724.1	Protein memo-1 homolog	6.3659	36.564	3.3	LLDEAAMDAVK
430	KDB14211.1; KDB14208.1	Sphingomyelin phosphodiesterase 2	137.18	79.073	32	ATSYDEQFCNGVVER DETLFDQYMSR DIGAYSTK DPNGQLAWLVK EAPPIASIVR EEICNLR ELDAAER ENVYIIGHMPMGTSDALPNGSNYFDQIVK FNFLLYQDK GIVADGDEAFISLGK HAYTTVNDYIDVVYGTAGNHEADPPNIFEPK KPLQVIQYSDIHIDPLYVEGSSTK NILGILK NPAGAFGDHNCDTPVTLEK VYDVDPETFAVIDAHTYIADMDAPGFQK
431	KDB14163.1	Non-hemolytic phospholipase C	117.88	71.517	26.8	ADGWVTSDTPYSMGYYK ANAEQDMNALVEAGYK DAAGEWITDPFDASNGK

						FGNTFYIQSALEK FIISPWTR GCSVVAIQSSK HIILFMQENR NIVQAVVNSPR QHMTNMVNAFDFDNPIYTLPIITAVR SFDHYFGTMAGVR TTPEYLQEAGISWRVPIGPGPR WILHTADGNR
432	KDB13450.1	40S ribosomal protein S18	6.5838	17.875	7.7	IVTIIQNPTQYK
433	KDB17526.1	Tubulin beta chain	46.703	42.25	23.7	EVEDQMR GHYTEGAELVDNVLDVVR IREEFPDR NMMAASDFR VGEQFTAMFR
434	KDB15909.1	[3-methyl-2-oxobutanoate dehydrogenase [lipoamide]] kinase, mitochondrial	22.266	78.6	4.8	DDATDEQVK GFSVTFDK QQAVEQGGVIGHEYTLFK
435	KDB13955.1	5'-nucleotidase	115.67	63.173	21.6	ETSSWFQVSR FDAMTLGNHEWDGGDEK GCVGGYAR GTTFLDPIPEIQNAIYEIR HGVAVIGATTETTPNIANVGK IVALTHLGYEVDQK LAQETEGLSLIIGGHSHSLLGDMDK SGHEVFVVTAYR
436	KDB11439.1	40S ribosomal protein S12	11.492	13.559	17.4	LALMHDGLAR SILLNYFQTSQ
437	KDB14729.1	Oxidoreductase OpS5	6.3929	67.044	1.8	LINAGSEGLQR
438	KDB14766.1	Xylosidase/arabinosidase	78.237	155.71	7.2	ASGGSWTR AVWAPAPVSR EITPDNYVEGPQMVK HADVLVASDFAWAR LGAVAADIR LGAVAADIRPGQR LHFNEDGTIQPVR LNPDMVSLGSFDDGATYK NDGYAVLVFR
439	KDB15204.1	Glutathione hydrolase-like YwrD proenzyme	23.42	68.994	6.1	GFPVSEGAAYLWQASEAR LDPQQALDAPR VLAPAVELGEK
440	KDB13158.1	Guanine deaminase	19.793	28.033	12.6	GLRPQTALLGGVLDGETDR YFAWQFDGAAACPR
441	KDB13075.1	Probable 3-hydroxybutyryl-CoA dehydrogenase	12.796	31.56	10.8	IAAATTSDPTDTSTSSR IASVSADAPGFLANR
442	KDB11788.1	Uncharacterized oxidoreductase YxbG	9.9155	29.771	6.8	AYVTPEGPAAAAPVNNASK
443	KDB11477.1	Minor extracellular protease vpr	36.322	93.965	7.4	AAAMIDAATGESFIK ADVVPITPSLSK IFGDAR LDSGAFAPPGEYK LTVPILVSEK MTTEHWGVR
444	KDB14267.1	Glucan endo-1,3-beta-glucosidase A1	94.782	31.741	32.9	DESTIQGWTSGR FGPSHQGAK NVQLSGGSTVQIVPWR QSITWFLDGHR SPGGICNEPSGIGSSVNIPNNDYHVWR WQDPFSGGSGR YVFTPDSGR
445	KDB14320.1	Probable mannosyl-oligosaccharide alpha-1,2-mannosidase 1B	130.7	58.983	36.7	AESYLLQPTGSPEAWPGLVGTFVSTEDGK FLDSQGGWNGGDDSFYEYLIK HAFPHDTLR HLGADAQK IDFTTTAQEDYGISLFETNIR MYVYDPKNQFVYNTECHPVR NSGATSNNIAEIGTLVLEWTR VEALLTQAQTLGDSLSVAFDTPSGIPDGVVFLNPAK VNDAYAELTDISK WVDAVESTIAHLVSHPTTR
446	KDB16468.1	Probable beta-glucosidase A	268.38	95.588	27.7	AFVSQMTLIEK DTYGYR EISNWDTVSQNWVVTEAPK FLAVIGEDAGQNPQGPNGCADR GVDVTLGPVSGPLGR HIDTFIYPWLYPTTSGK HNVGLMSPPR IAPGQSVTFR NDGVLPLK NLPLSATLG NPEGFGTDPYLQGR QASGDADYGQTAEQFLPPGATDGSPQPK QAVQDGSR QVPTSFSSWTR REISNWDTVSQNWVVTEAPK SPGAPIYEFGHGLSWTTFK TLHEVYAWPFADAIR TVWVGSSSR YESVLSNYEWDAAR
447	KDB14875.1	Pyruvate carboxylase	179.02	157.69	18	AAVENVSR AAVENVSRPK ADPSDSSQVGAPMSGVLVELR ALDTYWSQLR

						AYEHANDLLGDIVK DAHQSLLATR DAILADAVK FFLSRPEIGEEFNVELEK FLYEDPWDR FVQQYGDLSVLPTR GCLIMDTTWR ITTEDPAEQFRPDTGK LLAVGPLSENTGQR LVPNIPFQMLLR PGLFLEPVDFAK RPGLFLEPVDFAK TVDLLNIAK VDVSEPCQNGWR VFDALNDINQLEVGIK VVGDLAQFMVSNK YGAPVTECDVASHVMYPK YNLEYYLDLVDK
448	KDB14422.1	Acyltransferase LovD	72.627	44.295	24.6	AVLGGTGALNLR HAPADQEQGQR ILAPFGVDDAEFYPVTR ILQSLLANDGK KPLQLDDVLYLASASK LVDLNPDDPDATGR VGVDSETTK VTGQTLGQHVR
449	KDB13148.1	Carboxypeptidase cpdS	11.729	54.668	5	FFVDGSAIPEVK PSAQGPLPSVIER
450	KDB13704.1	Pyruvate decarboxylase	31.989	63.905	10	AIVDSSEVSDTK ELIAASQWHGITR GLSMIEVVMER QGYNDVAR TLMHHTLNDGEYQR
451	KDB18851.1	Probable aspartic-type endopeptidase opsB	31.207	80.141	8	AAYVVYDQENR DGSCVLGVVPDDKFPVLGDTFLR GSVVFGGLDTK RPIIPADK TEINVPYR
452	KDB18144.1	Acetamidase	35.933	119.9	6.3	ALDMTEAALR GPLAGIPVSLK GVAQMRFYAK LGALSAYR TLNDLFYFTR YTPGGSTGGEGALLAMGGR
453	KDB17213.1	Nuclease S1	36.547	33.573	18.9	AGPVIELQVAR IHVLWDGK MAAWLDQIAK SDDNDYLAK VASWADSVR WANQLAGEIR
454	KDB16341.1	Aspartyl aminopeptidase	168.55	53.555	54.4	AALDFVDFVNESPTPYHATQAAAK DLSIAGR DSSSLNQDPTIR DSWASTLQSGGK ESELFPIIGLAAAELNK FFTHYGTLEPR HEGEESTTYEQTLSR HHPAVLDVIAGELNVK IEKPLLR IPTLAIHLDR LAGVPLQLFVVR LASLPGGPDAASDGSYETVR LDNLGMTYCSVEGLISSVR NDSPCGSTIGPGLAAK QTNFDPNK
455	KDB14966.1	S-(hydroxymethyl)glutathione dehydrogenase	22.951	42.482	11.2	SFLVSADMAHAVHPNYAGK SVIGGLQDELIFSPR VSDIADFELVLYDTQK YESSHQPAMNK AAVAWEASK ELSIEDVEVAPPK IIVTDVNPAK SLSEINSAFELMK DFLAEFNTAQNLEGAPGDFNSVR DLSTKPLFDLTCPK EITALKK
456	KDB14252.1	Probable glucan endo-1,3-beta-glucosidase eglC	323.31	46.106	34.1	KDFLAEFNTAQNLEGAPGDFNSVR LYTNIQAYSQDDPIEAFEAAIETK NAGHLFDK NDAGIGNSPANIIK PLFDLTCPK PVWLCETGWPSSGPK TNLLLGVWASGTDNINK TPTFWYTLR WDDAVASVENAK YYWDEVGCR
457	KDB13610.1	3-phytase A	48.192	59.719	13.5	AQGIGYLQEMLAR FPDSIDGAVTR LDGLCELGNFIESQQDAYR LNNGILPLATIR VAYSPSQYGYDASNSSHK
458	KDB13042.1	tRNA (adenine(58)-N(1))-methyltransferase catalytic subunit trm61	13.174	75.988	2.8	FFIVPDMLR VSHLEQVYIR
459	KDB12781.1	1,4-alpha-glucan-branching enzyme	11.007	72.599	0.9	AEDVPR
460	KDB12419.1	Glutaminase A	323.31	79.777	55.4	ADSYGLLYNIYPDK AGHTTLQYDK AMTDLYDVATGDYPANLQFTAR ASDGMDAR DGIQFTGQAK DSVAAGGQDYATMTTLVLR DYDNAAR EDDGYLADNWDLLVK EVNGPNPILPGQWPR EYLAFWTQHGVNQK GIIALQAMSQVATK HLLEPLLINDR IGADTAVR IYDMQSDFYLAK LDLIPQR LDNTVDTEYR NPYLNVWLNK PVMGGLFAPLAL QIFGALAYTGAADDPYIFLK QSIPFSYLIVK SIFTFSVAGK SLDGASHSVQVYSDVSGEFASGDDNAVVK SVPSLWTAYLAEEDLAGFFYK

						TDWEMFAAAVAK TIFTIGVAQK VTLNVEFLSPVYPTDLK WETASASGVR WIGETPTWR WPVFAFAR YGAILDTR YHYPNDFAQHDLGR
461	KDB10987.1	Fructose-bisphosphate aldolase	91.415	43.254	36.5	DYLLTAVGNPDGEDKPNK DYLLTAVGNPDGEDKPNKK EDKPVFFVFHGGSGSSK GLSNTEQAASIAGAVAGATYIR GVTDVLSR LFAYAR LHPELLGK LLPWLDGMLDADEAYFK QFAVPAINVTSSSTVVASLEAAR SGVIVGDDVLK TEYLDAISHGVVK
462	KDB13987.1	Alpha-L-rhamnosidase rgxB	48.729	51.967	15.5	DGQGVAIGSMGQYDGK GLTNSAVR NVVGTVVK QPPPDADYLAR SQGNLPR TAMTTLAMDDVR VTVDGTDAVLDK
463	KDB16444.1	FAD-linked oxidoreductase azaL	43.559	55.849	17	AYVNYAYGNEGPK DAHLLAAPLITYRPDGPALDQR DLAGWTGISLSSPPCQK IGGGTLSK PFHDLGPIAVTPLSGTYR VSAVYDAANK
464	KDB16687.1	Glucose-6-phosphate 1-dehydrogenase	35.703	57.148	12.1	GGYFDEFGIIR NVIIGQYGR SIIPMEYPYGSR SLEPDWDESELFR VIVEKPFGNDLASFR
465	KDB14764.1	Copper amine oxidase 1	25.941	73.68	8	APHPLSQLSAEEFTR IDPAIDSYR LATGGLGDGLEPAPR SAEWVPELLDTPVR
466	KDB14941.1	Peptidase S41 family protein ustP	33.153	88.803	8.4	DYFYPGYDIFGNLAR IVQDGNSR LLDAIEPYLEWQSDAAYK QVFAPGQDGHFVFYPDLLSR YTTLPMER
467	KDB12463.1	Beta-cyclopiazonate dehydrogenase	39.743	55.038	17.5	EMLLQPLAR LEDALGILR NTPNAPSEYFLPHGDFNDAYVR VIGTAGLGDAEAR VLDLIRDDVLLESTVASSQR YGPVDPVGVPIGLLR
468	KDB16220.1	Acid phosphatase	66.016	54.598	24.2	APDGVDGATK AYQAITEQFYDQLAPIASR DLIPSIPPSMEHTTISR MGPFGAPNQQLQFLEADLASVDR MPADMTTPVQQR SSTGEVVDSSTLYK YGPADCSLTQQACSPSSAQTYPTSR
469	KDB18213.1	Probable mannosyl-oligosaccharide alpha-1,2-mannosidase 1B	232.81	57.012	49.2	AGSGFAILDDVTKPDGGTK DLLTTNR ENFMESFFLAETLK GTASGIGPELFR GYYDHAFR LADYALRPETIESLYYAYR MYLYDPDEFGHYK QPLYKPDAEK SQFGSWGGGTDSYYEYLIK TTFVFNTEAHPLR TVPAEYAESYK TVPAEYAESYKK TYAQLAQK VHFQGYGK WVAAAESSIK YAYLTFAGEGK YLGGLLAGYDLLTGPYK YLTEPQGLPESFPGLVGNELGIK YQDMVWSAFTTIR YVDFGLALAESYYETYK
470	KDB15626.1	Catalase-peroxidase	309.69	81.599	50.3	ALMTDSQDWWPADFGHYGGLFVR AVAEVYGSCDGSQR CPVQAQLNVAGGGTR DAGVDIK DFLANPDK DFVAAWDK DWEVNNPAQLSK DWWPETVK EDLLWQDPIPSVSHALIDDK EDLLWQDPIPSVSHALIDDKDISAIK EPEGANIEQQGLGWNSTYGTGK FADAFARFAPLNSWPDNASLDK GDATQEQTDIESVNYLQPLADGFR GPDTITSGLEVTWTK MLTTDLSLR NDLIFGSHPELR PGVLTNDFFVNLLDPTTEWR QEHLLVDR QTGEDVFGGFDRSPAGANQWVAK TDDLIIPDAYDANK TFGFAGGR THGAGPATNVGK THGAGPATNVGKEPEGANIEQQGLGWNSTYGTGK TLDFEGLK TLDFEGLKK
471	KDB11000.1	Metacaspase-1A	11.064	49.548	6.9	TVSIADLIVLAGVAGVEK VLDTNYDGSAHGVFTSR VLGALEDLQK VPFTPGR MGHIVDDEIHMR REDMVMLTDDQQNPVMRPTK

472	KDB16501.1	40S ribosomal protein S16	32.824	16.066	18.8	PLSLVQPEILR QALVQFDR TLLVADNR
473	KDB15716.1	60S ribosomal protein L31	6.1755	13.956	10.6	AFATQAMGTSDVR
474	KDB11591.1	SEC14 cytosolic factor	6.408	49.713	2.5	TIELEAYEQSR
475	KDB19062.1	Autophagy-related protein 27	18.445	38.113	11.2	AKPTEECPNGSR DAALIWESYGPEKDGDVLR HPLDGPVK
476	KDB18714.1	Cell surface Cu-only superoxide dismutase ARB_03674	101.18	28.597	28.6	ATAGPGGQGVK EGTPGFIGDR GEDPPCDASEPQTCQIGDLSGK ITQDPFR LDGNIR QEYVDLYASLK RGEDPPCDASEPQTCQIGDLSGK SIVVHFK
477	KDB18611.1	WSC domain-containing protein ARB_07870	31.101	57.264	13.1	DADSLQVSLR IHQTGAVLPR LIDTVPSTVQLSEPIDIVDVKPYIDK LRDPQEFR SDFLEDMLAIQSGAVK
478	KDB18188.1	1,3-beta-glucanosyltransferase ARB_07487	35.464	54.209	13.4	AADCPAVNAK ASTSCSFK DIPLLQQLQTNVIR GNPEWNVDLLAHYQK LLSDAGIYVLSDLGNPQQSINR
479	KDB18085.1	Probable glutamate carboxypeptidase ARB_02390	93.948	83.718	14	APAHDVIPSIPSIPISYR AVDAGWKPLR EALPILR EDVIGEDPTTSLK FVGSAAPLLNQALR GSVMFLSVR HFLFEGGLDNR HLSHEQLR PGDPTTPGYPSK PGDPTTPGYPSKPGVPR
480	KDB17956.1	Secreted protein ARB_01864	31.657	73.156	10.6	DMIQEQWFTDALASK DVFLYVADVPFDK GPFTFDDNFIVAPFR MQPGAPNALYGPK TPIQLLGGHTHIR
481	KDB17570.1	Probable aldose 1-epimerase ARB_05372	79.625	56.452	20.4	DQYGIDR DSPGGVDTLHGGPDGWDWR GAMGPYDWR GGVNDFWSEPK IGVDSILVPTGDMLANK KGGVNDFWSEPK KQIYEPGGDAYVLQAK LHPHFGGVPGR QGPVATLSSAWSGIR QIYEPGGDAYVLQAK
482	KDB17539.1	Extracellular guanyl-specific ribonuclease ARB_00194	12.625	16.202	19.4	LAGVITHQGANGNGFLECNVK QYSTDAVAK
483	KDB16877.1	WSC domain-containing protein ARB_07870	300.8	98.079	42.4	ADTFKPVTHIGGTCAAFAGAR ATVGGYK DAHGPGPMGVPQPQNSPQTLLQQFDR DYDYFGTEYGR FADTVHPCSSETSGPGR GSADCGSSSCTFTSTVQGVGK GVVNGNAPLDSTDTFFDNK GYPLQDDVLFQAPQSCISGK HAAAEWLR IGGLDASLQYELDNGENTGPGHR KDAHGPGPMGVPQPQNSPQTLLQQFDR KFADTVHPCSSETSGPGR KGSADCGSSSCTFTSTVQGVGK MGFNAEEMIR NPLVVGNSVR SAFHDMSTANTYFK SCGGPGGPIVPFR SGVLTVVAAVR SLQFTGYIR SVAITYK TMSTLADPDTFK TTGLGADAIK TTLTFMGPYVTSR TWPSNVDELEEIMFQIK VDVIAGDK VPQPNSPVPHLENTVVPLR VTACGHTIGGVHAPEFPELMPPR VVTEYLSGTTR YTLFSADFHVPGGSPYQSR YVSCWSEGVGAR
484	KDB16539.1	ABC transporter G family member ARB_01379	26.26	98.96	3.4	DGSIDLSDFLDQAIR GPGGFECK SSAEFSDK
485	KDB15986.1	Lysophospholipase ARB_05919	137.49	66.981	28.4	ADGQPPAAALPNIGLAVSGGGYR AGFETTITDYWGR AMLNGAGALAAWDAR APLPLIVADGR DGNSNYVPDDVPR DPDWPVCAGCAILAR DTFLNLGLNTR GPSQYSLIQYYK IAIPGFDVDGYLGR LAAAGLSAQER LTLVDGGEDLQNVPYHPHLLR

						PAPYVPDYFGK TILDEVQAK
486	KDB15742.1	Uncharacterized secreted protein ARB_07590	21.348	21.496	11.4	HVDYAIQLK IHGISLLGSGCPEK
487	KDB15273.1	Probable thioredoxin reductase ARB_06224	97.799	43.963	37.2	ANPGWQK DLIPDTPGLR ETAYAETGEVAR IILGTGIR IQPQQNNAYFTVTSK LAVDSTK LPSQPEHYLFR NAFLTSFPNEQYSTLGQDLGVQMVSGR NALTLNK SIEVLEK VLLIDSGVYR VNFDSGEPLLR YMHDVLGFDGVKPDYFR
488	KDB15211.1	Probable phosphoglycerate mutase ARB_03491	6.5852	42.992	3.4	MPHFETYYSSPLR
489	KDB14878.1	WSC domain-containing protein ARB_07867	252.58	118.5	26.5	AANAGNVLAGVEYGGECYCGK ALSQVFSVPGAEMTVAK DPNLNALLYDPTKPLGSR ECYCGDPANIDAR IGGDPAQLGNWPK ILDENNFSTVK ITLELGSAAR LYHSEAITLLDGR LYTPDGSAGVPGK NDWEENAAVLK NGAISASLLGSVSSTHGNSMGGR PLTMEWLQR PRPTFAIDNK PTFAIDNK QINVGGWSGDSTYGVR SDAECNTPCGGNPQQNCGGGGR TINQADTADAQMTNQK TLFPAIACSGTSCTVTSPPGK TLPNIPGAVNDPMGGR VLVSGSDPQDGVNPEEYR YTIAGAEYSR
490	KDB14703.1	Probable serine carboxypeptidase ARB_06414	181.59	63.431	32.5	ALHAPLDIQWGECVDTPFVQYK ASNSFLNDK DGCGNLWDFVFR DMSAPPSLR ELPNVIDK FYGSTFAEK KYAVDGAK LPDVDFDLGPSFSGLMPISNK MGFQSR PSAPFYVPR SGVLGTTHEER SLSDTEPFTFPQLR VTIHNEDELAQQFMGFWK YAVDGAK YGYDFNGNK YYAYPPAGPQPSSTPWTHK
491	KDB14638.1	Glutathione hydrolase proenzyme	21.434	42.149	5.8	LVDASHVR RLDDASFEAASEPR
492	KDB14361.1	Probable glucan endo-1,3-beta-glucosidase ARB_02077	19.979	173.74	2.1	DSLASWYQGVAAR GADTCLCGEK SRPLTGPIWTR
493	KDB14275.1	Glutathione hydrolase proenzyme	11.547	60.399	4.4	NPISINYR RPLSSISPLIAETPDGK
494	KDB13612.1; KDB15373.1	Uncharacterized secreted glycosidase ARB_07629	214.31	87.084	36.4	AEPGYFGITLQSGPQVEMTTTQHTALFR ASTQVHDLTISR DAEAAWR DAEVEPYDWCCR DLPPSLSR ELVLTLGGK EMPNFDFSK FANADKPVYAR GFGTMTR GFLQPR GGIDSWNR GYTQGGSNADVVLADVFQK KDAEVEPYDWCCR LSPITVNTEGVDVGFVK NVNFDPTYK NWIDHSFFTEGK PLILQDLSDLSDSR RPNGVDTGFK SFYSGIYR SINGYPLPGGAFVR SLISTYEHDGWLPDCR SQLPFLTILDPEAVAQMVR TVEYAYNDFCIAQMAK VDPQTGR YIASSGNWR
495	KDB13443.1	Endo-1,3(4)-beta-glucanase ARB_04519	51.11	56.142	12.9	DIQAEDPNPK DWGTPLAR FVSADCGAHGTSAGCGQR GFVEYVDSNTANK GGIFMGVDYK NAITLHTK
496	KDB13423.1	Probable extracellular glycosidase ARB_05253	61.569	40.945	25.9	DTSGTWQSIEAVGGGAVNSK ELPAAQAAPDQYPQTPMQVK FGAWAGGDPAYNAPGTVAWAR GPTDFAK GQLHAVPGTQAR SVVVADYSTGK WITYAVDWTADR
497	KDB12547.1	Probable 6-phosphogluconolactonase	134.4	40.251	36.7	ADDSIASFR ASHYLVVCEMR DDAFAVVNHVPVAPAGCGPR DPSGRFFAVNDLGTDR

		ARB_02015				FFAVNDLGTDR HGAFYPAGAAR ILVLDGR LELLGLTPTGGR LVLSDAAGHIYLADFDGAAFR NLVNLYAVR NVYVSNR QDKPHPHQTVR RADDSIASFR VVPAGASVK
498	KDB12181.1	Allergen Asp f 7 homolog	13.193	33.478	5.7	ELYGSLTSGR STTAIVADK
499	KDB12077.1	Probable glucan endo-1,3-beta-glucosidase ARB_02077	323.01	80.426	40.9	AAGSSTWWLANIPR ATILAGNALISSWAQGR GDGSTDDTVAINNAITDGQR GVLIESSK IDLTGLPK IGGSAGTQLQSDK IVGEIWPLILAGGNSNFK NNFCAAIAAFES NVPVAIQNNDNK PAALTDSTGR PIIQYYYTQLVGDAVSVPTLK PKPVFQVGR PQYNDVAASSFVSVKSK PVFQVGR SKPQYNDVAASSFVSVK SSITMVTLNGQATAK STGTGIHWQVAQATSLQNIEFNMIQDR SVPKPAALTDSTGR SYTGATGQAGQGPR TDDTAAIQAVFNNVGPNQVVYFDHGAYVVTDTVR TPNVQTR VEMFGISTK YSDPDFSHCSGAK
500	KDB11931.1	Metallocarboxypeptidase A-like protein ARB_03789	22.279	46.359	7.8	GAVDAIYAVNK NLQPNPGSSCR VPVADDGSNIR
501	KDB11780.1	Probable extracellular glycosidase ARB_03382	16.875	56.928	7.8	DSIGWYIDGAQVR SAQVTDFSSGQEYTYGDR TFATTNYFGK
502	KDB11503.1	1,3-beta-glucanosyltransferase ARB_07487	312.67	58.411	29.7	AVEESSYAPSNK DSPQWNTK DVPLLQELGTNIIR KYGDIFSFICTK LAFVLDAYYNSQNK LDDNWQANSILPPAPDQK LKDFDALK LLQDAGIYVISDLSEPDNSINR RDVPLLQELGTNIIR TYVDPLADEK WLGVGYAANDDK WLGVGYAANDDKDIR YGDIFSFICTK YTAVIDELAQYTNVVGFFAGNEVSNAK
503	KDB11462.1	LysM domain-containing protein ARB_01155/01156	23.894	26.462	11.8	KLNPQLDEK LNPQLDEK PASVVSGTTYK YAIQFSQLK
504	KDB11288.1	Probable pathogenesis-related protein ARB_02861	48.024	33.941	10.6	HDMNQGTGHYGQNLASAGSSANIDNLK VGCSTVK
505	KDB11015.1	Secreted protein ARB_01864	54.89	82.896	13	DSTYIVSPFVSGFNYIPDVPYR EPWFQDVIR GADLLVVDTGDR LAIMNTGGIR SLAIASGR YFETIGWMSIDGIK YIASNLDYLDPSTGEQRPVAQR
506	KDB10952.1	Probable extracellular serine carboxypeptidase	102.64	69.499	25.8	AAGPHAIGLPGR ATGGVGVVLEHR DGGPVIILHSGEADGNER EVDMVVGWLK FLTTDQAMADTAYFSNNFR LAFIDGR MLNYIGLVR MPFLEHGIVPLLAR QVVDMQR SAVTAAGYGSEPLTSR YAPHSDAFFNLR YWVDATHYK YYGTSLPLPLDEAATEGYR
507	KDB18682.1	Dimethylglycine oxidase	30.085	90.501	6.2	ALAEVLTTGK EASAAQAPVQVR LDFTPDDFR VGGLELATTPER YQDQDLYYR
508	KDB18659.1	Berberine bridge enzyme-like 18	12.844	68.298	4.1	ILQPTGIEDIQSAVR SFGLLGDYVR
509	KDB12278.1	Putative polyol transporter 1	12.287	70.23	3.7	IPRPPPIIEK QSAALNLIENVLK
510	KDB11568.1	Pre-mRNA cleavage factor Im 25 kDa subunit 2	10.386	8.4556	19.5	LLAVPLFELYDNTAR
511	KDB11072.1	Stress-response A/B barrel domain-containing	73.628	12.431	86.8	AHHAFVDR AMVVDFAPGSF DFYVNEDK DVSIEGLQNGFTHAFVVEFASAADR

		protein HS1				HIVLFGFKPDASPDSIK LKEQCVLASTGETYITASSGGK SADGIAAK
512	KDB17822.1	60S acidic ribosomal protein P2	44.207	11.017	30.4	DISELIAAGSEK LLSELEGK NVLSAVGIEADDER
513	KDB16568.1	Minor allergen Alt a 7	11.611	21.457	14.2	AFPQLTDMSAVR GGSPWGAGTFAGGDGSR
514	KDB12412.1	60S acidic ribosomal protein P1	7.0429	10.985	15.6	AAGIQDVEPIWTSIFAK
515	KDB13104.1	Actin, gamma	82.337	41.593	36.8	AGFAGDDAPR APEALFQPSVLGLESGGIHVTTFNSVMK AVFPSIVGR GYAFSTTAER IIAPPER IWHHTFYNELR QEYDESGPSIVHR SYELPDGQVITIGNER VAPEEHPVLLTEAPINPK YPIEHGVVTNWDDMEK
516	KDB15688.1	Putative lipocalin R877	19.958	22.42	12.7	LGSITGVATLADAR TGVFQVHFPHQR
517	KDB18918.1	Profilin-2	17.059	9.1994	28.4	AFADGLYIAGNR VISAIVSGEDAAK
518	KDB19097.1	Glucose repressible protein Grg1	49.419	7.389	12.7	DSNVSAGTR
519	KDB18984.1	CBM6-containing protein	208.53	39.932	55.4	APATLNVVLR DAVTNHYSR DEHGIVMLSSDGK GYAFPYDDVGPSSGEDQSGFVNDPDPQELTVCVGR IAAALNR ICHLVSVQGR LAFDGVGSFDK NETGASQLYAHITGR QPEGETLGSYYK SPSGANMR STLLANSR TPHHPPSPSDTLQPVGADCAIAVGAHGASR TVAGLPSDGLDR TVSIGR VCDALADQAR YASEDLTVNTQFR
520	KDB18948.1	PI31 proteasome regulator	9.2668	48.216	2.5	DVVQSSNLPVR
521	KDB18937.1	Putative gamma interferon inducibly sosomalthiol reductase (GILT)	29.717	30.838	15.6	ALIEDCALEHAIDIR DDGAYGMDLLR DYEHIPER LLILPVMQR
522	KDB18886.1	ATP synthase f0	67.919	32.966	10.1	ANLDAWNPFGR IWDQNALYR RSPPADAER
523	KDB18863.1	periplasmic binding ii	131.82	30.422	47.3	AGVLTEGK HFSLDANLVSFPSGTGHMITALR HVGDIYTPWSSWQVVASTK KYYDSGEIR LASGSAVDLR LEIASVDSLK NAVNSGEADFFMWEYFTSK RLEIASVDSLK TVNFTTK YFIENQEEAVK YISTELGYTEPDAR YYDSGEIR
524	KDB18796.1	DNase1 protein	14.474	17.957	19.2	TIHFTPNAGLPNIK TYFDVSGIVNADDVNNVK
525	KDB18783.1	hypothetical protein UV8b_174	32.212	60.336	10.4	AHNPQAFDGDGYWAR AIVSWVWPTSDSLGR FGSAVANPPVR GLSVESVDR RLDDLAR
526	KDB18778.1	XPG I-region protein	6.4301	81.406	1.4	EQALGLMIPR
527	KDB18758.1	glycoside hydrolase	29.318	43.303	11.5	DPFTYDFTIPK DVDSFGGAAMDAFK EMAAGNYTLAWTWFNR SIHGGCPAK
528	KDB18726.1	hypothetical protein UV8b_117	323.31	26.299	70.6	AMPENSLK DDVNIPFHAEDAAYFNFETLVPDSK DDVNIPFHAEDAAYFNFETLVPDSKR DPSCATVAHNIGVATGK EQLPCGAYPK EQLPCGAYPKR FDGQADAGK HGPLLVAALYIPGSTAANHK PATYEQVLSYAK QEATAWYIQTK QYYNQIK RDPSCATVAHNIGVATGK STNKDDVNIPFHAEDAAYFNFETLVPDSK STNKDDVNIPFHAEDAAYFNFETLVPDSKR TRYPSGSILAVWGR VYASTVPR WADEDQPTWHAEFTLGPFDPK YPSGSILAVWGR
529	KDB18655.1	haloalkanoic acid dehalogenase	14.692	26.435	9.2	FDTLGDMASALER LVILSNVDR
530	KDB18588.1	hypothetical protein UV8b_830	35.073	18.513	43.7	IDLHFPLGMEVTAR IPLTKPPVK RADDELENPYLAGFEWDKEESWTR SSTTVEDSSILK

						VDSAVQGLSSSPPK
531	KDB18396.1	CFEM domain containing protein	25.146	27.644	11.4	AQELGCNQGDLK DCSGAICSPQDAAK VVDYGVK
532	KDB18368.1	UDP-galactopyranose mutase	97.977	59.268	28.9	AFEHGYPTPSLER AIDGLIDAAMECR DFDQWILR GERPEYIGDK ILGEGIADMFMR IVTFADGSTVGYGK LINTMAVDDLAEK LPTLYLADGTK LQDMGIYSR MQCEWLGER QDDWYTHQR VSIQGLVNTEMLKPEDQIVSTYHR VWAVPTTK
533	KDB18301.1	hypothetical protein UV8b_1149	27.484	34.388	7.5	AKDPVQTQSLR DPVQTQSLR FVNNVSADQELPR
534	KDB18277.1	putative capsule associated protein	6.348	76.828	1.5	TVEQAAQAYR
535	KDB18266.1	hypothetical protein UV8b_1114	84.497	19.592	53.1	FVGVGEVR GLLHIDQEWVCER GSLADPGDK MLDFDFHSWR QGQLYEAASVQCGK TPDFFYAGDVYECR TSFSFDR VCTSLSGSTVSWK YSNDHWK
536	KDB18259.1	hydrophobic surface binding protein A	250.77	26.175	31	ALIDYIGNTIQPSMR DKIDADFNDAIAYFSS IDADFNDAIAYFSS KPQFDADQLTGVVHDTLQSLQSDTDK KPQFDADQLTGVVHDTLQSLQSDTDKLADALIK LADALIK LGTISTADAK PQFDADQLTGVVHDTLQSLQSDTDK SKKPQFDADQLTGVVHDTLQSLQSDTDK
537	KDB18258.1	circumsporozoite protein	51.205	66.083	14.3	ATIQSIQK AVVDAGNSPDLTFNQGLALVAPIGTLTK DDASISDDISAIVAR LPSSLQSLAPR LSQPIIDSLQK TNALNTVINNYQGAAQNPALFDSAR
538	KDB18211.1	hypothetical protein UV8b_1059	36.108	17.045	36.2	ALSGMDHNDAAMFESAFAGQDATMELR DEDNASLTAVAMAQHAPSGR LDTTHLMSNVR
539	KDB18206.1	outer membrane autotransporter barrel protein	157.02	38.875	57.1	AGVTVGLTEENTAR AHIHFDYSPTNAAVPFTDDVSTAVR DVAFTNAK DWDQVLGAVAIDGSSLFVGK EHDGMPQGTPDK GQDEGIVPSSVR ILYSGHWIPRLDVRPGAADAR NAPHLNTGR NPPGWEFLSSSK QVVVAR SIGGLDVAGSPDFR TKPPVLDDASFVELR VLGVGFGR VPLLAVESK VTLADPEK WETAFDCDGGFFGLR
540	KDB18126.1	hypothetical protein UV8b_974	64.327	23.039	22.6	GQIFSVDDWNVIVR LISQCFEPDGDVVK NIDCNYGDWADR PGFGPGR
541	KDB17951.1	cnd01770-like protein	8.027	16.665	7.4	DAGSNEIVYYR
542	KDB17882.1	oligomeric golgi complex component	6.3676	89.252	1	LLENTDQR
543	KDB17807.1	WSC domain containing protein	62.946	58.404	17.8	DHLAFPDLVMTGNCPDGYPVR HFLPEQTYLDANCADGLR LKPSLPQLLFGEDVIGPMAK NPEGTLYR SQWASLGQTTQSILAER SYTAGDPSQPDPPK
544	KDB17670.1	small secreted protein	70.153	17.163	47.2	AGLISFPAYR ATEITYVVEGPGASR DFDVYYLDSK GFTALSAIDR VLQPAEIPQSLAQIEAQFNPAPVPK VVADKDFDVYYLDSK
545	KDB17630.1	hypothetical protein UV8b_1514	17.914	16.764	15.6	GPFTIGDK QFSEGTELR TLLGTSLR
546	KDB17579.1	WSC domain containing protein	7.0468	41.154	2.6	SLWSDGWSFK
547	KDB17565.1	hypothetical protein UV8b_1449	49.667	30.871	24.4	AQEMLELMSR DDQVQMWGQTTR IGVLGGGAGSGSSK LDDVEQR QQLLVTMIEK RDDQVQMWGQTTR VWTELPMLGPVMR
548	KDB17496.1	suaprga1-like protein	17.558	32.857	11.1	AADGETDEELSAK IESPEYAQK LLDTPGQEVVK
549	KDB17424.1	hypothetical protein UV8b_1681	72.39	20.103	41.1	ATTVDQWSVK FWAQGAANLTLK LTQTWTCADEGSR MGQIYSSR STQPSCSDR

						TVSCDLVDATVPITSK TWTNPDWK
550	KDB17335.1; KDB17334.1	NIMA-interacting protein TinC	38.739	101.85	6.9	AGIMTSAK DHFSNILNEIAGR HGDIEDALLTLAMAR HGGQVEYVSQESR LEGQNWR NLMFDEVR
551	KDB17333.1	high mobility group protein	67.979	34.8	36.5	EDPAAEAPVDDHDAIEEQLQDEAK GAVQEEGQR GWNAAYQYNLR LSTILELLR NMSDEEALK SFTQDYIR SIIANDLGAEAPK VVDNESFLR WANMSPTEK YADDYQIPMPSIK
552	KDB17005.1	glutathione s-transferase	6.3761	30.804	4.3	DALTGLAELFAR
553	KDB16884.1	hypothetical protein UV8b_2475	12.023	48.943	5.5	DCPLAAPAPK SFAEAFDPLNPAYVHK
554	KDB16867.1	glucose repressible protein Grg1	6.8253	7.2239	13	DSDASIGTR
555	KDB16859.1	calmodulin	70.337	16.039	4.2	VAAVLR
556	KDB16821.1	NAP family protein	11.32	34.683	11.1	APPEIDEYIQPTDAAVIASLK DLTNGLLSLANK
557	KDB16672.1	immunogenic protein	142.02	54.276	31	AQGASFPK AVSTAER EFFFFGQDAVDAK ELAASVTESETYK GLLFIGDK KPAEVKPAEEPAK NVLGSLKPAPK SEEKPAEEAVK TAPSSVINLAEATEPENDGSNK TEEPAPLQPVEEGHLAHK TEESPAVEGETAAAAAAADDGKPVEESK TSIFGFVK
558	KDB16608.1	hypothetical protein UV8b_2643	123.96	37.908	36	DDYAEGFQLLQGDLDK FQSLLDALGK GVFEPQGHVAAHWVPAVR IGAYLHAR NHIGVGGPDLVPENAQGHPPYAGAMK SFEFYAR SLESAKDDYAEGFQLLQGDLDK TFAVQYVNYWPGANGNDDIVAAR VWIQIQDR
559	KDB16456.1	hypothetical protein UV8b_2912	31.906	16.069	26.4	LYGEQGCSQK NEGVWTVIDDDFR NVHVADMR SLNGLPAR
560	KDB16389.1	hypothetical protein UV8b_2845	16.429	20.266	4.8	CQQLPPPFR
561	KDB16205.1; KDB14769.1	carbohydrate-binding module family 48 protein; hypothetical protein UV8b_4292	24.748	45.376	5.3	TVSISPLPATAGIGNPIK VPVVVR
562	KDB16030.1	endo-N-acetyl-beta-D-glucosaminidase precursor	7.0206	38.843	2.8	VVAGQLTSPR
563	KDB16017.1	putative 4-carboxymuconolactone decarboxylase	36.987	18.742	32.7	HHAPLAAEAGVSK NVQVGDETFR QSEKPAELSEK QWAVLVYADEMTR TSLGADLR
564	KDB15993.1	extracellular membrane 8-cysteine cfem	18.782	16.7	25	CSVTDYACVCEK TNFAAVQGDATSCVIASCGADVAVSQVLPAVQK
565	KDB15977.1	copper resistance protein Crd2	15.787	11.179	20	ARPAGQCTCDR ASSENTVQGPR
566	KDB15962.1	hypothetical protein UV8b_3274	84.952	17.211	50	CSAKPDYSWALIPYDGHDFK DTVDKDGLTK FNLQLGNQIYACDVHGGVSFPMDTK GVYFSGTGAMPTNCDK PDYSWALIPYDGHDFK YALQIHHYR
567	KDB15881.1	extracellular matrix protein precursor	43.496	22.477	27.9	FDSCQSGCTIVLQNGPQTNTK FLNSNFQVVAGQPFTLK VLTSDATGGAFTFTPSGWASDTYNFK
568	KDB15874.1	cell wall protein	58.982	19.414	22.3	AQSVAAAQCK HIGCTGSPAEIAVCACKPETQSMLVSPVTQCAVSSK PETQSMLVSPVTQCAVSSK
569	KDB15806.1	wall-associated proteinase precursor	102.15	37.385	45.1	DWAGLSAYLDTPR EATFQAIQHPWK IGEWVLSEQGPK KFDVSESDTR LLSANAVLGGK

						MAAVTVIANPLAK MNYLDLADFSPDTNLYAVYDEAQK SLIDFNAR SSDDSETVVK TITFEEPEEQPR VMSNTPLAVTIIPGNEEK VVVFGLTTK
570	KDB15803.1	hypothetical protein UV8b_3443	26.746	41.87	10.5	DLASIADYLR ESTELNCYGGPLAR LYSMTSR NPIYWSDEYR
571	KDB15770.1	hypothetical protein UV8b_3410	23.079	11.004	25.7	DAPPVPPGCSQR GCYSDPIHPSCPPDR
572	KDB15756.1	hypothetical protein UV8b_3396	6.1867	6.9759	14.1	GASLNIYNR
573	KDB15744.1	serine threonine protein kinase	16.143	80.44	3.6	GQPATIAPSSITPPTK SEEATGEMNNDK
574	KDB15684.1	carbohydrate-binding module family 19 protein	11.28	71.958	3.6	LGCSDPAEAQR LYSSLTPQSACAER
575	KDB15409.1	hypothetical protein UV8b_3835	109.23	23.425	17.4	AAAALAASAAATVETIAVQR LVPPVR VTVDVDAPYAAVVER
576	KDB15364.1	hypothetical protein UV8b_3790	12.98	23.228	8.1	AEGCCPAGR GSCDGGLVACGR
577	KDB15312.1	indoleamine 2,3-dioxygenase-like protein	11.677	88.945	2.1	GFALALDR HVSEAHDVR
578	KDB15298.1	ECM33-like protein	120.57	40.743	26.5	DSLTLDNNKK INQFPELK LNMFDSK LQGLQLLSSFDMK MNSLENAGELDFR NLELINLNQLSGLSLGTSGVTK SFDAPILGTASR SIGSVLLAGSFNTVEIPK
579	KDB15293.1	hypothetical protein UV8b_3719	67.663	410	3.7	AATAAAPSSNSPEPVK AEDPAESVK AQDDSAPEK EQASESVEK LAEEEKPVDDSPVEESPK QSETPVVEPAASEPR SESEDAPDADETK TEDEPDTTMAAPSK VVEAQGMPADEAVSTDSAHETGAILSSVSDSGDVDVDVDR
580	KDB15258.1	allergen asp f4-like protein	12.343	29.414	10.5	DQTCVVFLK VLAAEANSQGGAACSPGDAVK
581	KDB15214.1	glutathione S-transferase-like protein	17.332	26.243	9.5	IQLGPPSEALAR LTALFEEGLER
582	KDB15125.1	cell surface flocculin	25.243	93.464	4.2	IILPDDPNK IILPDDPNKPVPDGTVPIQIAFR YLPEALSGASGLPANK
583	KDB15119.1	heterokaryon incompatibility protein	7.1303	84.633	1.6	LVGTAEIEPGTR
584	KDB15099.1	hypothetical protein UV8b_3939	6.3814	116.04	1.1	EAPATTAFNAAR
585	KDB14950.1	malate dehydrogenase	62.298	26.135	28.4	GAVNLLAK GPDGTGAVPWLQLGAK HSLSQTAWDALTSR KLAAVDAPR LAAVDAPR SADKGPDGTGAVPWLQLGAK TAGATPTSTGALAMLYDITK
586	KDB14800.1	hypothetical protein UV8b_4323	63.921	19.47	24.9	CVAACPQGNGSPADTQK GDVNCQSHCITVPSPNEEQVIETTK YADCSNACIK
587	KDB14781.1	putative tubulin-specific chaperone Rbl2	26.293	12.568	34.5	EEASYHTELGSQEAEIAR PAPSQLAIASGSVTR TALEQTR
588	KDB14767.1	TPR domain protein	17.318	76.379	4.7	ADLGIDDSLPR AVAANTEAITADEK VADVPVR
589	KDB14624.1	hypothetical protein UV8b_4480	26.167	17.91	26.9	AASQAAFAR IDNNLQTVIR QAALDAVAK VWTGDYTGAVALISQASK
590	KDB14482.1	hypothetical protein UV8b_4753	7.9244	31.749	3.7	LGTFCAISPTR
591	KDB14378.1	ester cyclase	40.502	15.036	31.6	CMEHMFYEFR EDFEALVAEDVDAAPDLK FTVVMPVVDESTQR
592	KDB14352.1	putative restin	49.462	118.8	7.2	ELEAEVER ELSQHLAQEK GDLELAQMELEAHK IDLAAAEGSK LADAQDSLSAIK QEDGVAASELSLK SAKPDTSEADALR
593	KDB14349.1	hypothetical protein UV8b_4944	14.144	16.461	15.5	SDTTKPAEPADAK TDTTAVSAEVNK
594	KDB14310.1	extracellular serine-rich protein	12.541	19.845	10.1	NVFQIVVR SVIPPVSDVGK
595	KDB14294.1	glycoside catalytic core	49.217	34.574	23	GLCFTPNPSYPDDNK HVLGFNEPDTIHSYGGSNVKPADAALAWVANFEPLGK LDYIGR

						LIDQGTK PADAALAWVANFEPLGK YSYFGAFR
596	KDB14287.1	methyltransferase type 11	268.89	30.906	76.7	AARPDDKPFNLPIPPEWFSADHVK AVLEDGGFAQVQLVR EAVLEHLAK PPSAIDFFDMAAPK GVIQPAQK IAALGAQAGCK KPAHFGAPALPELR LAVMPAEK LDFPDATFTHSITNLGILFFADAPAAAK MLAIAQDK PAHFGAPALPELR PDDKPFNLPIPPEWFSADHVK PFNLPIPPEWFSADHVK RTGAPLPQIHAVDPAK TGAPLPQIHAVDPAK TLKPGGVAVVTSWSDMGYVK TWSEDEQVK VTVPYTTPSGEPGVGVPMEAIIAVCR YEWASGSSTR
597	KDB14199.1	hypothetical protein UV8b_4989	76.437	25.161	45.7	ADLPPSSK DVAAIDWAK DPLPEGSFSNHDFAYETSCYK LTNAIPPNCVPVR LVAQCTK QFDLGLK STVVSDIGTPLSPWGVFFTNDEGSDMAHTAHLDGGPGAK
598	KDB13988.1	agglutinin-like protein ALS2 fragment	58.523	193.64	4.4	GFAVGATVSPQQPLK LEAGQVCFPSTVQLR NIQVSSDAVSGSHSAVNIR SVAAESTVER VDCQQTITVK VVASPVTPEFNENWTK
599	KDB13985.1	Kazal domain-containing protein	74.791	14.546	64.3	CGGFAGFACPK GLYCYYGPLTGCDPK LCPGTCR LPESCGLACDVPGICLPK SCKPGFECR TCVPNAGCPNPK TTSDCMGICL VAPCPTDK
600	KDB13907.1	hypothetical protein UV8b_5241	6.1723	22.083	4.5	TGQTDLSLR
601	KDB13881.1	hypothetical protein UV8b_5215	18.245	9.3785	46	AGPFVQQELGAVSQNLNSPETHYTYPCK SLSNPDCDFSGR
602	KDB13709.1	carbohydrate esterase family 9 protein	82.733	103.36	15.1	AEVEGLAAAR APASALFADNMFYK ATVVPGR GDVYLEHGLISK GTSDFNEFSDNVTPYVR LGESFAFR MEAYVGSESAGK SIDGLYVFDPQIQVIK SYTEAFGALR VGAQHGVTR VGQDLPLHSLPQDTQLFDAEGR WELLGAALR
603	KDB13686.1	hypothetical protein UV8b_5518	9.0031	17.797	7.8	GLTLDTTLAGVAK
604	KDB13670.1	hypothetical protein UV8b_5502	6.8604	53.699	2.3	SLADTVKSDVGK
605	KDB13669.1	fungal transcriptional regulatory protein	15.885	39.326	1.7	GLLTIR
606	KDB13614.1	IDI-2 precursor	43.182	13.752	36.1	CAEHPLAASPNPVSK NDGQCNTNQACGIGNCK VDSASLPPGVDAAAIR
607	KDB13604.1	hypothetical protein UV8b_5436	55.425	19.907	29.5	ALSTCHSGAAIESLCLLPGSK QNVQYVSFDSNDILNIVSYLDDTK VLDNWYICK
608	KDB13594.1	PCI domain-containing protein	6.6978	20.573	6.6	NIQALCATYDPPK
609	KDB13550.1	hypothetical protein UV8b_5648	6.3388	21.342	5.8	DHTDWLFGTVK
610	KDB13513.1	hypothetical protein UV8b_5611	25.478	43.228	10.9	IAWTNQDTQER LTAGLPTGDR SDLVSEEETIDKLTAGLPTGDR YQPQPGDYMR
611	KDB13349.1	hypothetical protein UV8b_5728	29.038	24.909	14.7	AHANYCWAPDVILR CDGNAVGTR FMCESGSGSGSGSACR
612	KDB13297.1	hypothetical protein UV8b_5887	8.1978	10.095	10.4	NAGDTVSDQGK
613	KDB13261.1	epididymal secretory protein E1 precursor	25.237	15.505	32	LWGVVGPAQVAFPLTPSDACGK MELVTDK STSPIESASIK SVPVEIK
614	KDB13234.1	thioredoxin-like protein	108.5	23.517	45.5	AADEESLNVGNR LAAGAGVDEAR LVGVHVTPTVVFDGVVQDTSSGWTVDEWK QQVQPWHPSSTLLHEAGLAVLR TLTTQVFPAVR VYALLEIPDK
615	KDB13205.1	hypothetical protein UV8b_5965	10.845	22.65	9.5	GTLCVDK YSQWTYPCAGVAYK
616	KDB13157.1	cystein rich protein	134.16	45.004	32.3	CTPGSPVNPSTQVYIR CVCPAGQEEGPDGHCK DTYGDLATGANK GQWLNGAQNGAHIGR

						IEYDDPDCDPGLECK KNIPTFEVGPGAK MCAAAGTSPTLSDDK MCAAAGTSPTLSDDKK NDNVYYAAPDSMIQR NIPTFEVGPGAK VLVDGK YFACCAAGQR
617	KDB12913.1	endonuclease exonuclease phosphatase family protein	100.08	32.984	52.6	ADAWACDASR DGELSPILFR DVAALAGPR GHLGNHVTYTSFGEPDEFPQR GWDAALNR LAAEWGRPDPDPGAPDAPDPVPVLVAGDFNSRPDGDAYR LAAQLR LDFVFVR SADLLLR TLWLSETPDR TLWLSETPDRPSR VMTAPGSGLR VVVMSTHLDHR YAADEHQR
618	KDB12896.1; KDB18170.1	hypothetical protein UV8b_6257	35.685	32.7	17.3	AGQVCFPSTVQLR APPNSVSSEALGK CSQQFTFR IWTPTPNFPFDK LLANEFR
619	KDB12618.1	ThiJ/PfpI family protein	7.9702	31.797	3.8	FDLLDLSGPLK
620	KDB12522.1	putative phosphoglycerate mutase family protein	184.27	18.873	74.3	FDEIWIDPHPYSR HGEKPSGDENGLSAQGK IMAQAYKPSGK ISDIVSEDCPGLDD KYDGPGNILICWEHK LGDENAPSYPSDR LGDENAPSYPSDRFDEIWIDPHPYSR MLTELAEK NVFAAGTSYNIGK PSGDENGLSAQGK RPYDTVK RPYDTVKPLADDLGLTVDVSCDR YDGPGNILICWEHK
621	KDB12517.1	hypothetical protein UV8b_6642	7.0466	20.606	11.5	IDQSGGAHDISYDAWNYLAFGK
622	KDB12370.1	aspartic endopeptidase	119.72	46.871	35.7	DIPFSV EIGLADPTVK FTFDGATPGTSVTIAVPIR GAAVFGAIPSTPK GIYDSDPGLDTSK LTASNLMGPGGK LTASNLMGPGGKPR NCLLDSGTGADR NTADGLSAIGPLTQIAAGK QTNPDPPYTGYYAALPK STTYSSVTNFPR SVETFFDWPK TSSSSFSVWMDQAPR
623	KDB12366.1	hypothetical protein UV8b_6729	23.945	58.332	9.4	DGAVIDFADGPGR FLVSESQQVPGEIVDYSAVAR GQVAPVASIAPINNEK
624	KDB12319.1	GPI anchored serine-threonine rich protein	30.882	25.315	13.3	LESNSSIFQYSNPFHIR TQNTQQHIADIASGIK
625	KDB12270.1	hypothetical protein UV8b_6869	60.719	24.789	50	EINWPYGTATAAHGWELQVAATVAR FSKPATGER GTVDSFVR KPPAHDAFNAPYLTHSK LSTVCTDNYSDWSVSDAPR NVDETGQSLWVYHVEGGGK QFDQAGILLK RVTVSVER
626	KDB12228.1	small secreted protein	12.27	16.045	12	LITYPSYTDK VIFEHVVR
627	KDB12199.1	putative cell wall glycosyl hydrolase YteR	20.515	43.6	12.6	AIAASLPLQK QGIQDSGAASSTLESGILSQALQEAIAR YLSDVLDIASSK
628	KDB12195.1	methyltransferase domain-containing protein	6.7034	30.36	5.2	FIVADLLNPGEALK
629	KDB12185.1	FAD dependent oxidoreductase	13.194	52.118	3.6	LLQEYPASR VAEMILGR
630	KDB12127.1	hypothetical protein UV8b_7030	13.623	43.599	6.1	APMAVNDDELR LNTQSSSQWDGLR
631	KDB12104.1	cell wall protein	13.535	23.734	9	ADALDDQVK AVDDVLADAEK
632	KDB12054.1	thioesterase family protein	7.0267	70.242	1.8	QVESLLATLLAR
633	KDB12037.1	V8-like Glu-specific endopeptidase	12.626	29.869	9.1	EHYCTASVVESTSR VHVQDVTGGNK
634	KDB12008.1	tropomyosin-1	69.448	17.982	49.4	AADEGQQHGTQNETLQR AEEASSQTEELK ALEQENLAK KVQALEQER LQLLEEEAEDADK NNLLESEVEK NNLLESEVEKLETAIK
635	KDB12006.1	ER protein BIG1	21.516	29.32	16	GQYSVAEVVGDVTGAR VASVIQVPLEPLSPDSR YLIVTQPGLR
636	KDB11957.1	ethyl tert-butyl ether degradation	46.007	11.095	39	FDSLAAWEAASTGPVFENLVADIPR FTAAQPLIISGESR
637	KDB11808.1	prp 4 CRoW domain-containing protein	7.2126	28.709	3.8	TTYGYNPDQQK

638	KDB11807.1	acyl binding protein	25.217	9.5956	48.3	EAQDEYVALVDK FSIGEEAPK PAKPFTPTDNEAFNQAVAAANK PFTPTDNEAFNQAVAAANK
639	KDB11794.1; KDB17599.1	glutathione s-transferase	26.895	20.667	18.2	ALGILDAALR LGALLR SLYPDLGPR VELSLGDGTR
640	KDB11784.1	cell wall protein	178.22	27.014	46.2	DAQTIAASLAAGLSK DLTESVVSK ITAAVDALETATR KSQNLADDFK KTVDGSQDLTLADSVGLTEPVTSLTK LAAIANGSK LGACDIVR NSGGDVSSLQAK SDALVAAILDGK SDALVAAILDGKK SDFSEDK SQNLADDFK TVDGSQDLTLADSVGLTEPVTSLTK VLEQSR
641	KDB11767.1	acyl-CoA thioesterase 2	79.131	29.681	45.9	ATSPATIDICSY DTCPPNSVSDFR HVSNLNPGWNWGK IIYYVYFK LSSGCPAQPR NADYQFWAR NNGGDSWVR QIGWGDINDTFDGFGDWQNFQQK SDWEGIVVSFR WHHSVHQDWHTNTFK
642	KDB11603.1	actin lateral binding protein	7.9504	14.332	11.1	ALDAADDAAEEVKR
643	KDB11589.1	putative glutathione S-transferase	30.021	25.229	23.4	AAGLPAGHWLLAMADDQLR AALDELDKR LADNAWLAGR PLIVHHLGISQSER
644	KDB11529.1	hypothetical protein UV8b_7674	64.954	19.377	52.3	ASGTEPLSTENNFK EALANADKDLINKR EAVESLEAILK GEQAPNAASFPEAR KGEQAPNAASFPEAR MDFITPFLTK STYTFYDASIPIIR VDETITIGLGPK
645	KDB11519.1	hypothetical protein UV8b_7664	11.442	54.488	4.8	AGDVSNEYGAIR DKLGSLFPNDAK
646	KDB11470.1	putative GPI anchored protein	59.132	24.092	27.5	AESGWQQIFPIVQQSGIGDFCEPQVTVPSK VSFTDDSR YPESIGFDDDKEDTAPCGGFTPDFSK
647	KDB11463.1	hypothetical protein UV8b_7686	62.106	35.201	23.8	NSHVSTGFLILMNGLR PGFPATYWVDAVTSVR QQQSTPYSNYGEEMMEAFQR SGDSCEALASSK VLTACAGDPQPR
648	KDB11336.1	hypothetical protein UV8b_7798	7.0654	30.831	5.1	GPTGGCGTGYTSCMNK
649	KDB11306.1	hypothetical protein UV8b_7835	20.987	52.129	7.3	DTIPQLLLPVTGQPAYSGNEVINK QSQELPCQSDIR
650	KDB11214.1	hypothetical protein UV8b_7947	81.406	14.292	30.8	GISVAGLGEVNAASICPPSYPR SCCQDWAK YCPVGGFCCR

[a] Number of protein

[b] Protein grouping number

[c] Protein name obtained from UniProt database

[d] Protein identification score

[e] Protein quality

[f] Sequence coverage of proteins

[g] The amino acid sequence matched by the identified protein
